# Supplementary material for: Antiparasitic Meroterpenoids Isolated from Memnoniella dichroa CF-080171
Source: Pharmaceutics. 2023 Feb 2;15(2):492. doi: 10.3390/pharmaceutics15020492 (PMC9962372; doi:10.3390/pharmaceutics15020492)
Supplement: Supplementary file 1 [file pharmaceutics-15-00492-s001.zip › pharmaceutics-2120655-supplementary.pdf]

Supplementary data

Antiparasitic Meroterpenoids Isolated from *Memnoniella dichroa* CF-080171

Frederick Boye Annang[1*], Guiomar Pérez-Moreno[2], Cristina Bosch-Navarrete[2], Victor González-Menéndez[1], Jesús Martín[1], Thomas A. Mackenzie[1], Maria C. Ramos[1], Luis M. Ruiz-Pérez[2], Olga Genilloud[1], Dolores González-Pacanowska[2], Francisca Vicente[1] and Fernando Reyes[1*]

[1] Fundación MEDINA, Centro de Excelencia en Investigación de Medicamentos Innovadores de Andalucía, Parque Tecnológico de Ciencias de la Salud, Avda. del Conocimiento 34, 18016 Granada, Spain

[2] Instituto de Parasitología y Biomedicina "López-Neyra", Consejo Superior de Investigaciones Científicas (CSIC) Avda. del Conocimiento 17, Armilla, 18016, Granada, Spain

Correspondence: freddie.annang@medinaandalucia.es (F.B.A.); fernando.reyes@medinaandalucia.es (F.R.);

Tel.: +34-958-993965 (F.R.)

Supporting Information Contents

Figure S1. Schematic of the bioassay-guided isolation process

Compound 1

Figure S2. UV/vis (DAD) spectrum **1**.

Figure S3. ESI-TOF spectra of **1**.

Figure S4. ^{1}H-NMR (500 MHz, CD_3OD) spectrum of **1**.

Figure S5. ^{13}C-NMR (125 MHz, CD_3OD) spectrum of **1**.

Figure S6. HSQC (CD_3OD) spectrum of **1**.

Figure S7. HMBC (CD_3OD) spectrum of **1**.

Figure S8. COSY (CD_3OD) spectrum of **1**.

Figure S9. NOESY (CD_3OD) spectrum of **1**.

Figure S10. JRES (CD_3OD) spectrum of **1**.

Figure S11. IR (MeOH) spectrum of **1**.

Compound 2

Figure S12. UV/vis (DAD) spectrum **2**.

Figure S13. ESI-TOF spectra of **2**.

Figure S14. ^{1}H-NMR (500 MHz, CD_3OD) spectrum of **2**.

Figure S15. ^{13}C-NMR (125 MHz, CD_3OD) spectrum of **2**.

Figure S16. HSQC (CD_3OD) spectrum of **2**.

Figure S17. HMBC (CD_3OD) spectrum of **2**.

Figure S18. COSY (CD_3OD) spectrum of **2**.

Figure S19. NOESY (CD_3OD) spectrum of **2**.

Figure S20. IR (MeOH) spectrum of **2**.

Compound 3

Figure S21. UV/vis (DAD) spectrum **3**.

Figure S22. ESI-TOF spectra of **3**.

Figure S23. ^{1}H-NMR (500 MHz, CD_3OD) spectrum of **3**.

Figure S24. ^{13}C-NMR (125 MHz, CD_3OD) spectrum of **3**.

Figure S25. HSQC (CD_3OD) spectrum of **3**.

Figure S26. HMBC (CD_3OD) spectrum of **3**.

Figure S27. COSY (CD_3OD) spectrum of **3**.

Figure S28. NOESY (CD_3OD) spectrum of **3**.

Figure S29. TOCSY (CD_3OD) spectrum of **3**.

Figure S30. ^{15}N-HMBC (CD_3OD) spectrum of **3**.

Figure S31. IR (MeOH) spectrum of **3**.

Compound 4

Figure S32. UV/vis (DAD) spectrum **4**.

Figure S33. ESI-TOF spectra of **4**.

Figure S34. ^{1}H-NMR (500 MHz, CD_3OD) spectrum of **4**.

Figure S35. ^{13}C-NMR (125 MHz, CD_3OD) spectrum of **4**.

Figure S36. HSQC (CD_3OD) spectrum of **4**.

Figure S37. HMBC (CD_3OD) spectrum of **4**.

Figure S38. COSY (CD_3OD) spectrum of **4**.

Figure S39. NOESY (CD_3OD) spectrum of **4**.

Figure S40. IR (MeOH) spectrum of **4**.

Compound 5

Figure S41. UV/vis (DAD) spectrum **5**.

Figure S42. ESI-TOF spectra of **5**.

Figure S43. ^{1}H-NMR (500 MHz, CD_3OD) spectrum of **5**.

Figure S44. ^{13}C-NMR (125 MHz, CD_3OD) spectrum of **5**.

Figure S45. HSQC (CD_3OD) spectrum of **5**.

Figure S46. HMBC (CD_3OD) spectrum of **5**.

Figure S47. COSY (CD_3OD) spectrum of **5**.

Figure S48. NOESY (CD_3OD) spectrum of **5**.

Figure S49. IR (MeOH) spectrum of **5**.

Compound 6

Figure S50. UV/vis (DAD) spectrum **6**.

Figure S51. ESI-TOF spectra of **6**.

Figure S52. ^{1}H-NMR (500 MHz, CD_3OD) spectrum of **6**.

Figure S53. ^{13}C-NMR (125 MHz, CD_3OD) spectrum of **6**.

Figure S54. HSQC (CD_3OD) spectrum of **6**.

Figure S55. HMBC (CD_3OD) spectrum of **6**.

Figure S56. COSY (CD_3OD) spectrum of **6**.

Figure S57. NOESY (CD_3OD) spectrum of **6**.

Figure S58. IR (MeOH) spectrum of **6**.

Compound 7

Figure S59. UV/vis (DAD) spectrum **7**.

Figure S60. ESI-TOF spectra of **7**.

Figure S61. ^{1}H-NMR (500 MHz, CD_3OD) spectrum of **7**.

Figure S62. ^{13}C-NMR (125 MHz, CD_3OD) spectrum of **7**.

Figure S63. HSQC (CD_3OD) spectrum of **7**.

Figure S64. HMBC (CD_3OD) spectrum of **7**.

Figure S65. COSY (CD_3OD) spectrum of **7**.

Figure S66. NOESY (CD_3OD) spectrum of **7**.

Figure S67. IR (MeOH) spectrum of **7**.

Compound 8

Figure S68. UV/vis (DAD) spectrum **8**.

Figure S69. ESI-TOF spectra of **8**.

Figure S70. ^{1}H-NMR (500 MHz, CD_3OD) spectrum of **8**.

Figure S71. ^{13}C-NMR (125 MHz, CD_3OD) spectrum of **8**.

Figure S72. HSQC (CD_3OD) spectrum of **8**.

Figure S73. HMBC (CD_3OD) spectrum of **8**.

Figure S74. COSY (CD_3OD) spectrum of **8**.

Figure S75. NOESY (CD_3OD) spectrum of **8**.

Figure S76. IR (MeOH) spectrum of **8**.

Figure S77. Picture of ***Memnoniella dichroa*** **CF-080171**.

Figure S78. EC50 curves in the cases of the compound which could be determined for the concentration ranges tested.

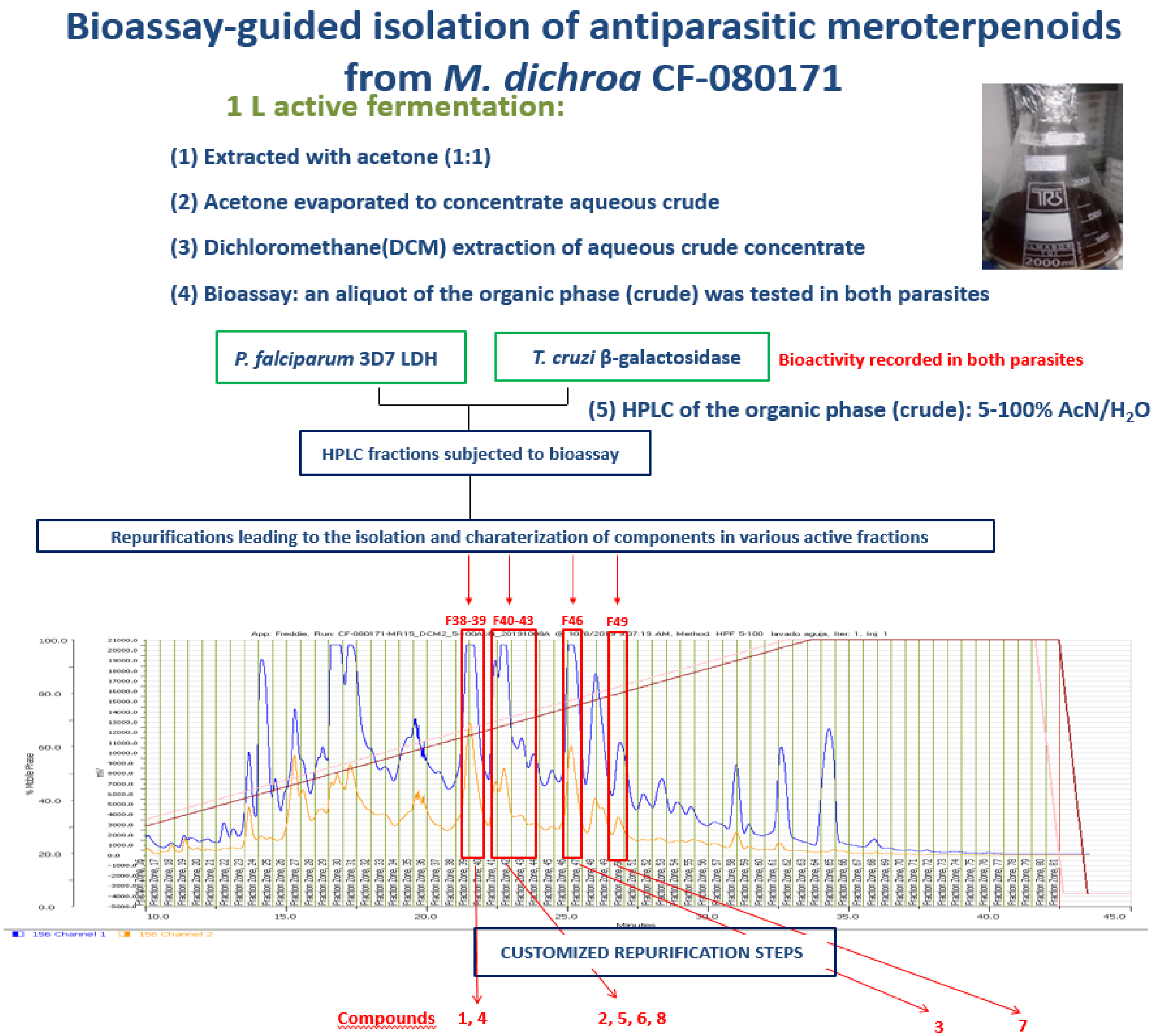

Figure S1. Schematic of the bioassay-guided isolation process

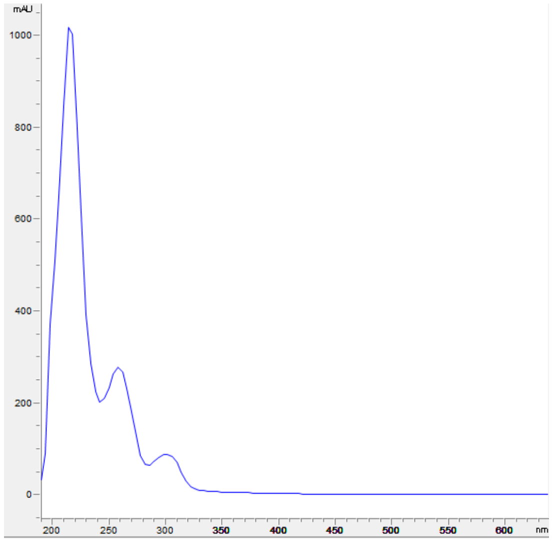

Figure S2. UV/vis (DAD) spectrum **1**

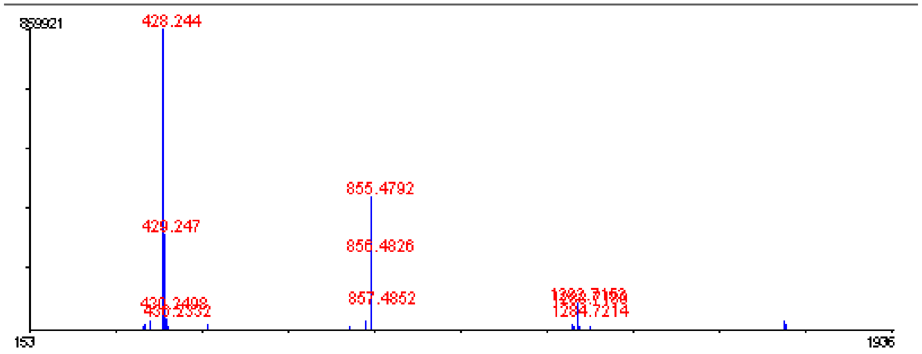

Calc. M+H^{+}= 428.2432

Calc. 2M+H^{+}= 855.4791

Figure S3. ESI-TOF spectra of **1**.

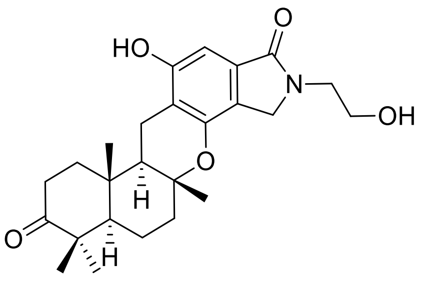

1

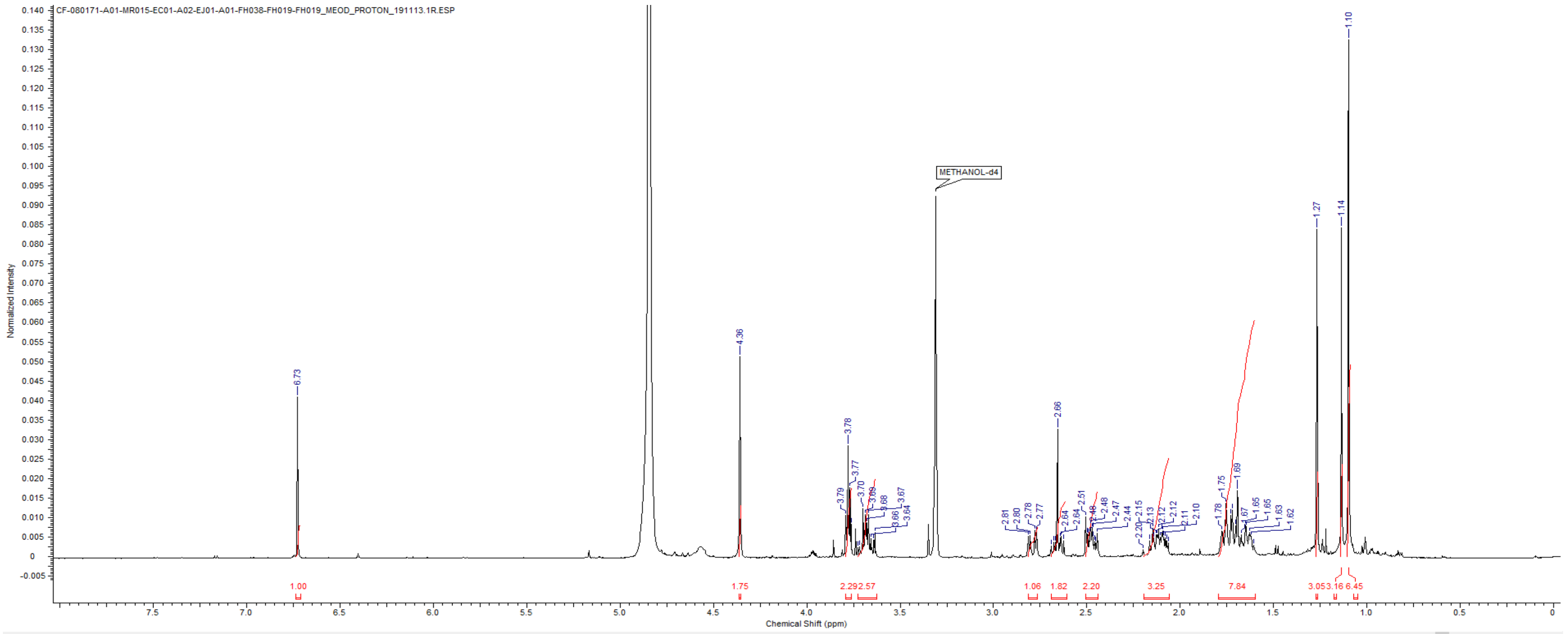

Figure S4. ^{1}H-NMR (500 MHz, CD_3OD) spectrum of **1**.

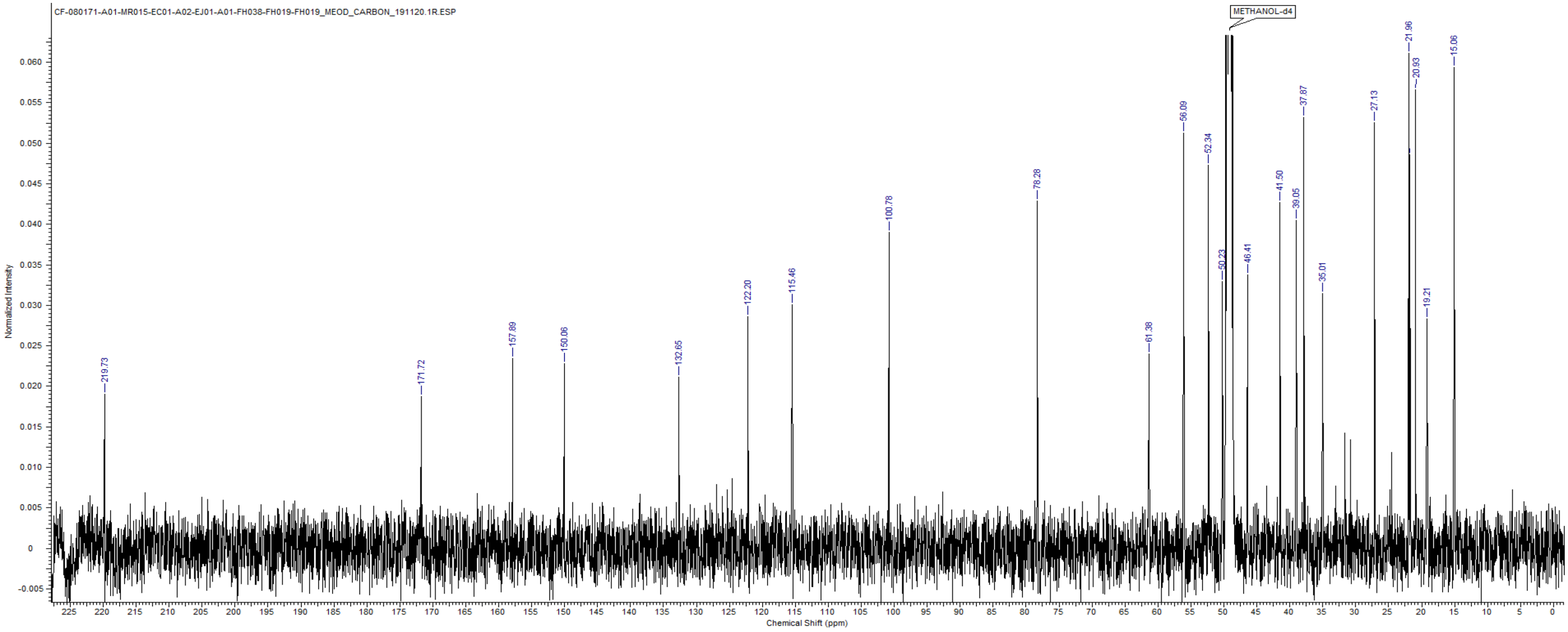

Figure S5. ^{13}C-NMR (125 MHz, CD_3OD) spectrum of **1**.

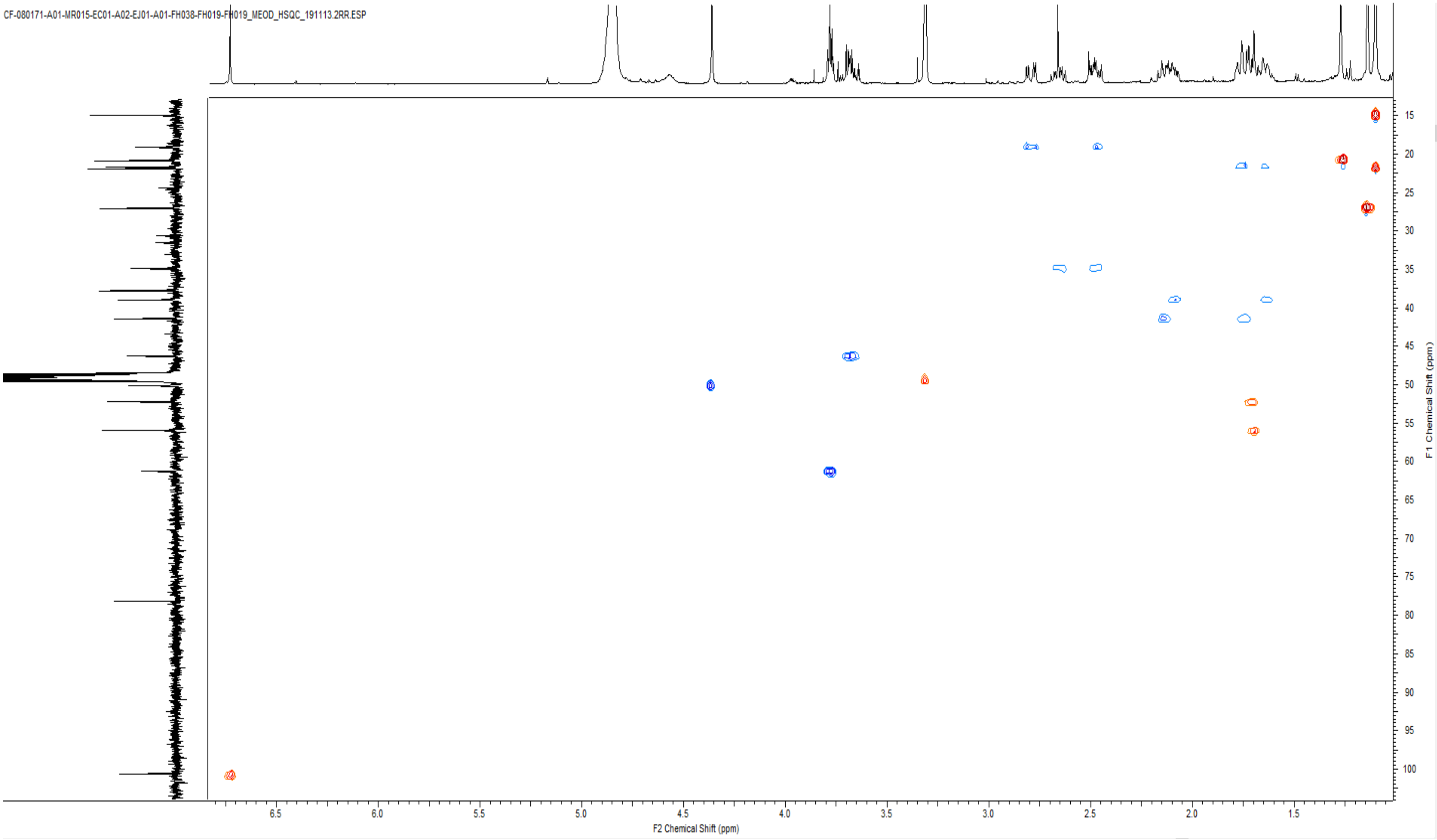

Figure S6. HSQC (CD_3OD) spectrum of **1**.

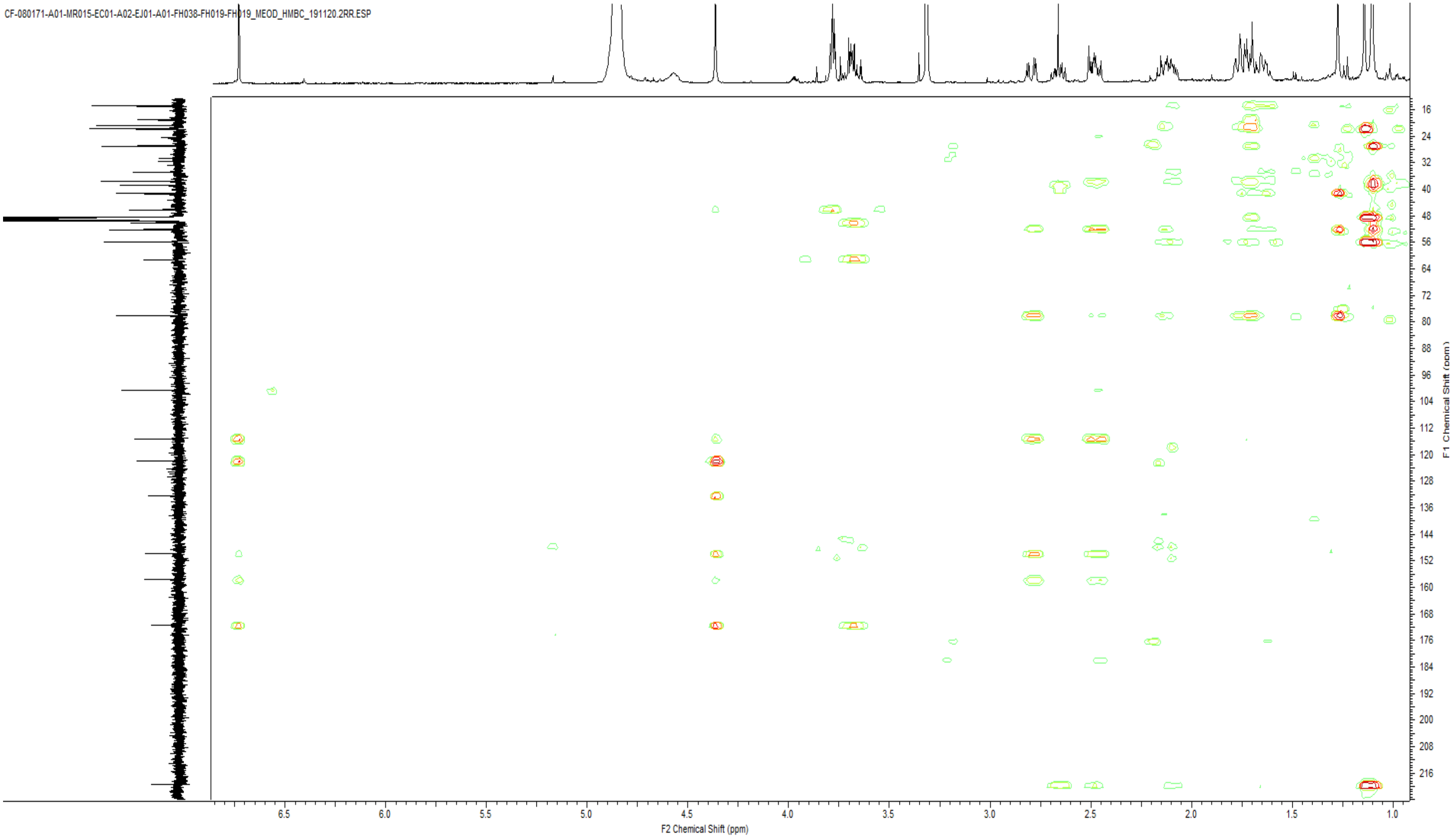

Figure S7. HMBC (CD_3OD) spectrum of **1**.

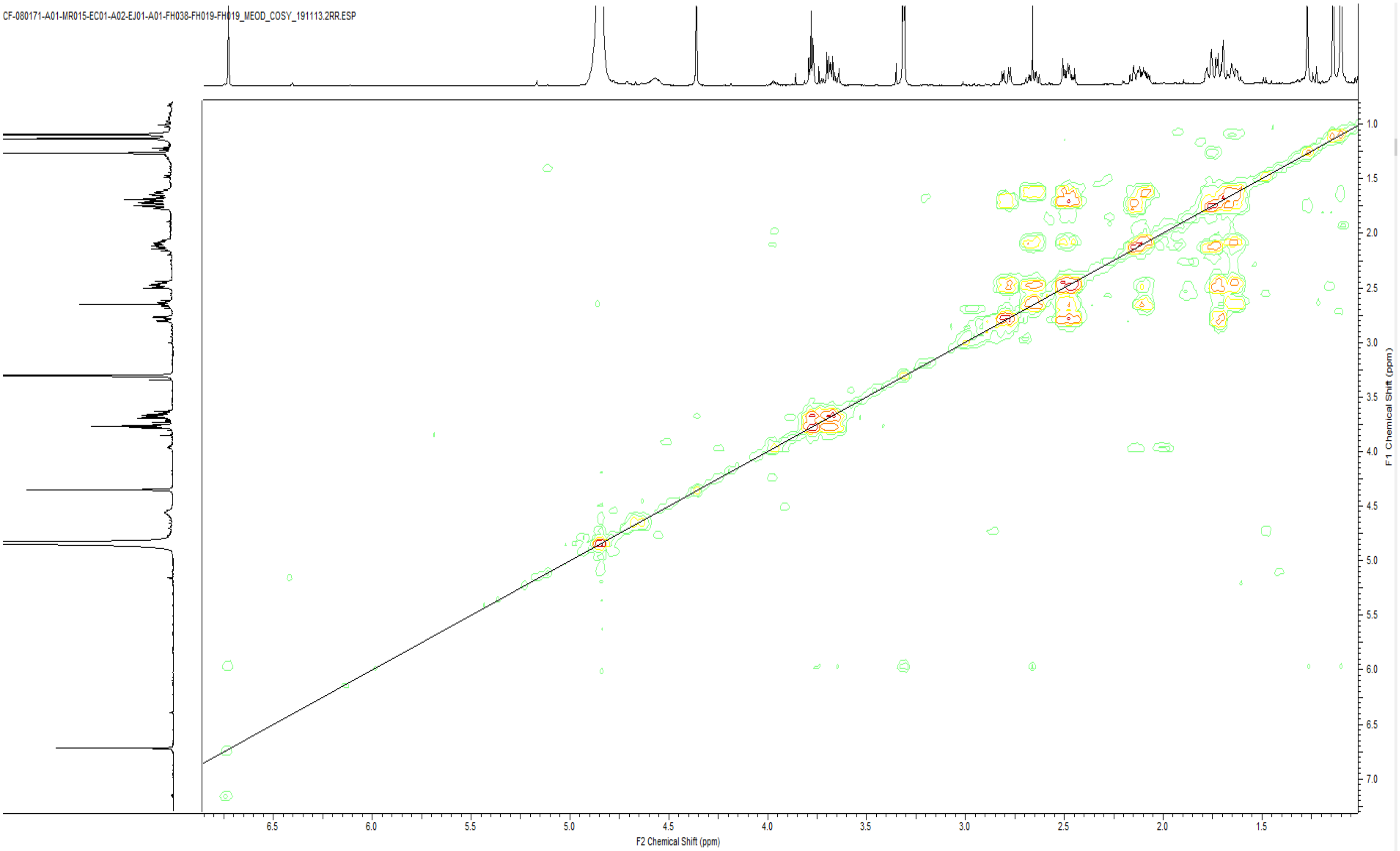

Figure S8. COSY (CD_3OD) spectrum of **1**.

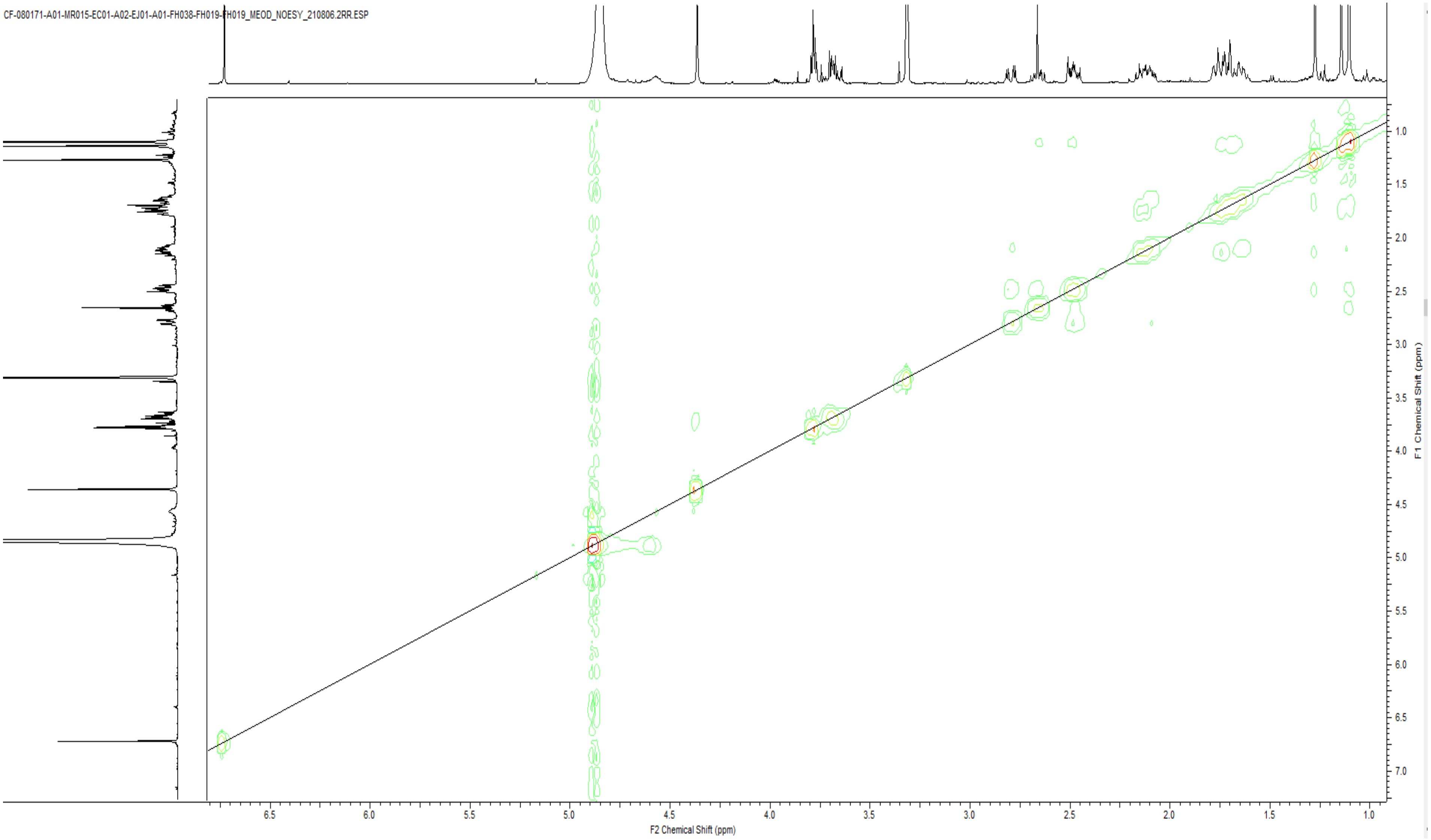

Figure S9. NOESY (CD_3OD) spectrum of **1**.

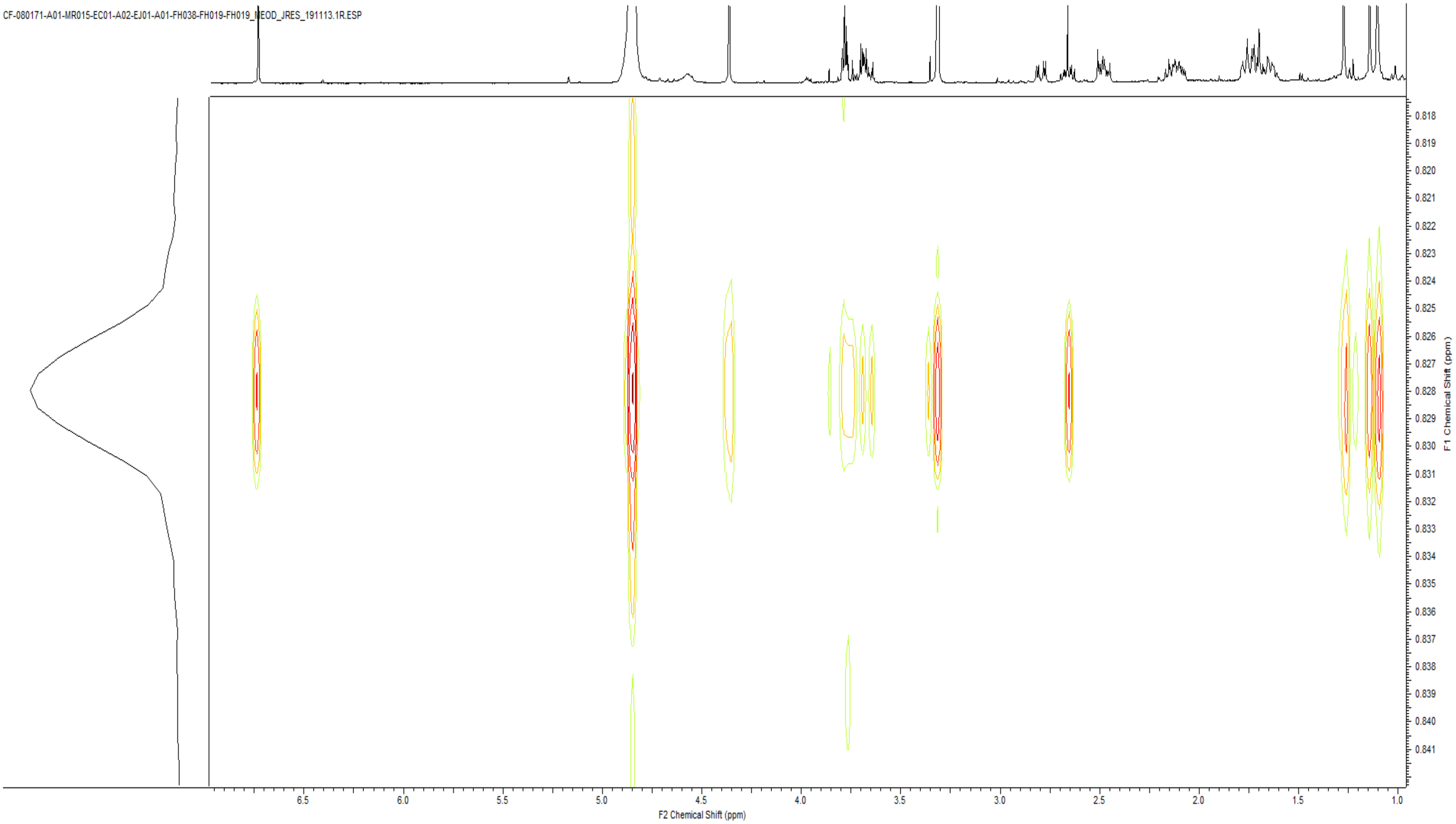

Figure S10. JRES (CD_3OD) spectrum of **1**.

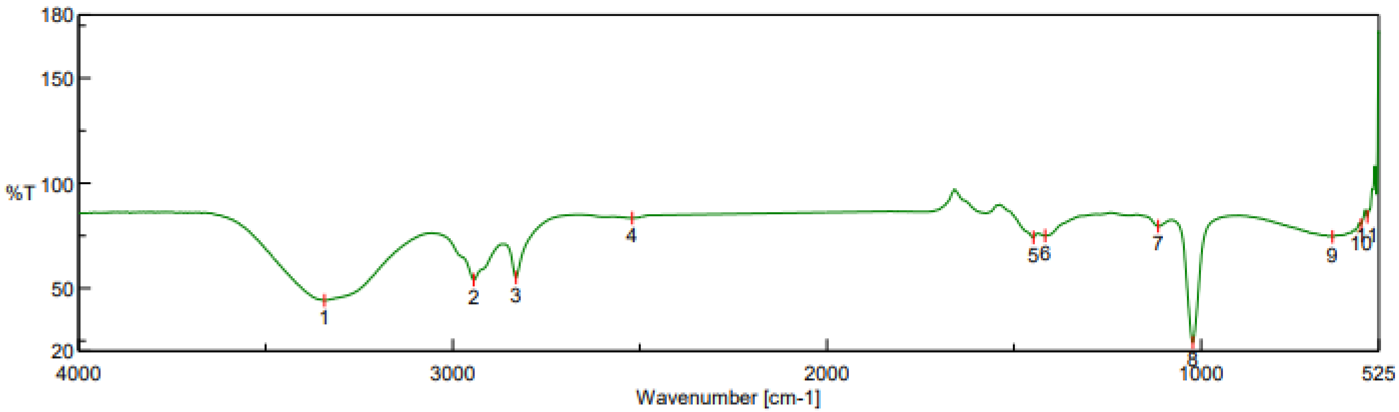

Figure S11. IR (MeOH) spectrum of **1**.

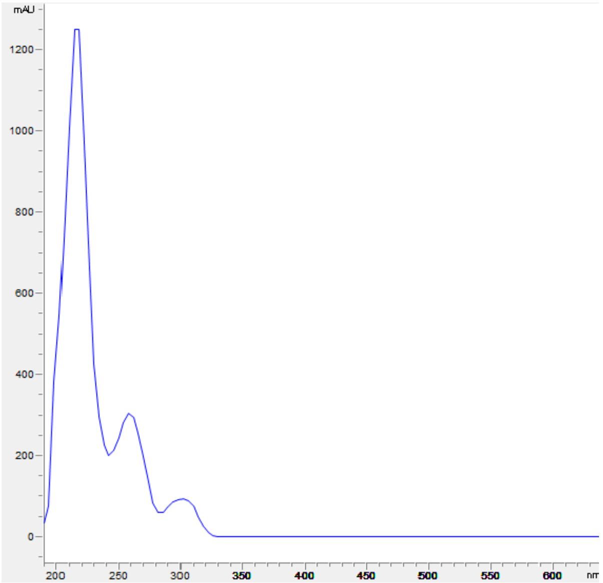

Figure S12. UV/vis (DAD) spectrum **2**.

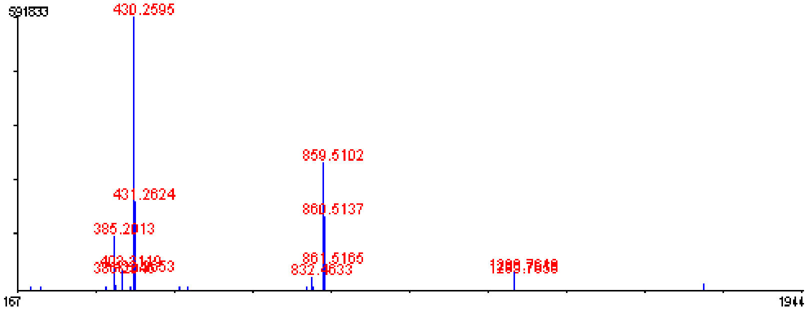

Calc. M+H$^+$= 430.2588

Calc. 2M+H$^+$= 859.5104

Figure S13. ESI-TOF spectra of **2**.

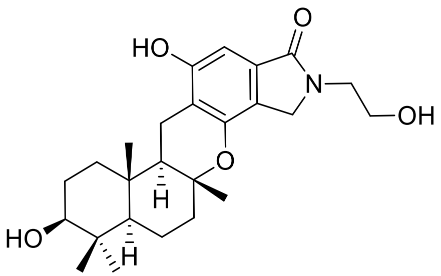

2

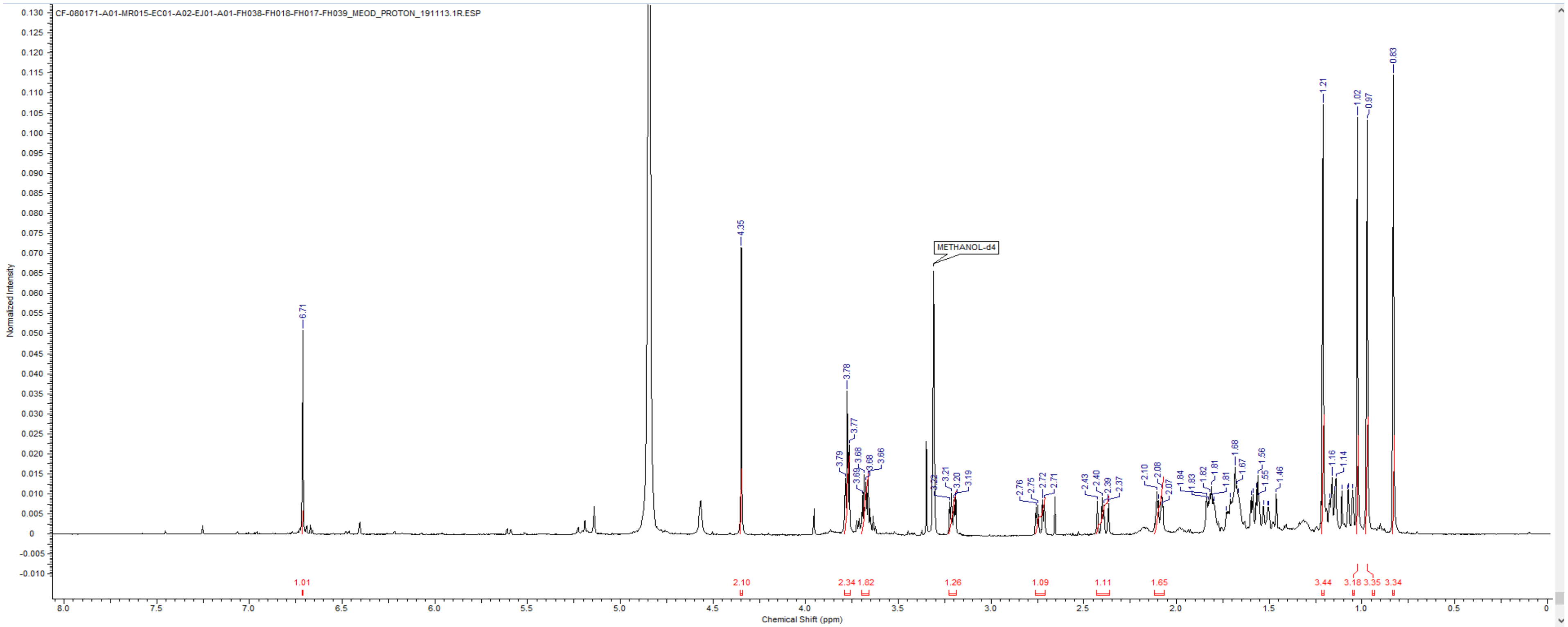

Figure S14. ^{1}H-NMR (500 MHz, CD_3OD) spectrum of **2**.

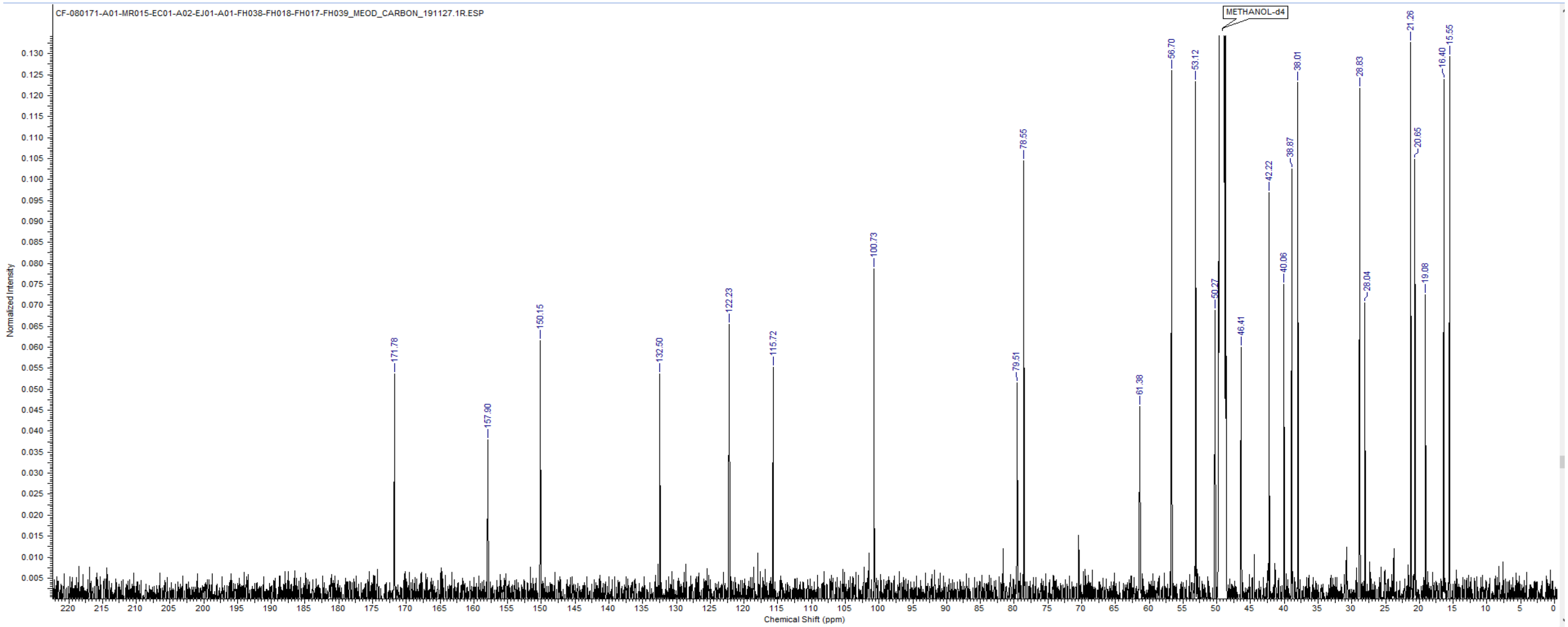

Figure S15. ^{13}C-NMR (125 MHz, CD_3OD) spectrum of **2**.

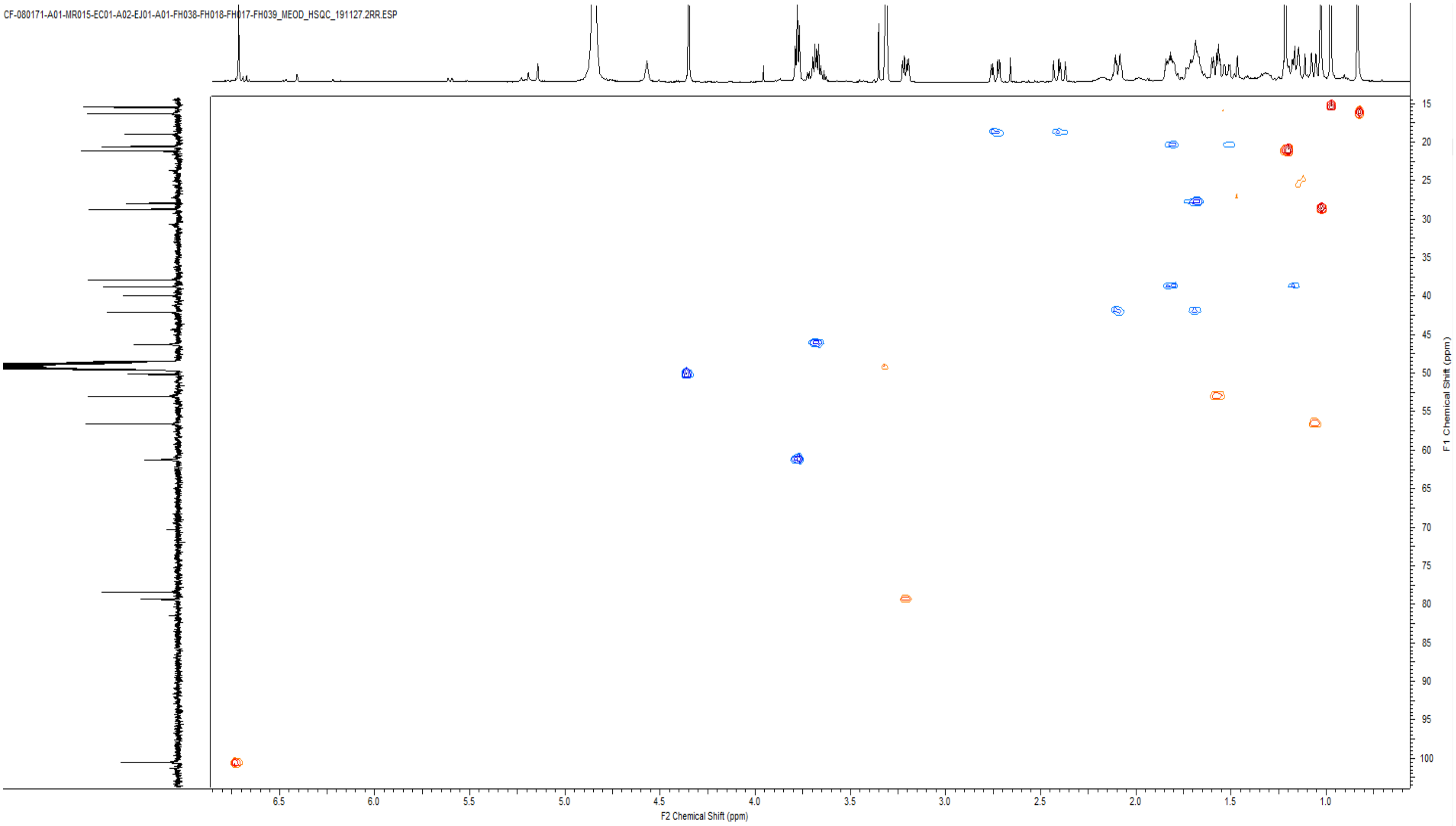

Figure S16. HSQC (CD_3OD) spectrum of **2**.

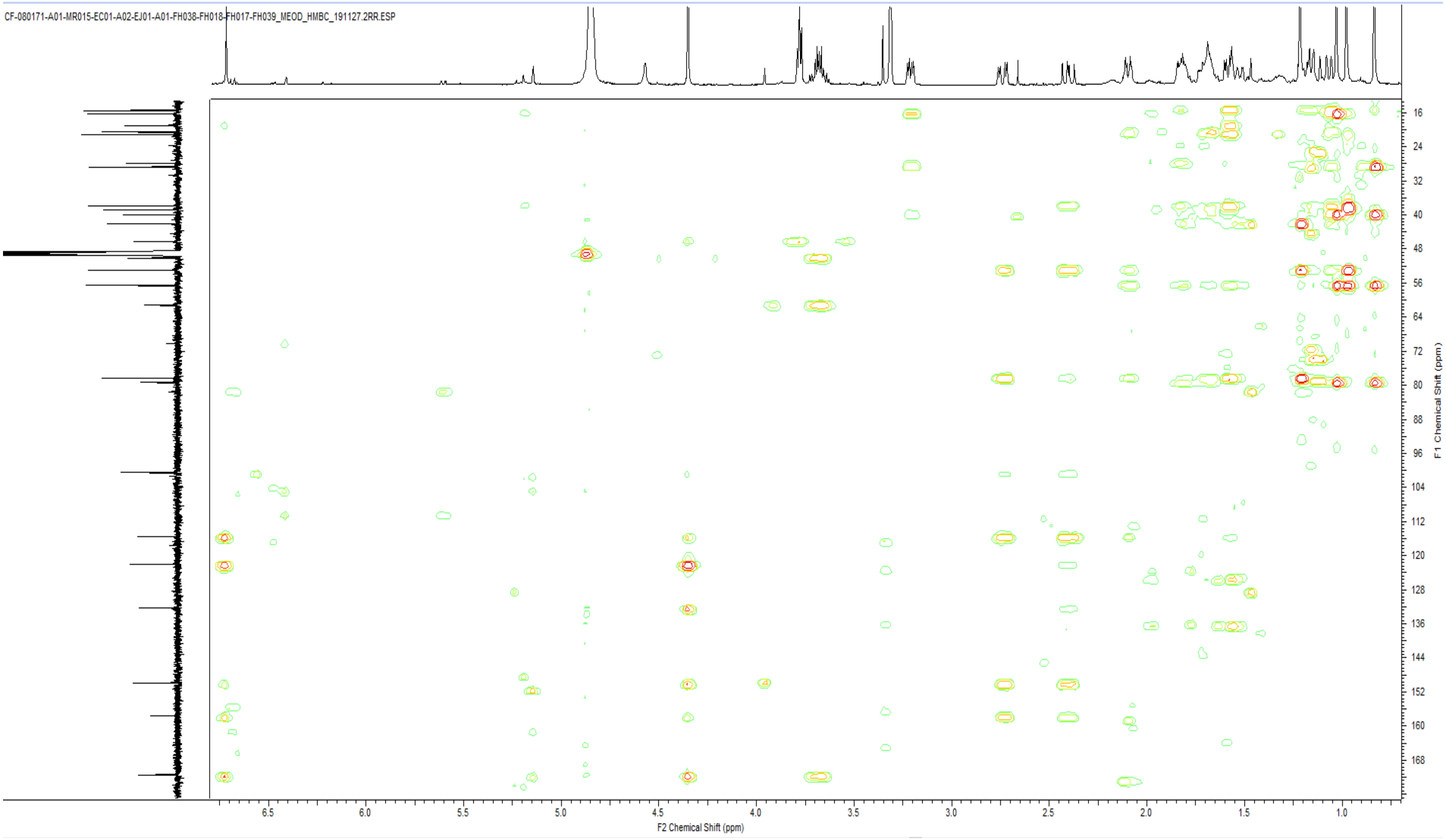

Figure S17. HMBC (CD_3OD) spectrum of **2**.

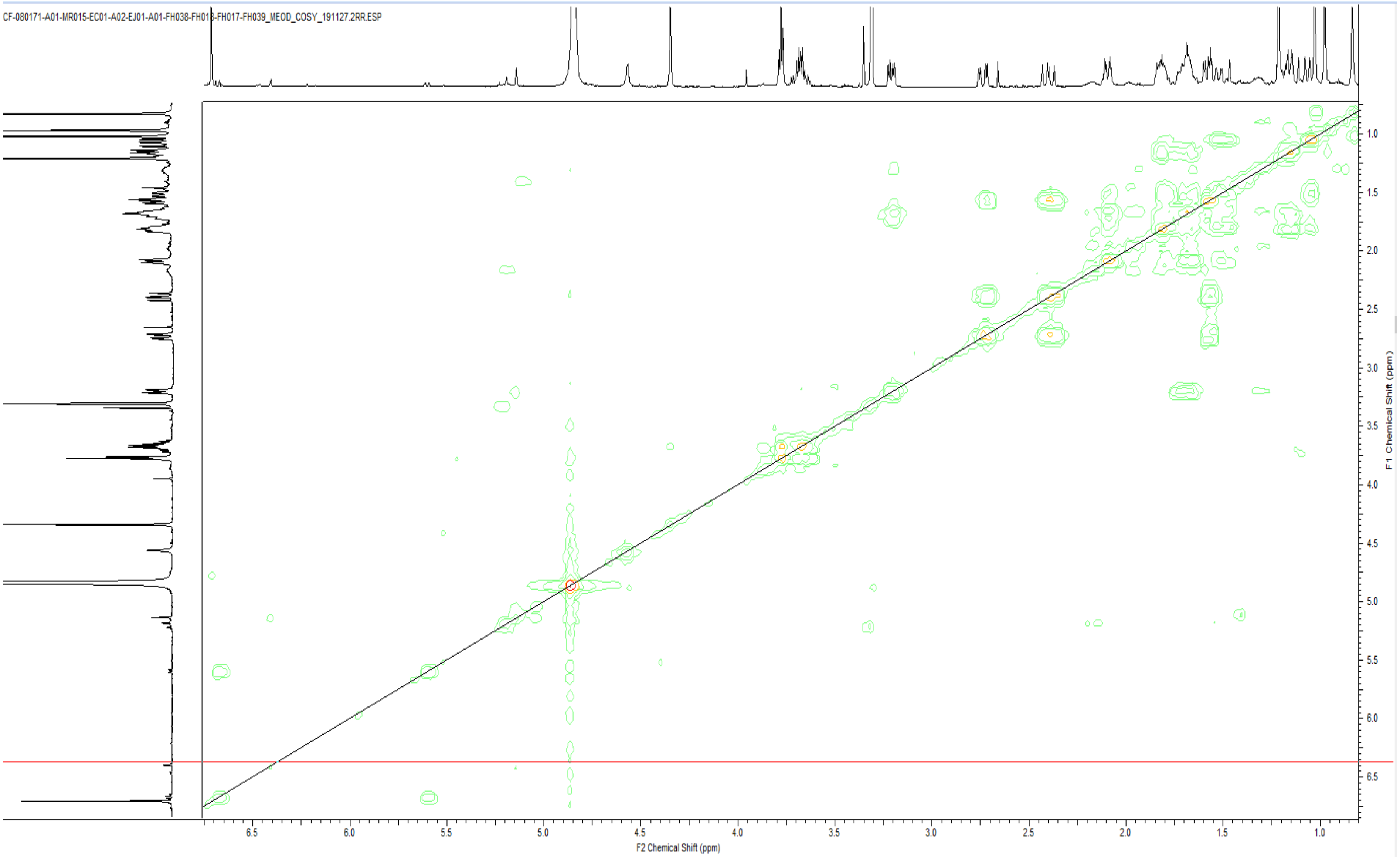

Figure S18. COSY (CD_3OD) spectrum of **2**.

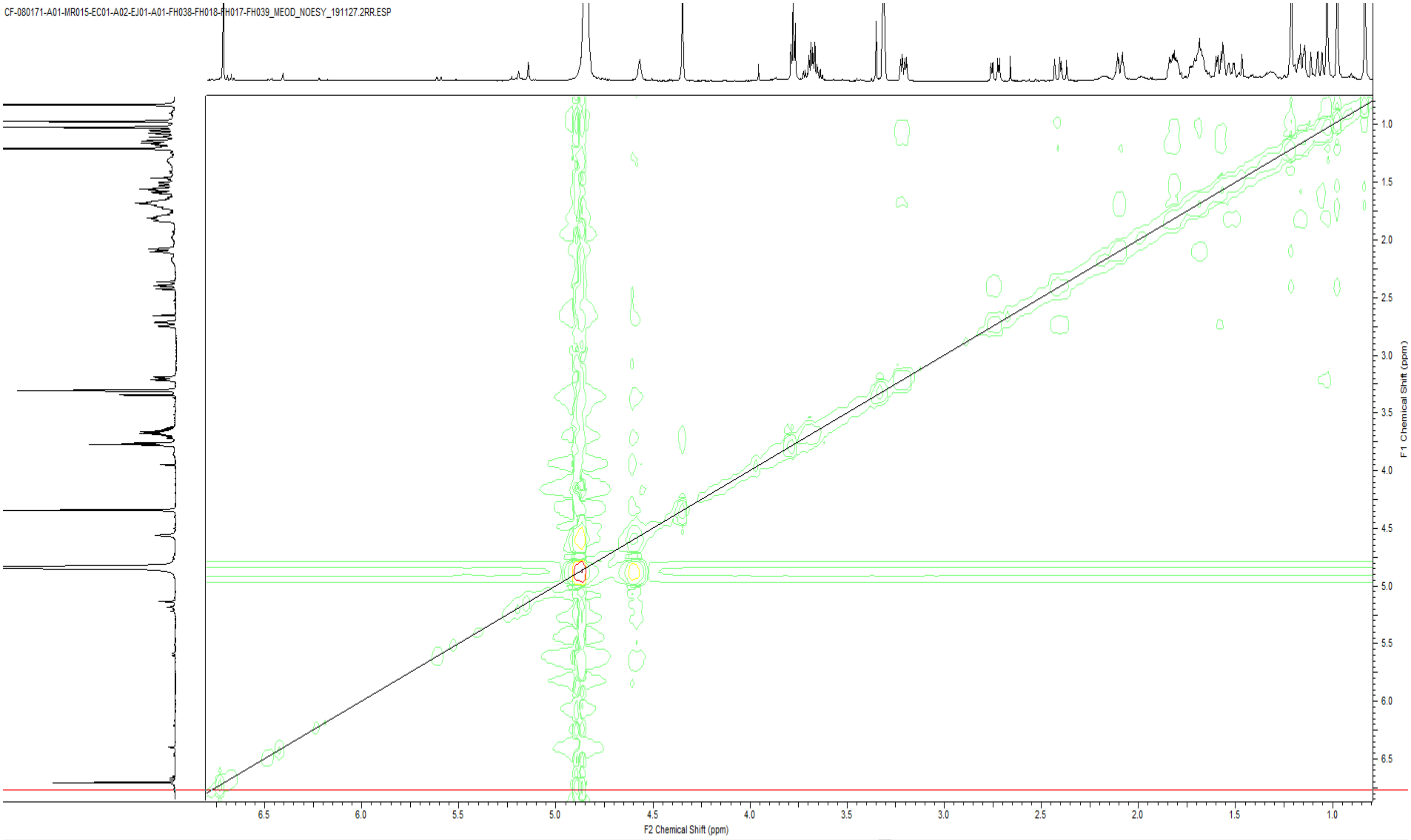

Figure S19. NOESY (CD_3OD) spectrum of **2**.

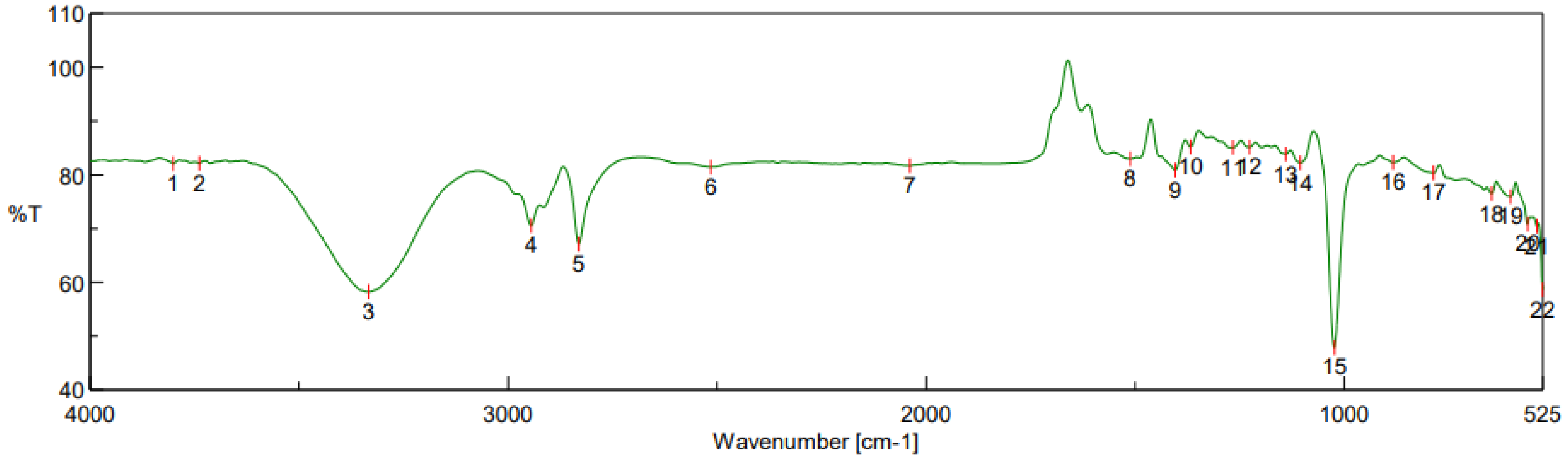

Figure S20. IR (MeOH) spectrum of **2**.

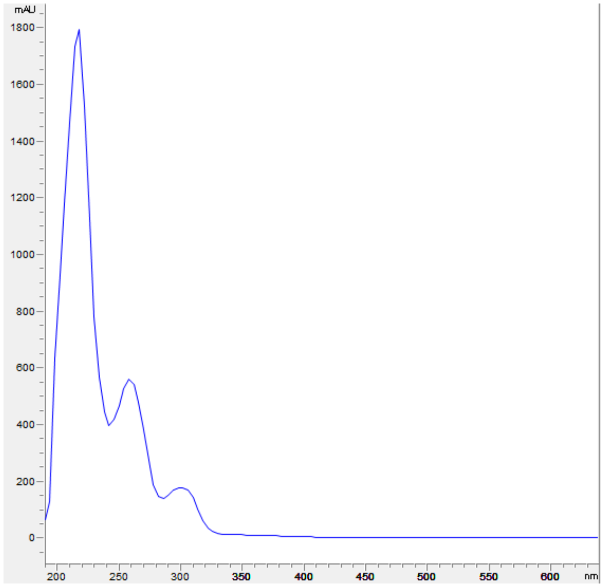

Figure S21. UV/vis (DAD) spectrum **3**.

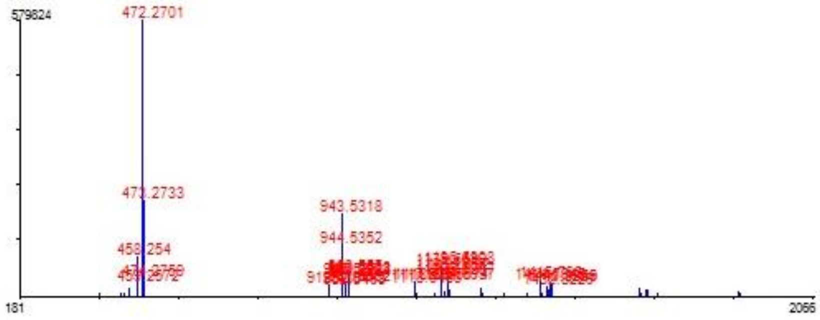

Calc. M+H$^+$= 472.2694

Calc. 2M+H$^+$= 943.5315

Figure S22. ESI-TOF spectra of **3**.

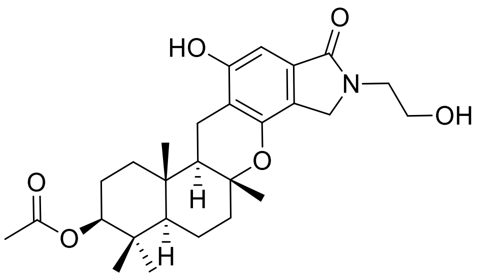

3

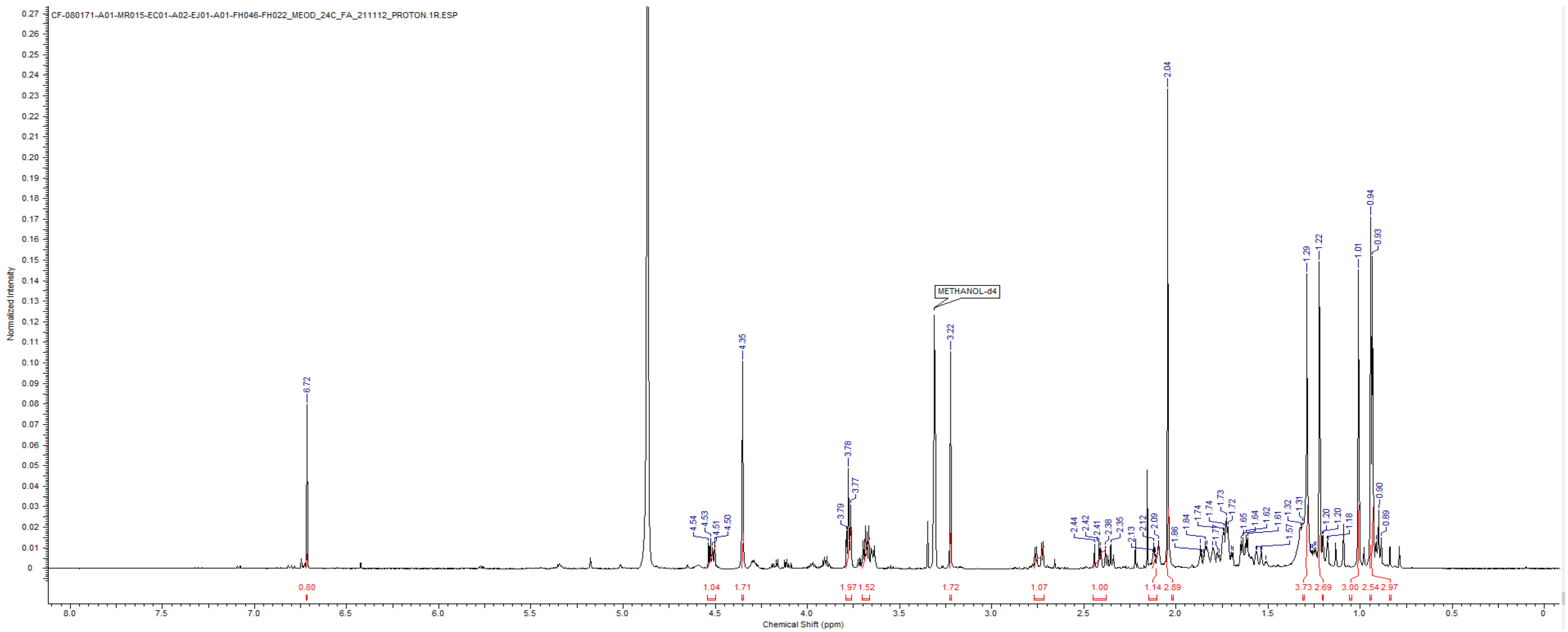

Figure S23. ^{1}H-NMR (500 MHz, CD_3OD) spectrum of **3**.

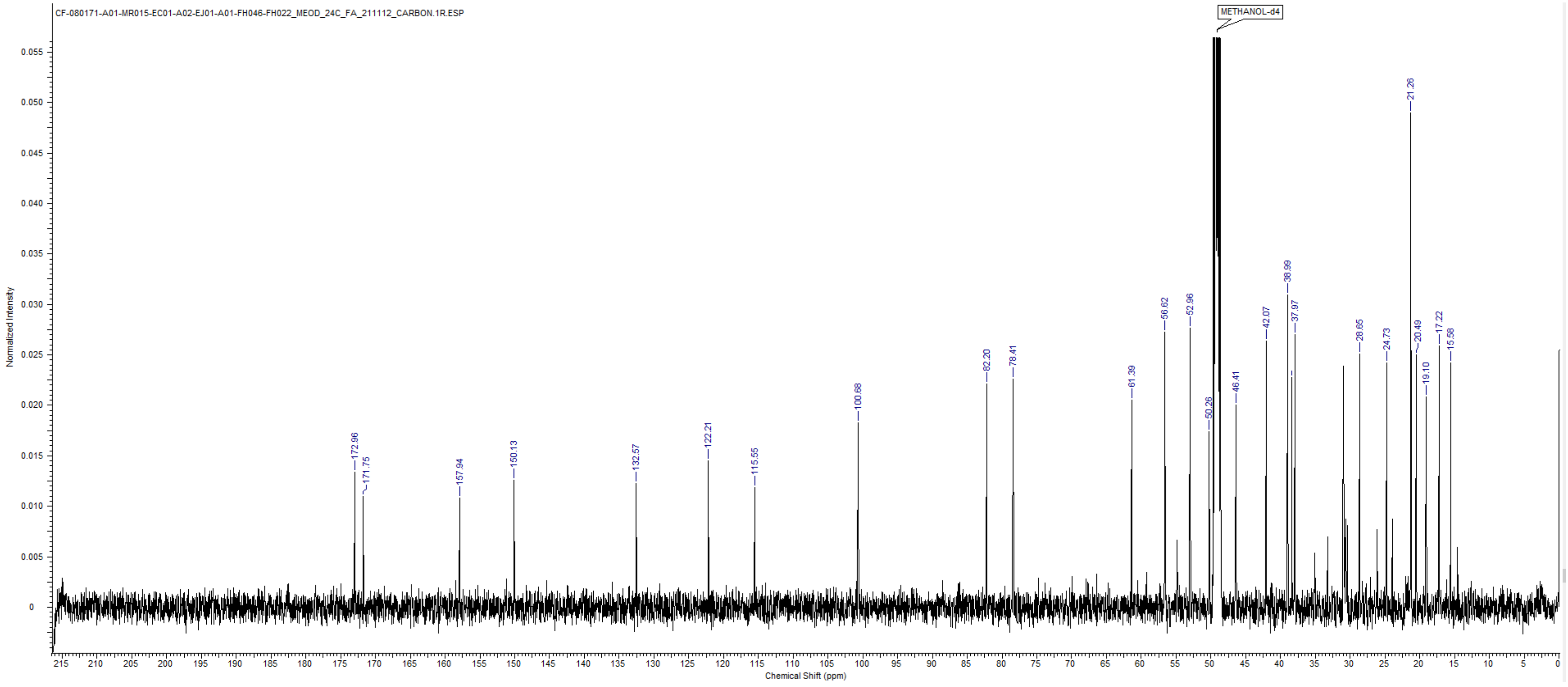

Figure S24. ^{13}C-NMR (125 MHz, CD_3OD) spectrum of **3**.

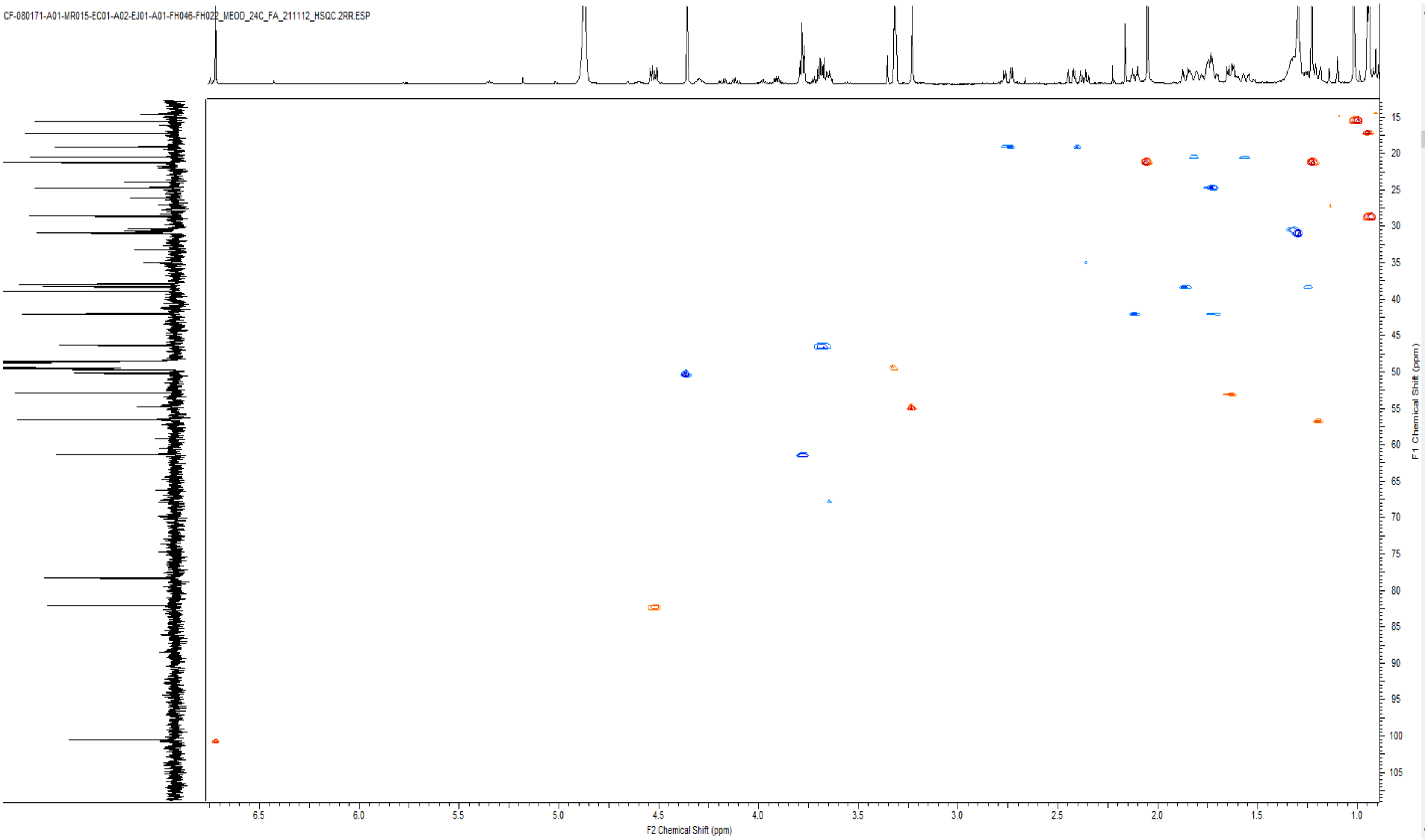

Figure S25. HSQC (CD_3OD) spectrum of **3**.

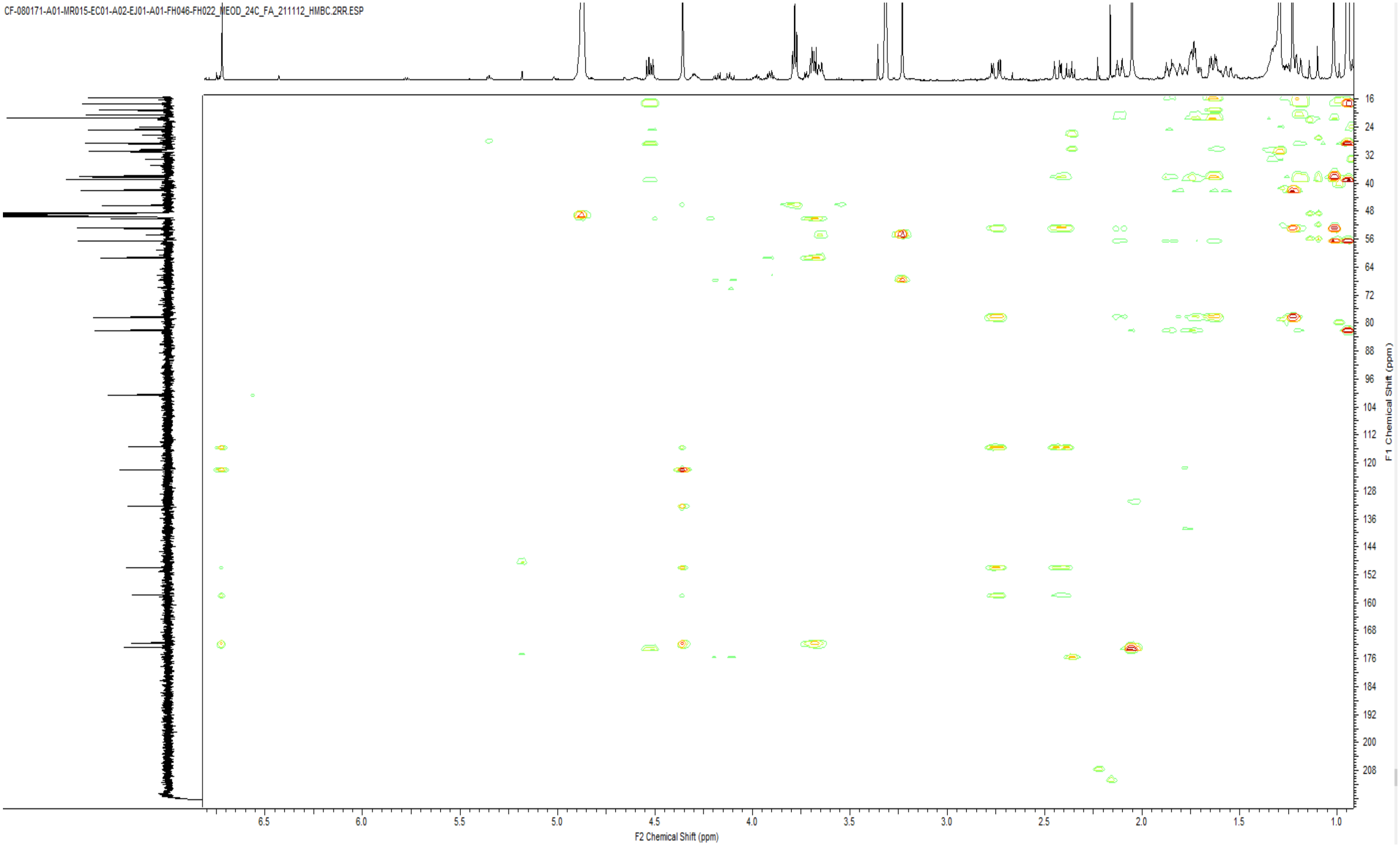

Figure S26. HMBC (CD_3OD) spectrum of **3**.

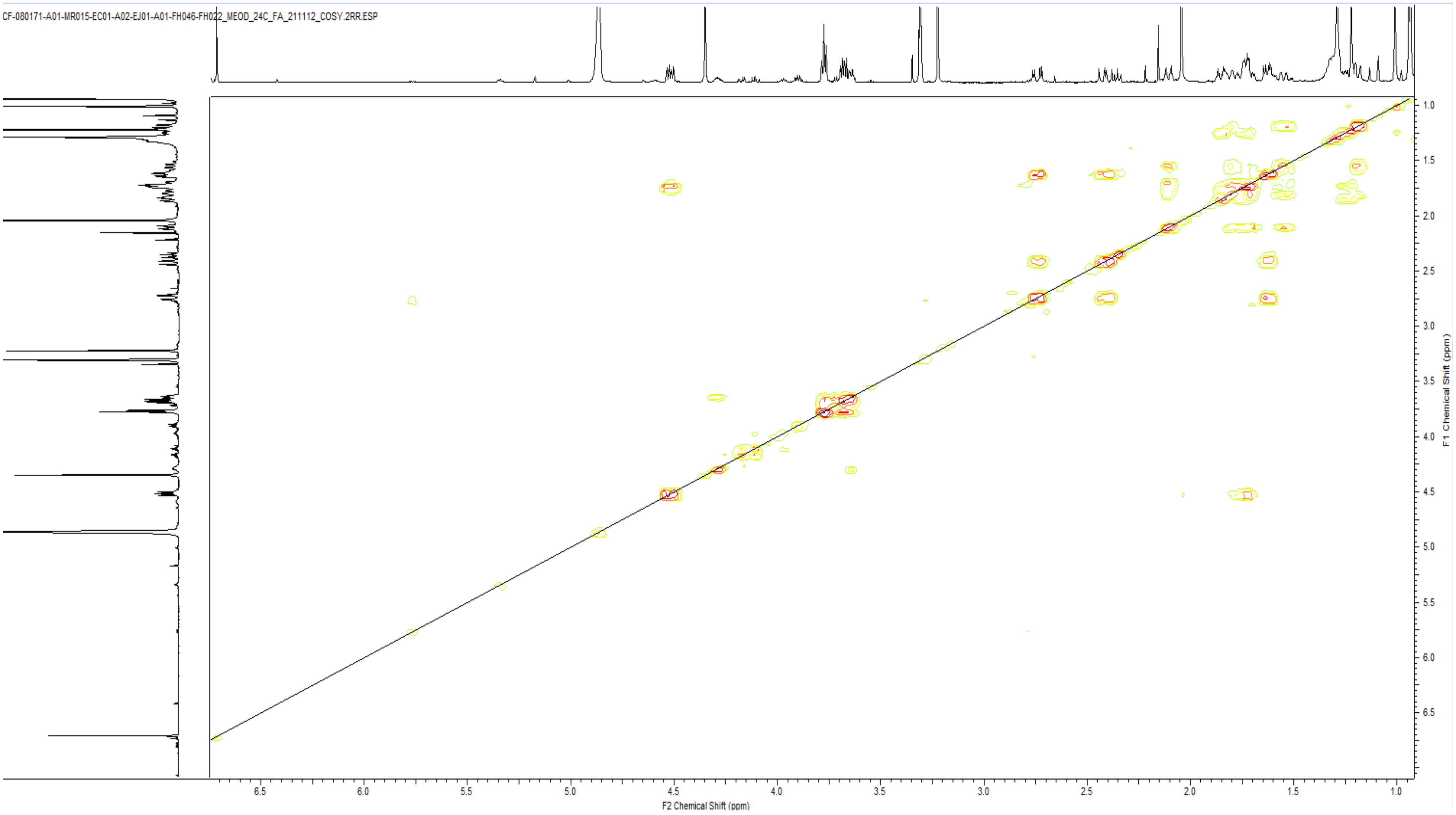

Figure S27. COSY (CD_3OD) spectrum of **3**.

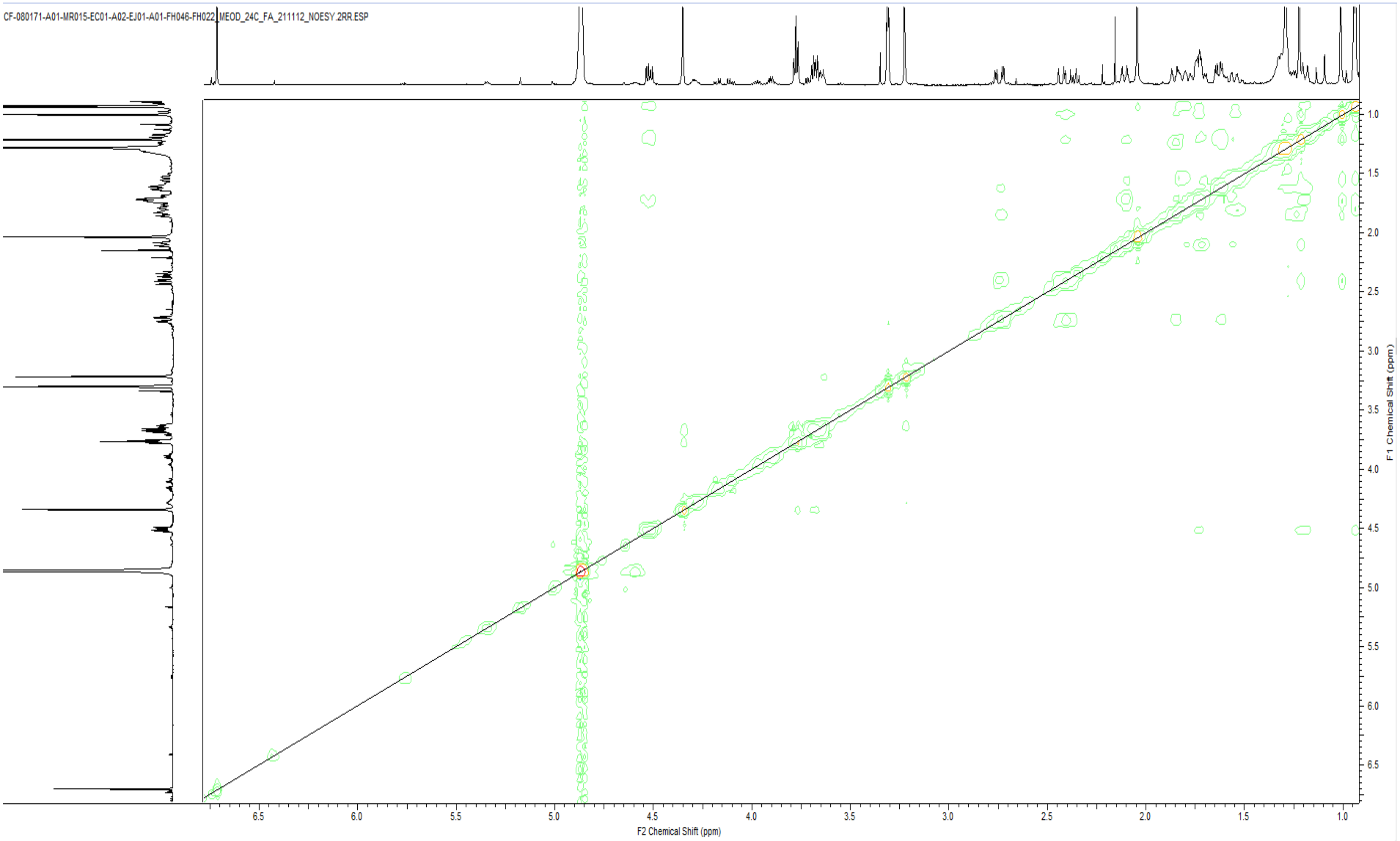

Figure S28. NOESY (CD_3OD) spectrum of **3**.

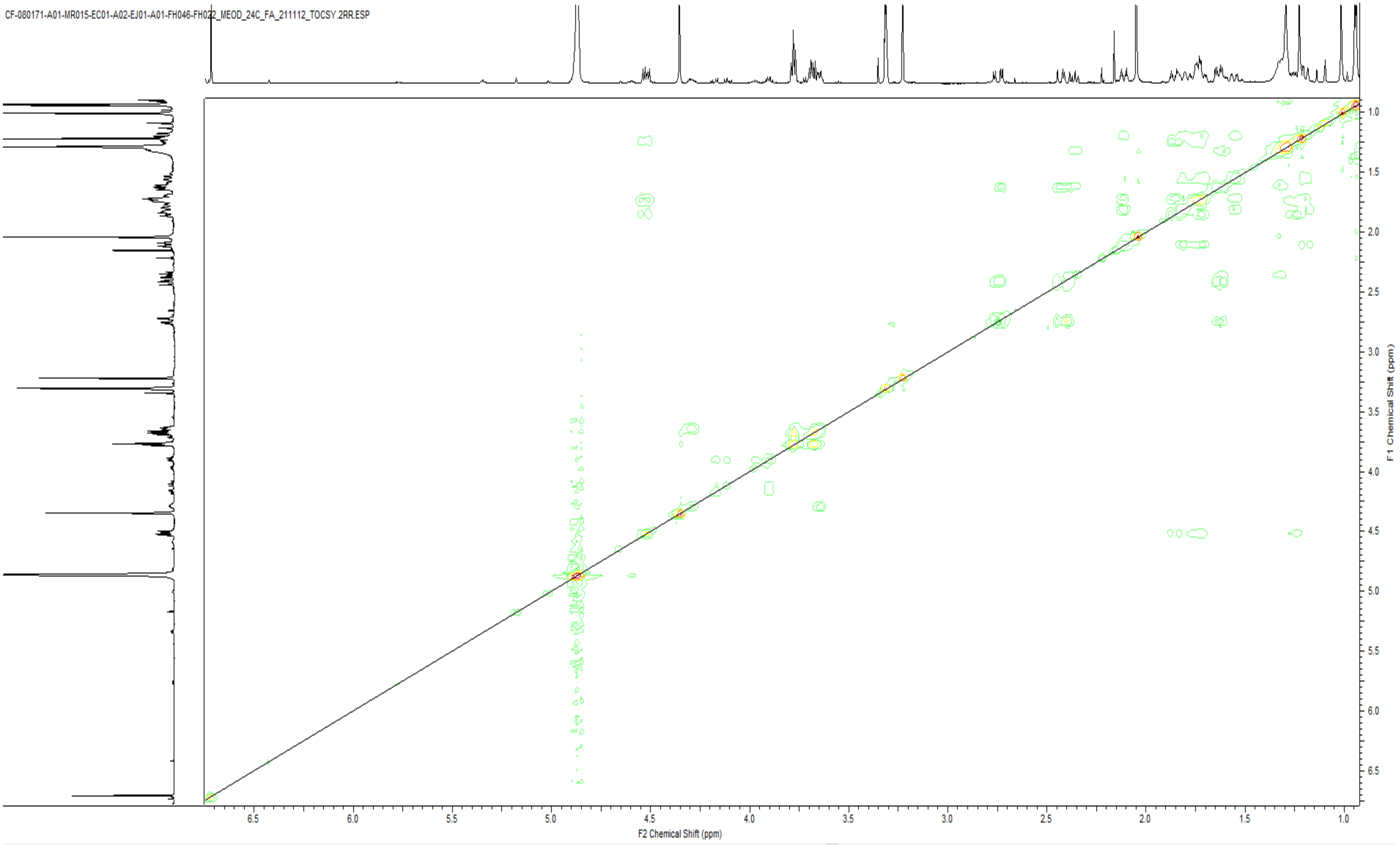

Figure S29. TOCSY (CD_3OD) spectrum of **3**.

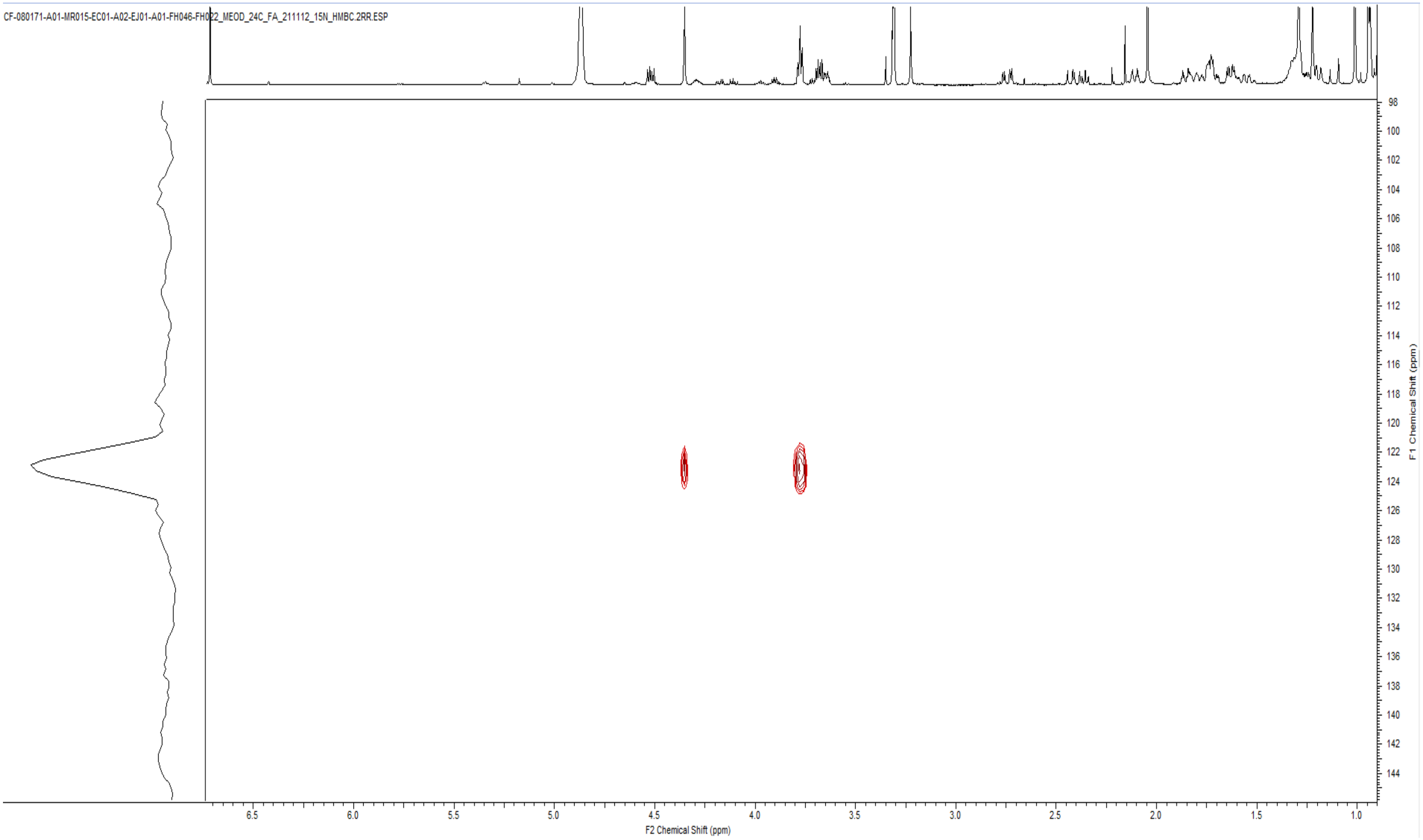

Figure S30. ^{15}N-HMBC (CD_3OD) spectrum of **3**.

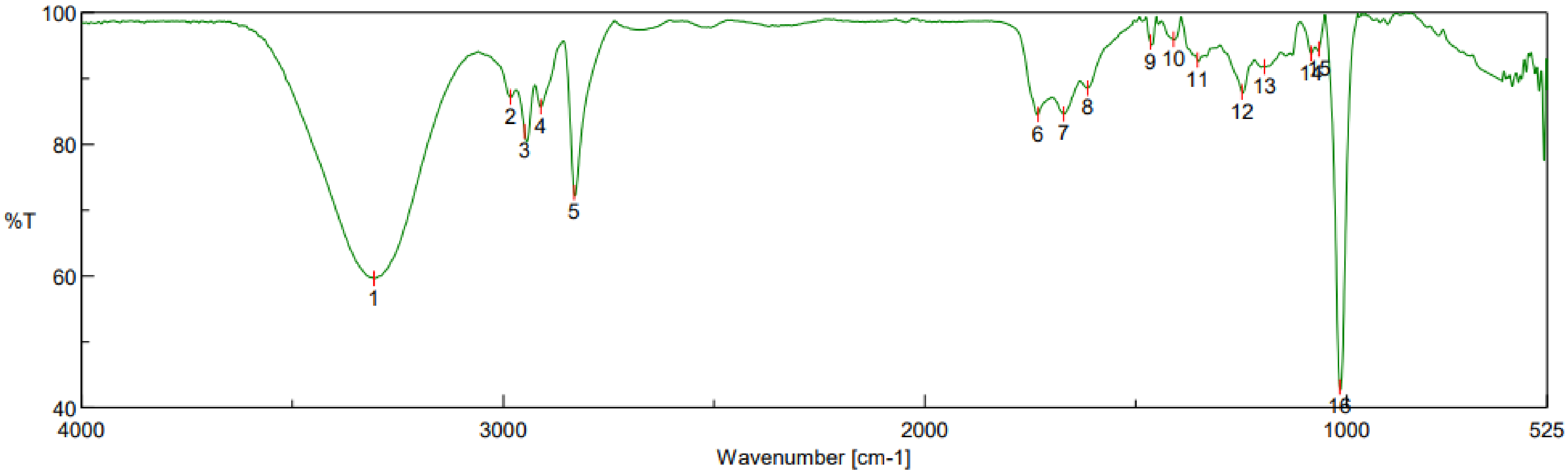

Figure S31. IR (MeOH) spectrum of **3**.

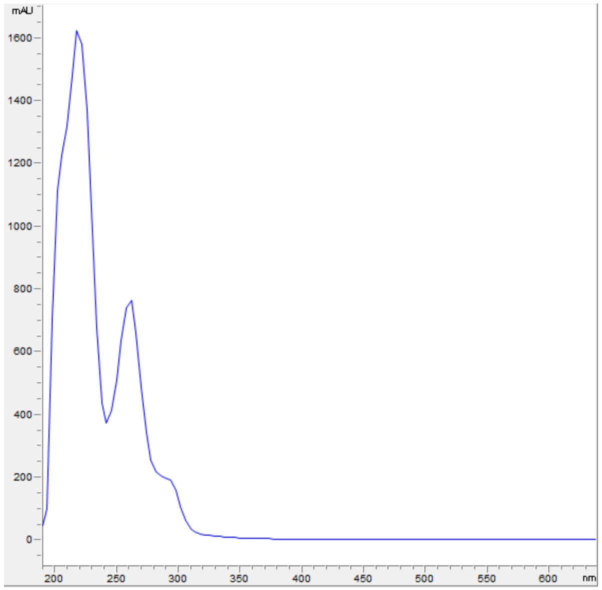

Figure S32. UV/vis (DAD) spectrum **4**.

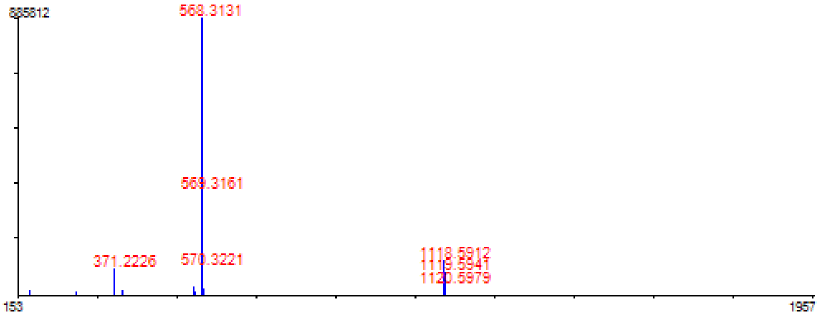

Calc. M+NH_4^+= 568.3117

Calc. M-$C_6H_{12}O_6$ +H^+= 371.2217

Figure S33. ESI-TOF spectra of **4**.

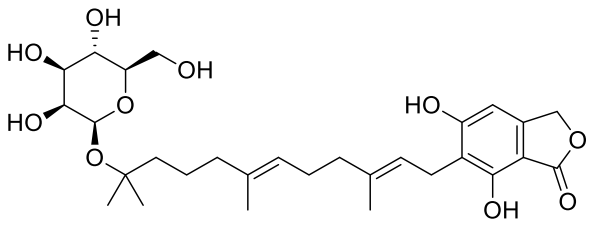

4

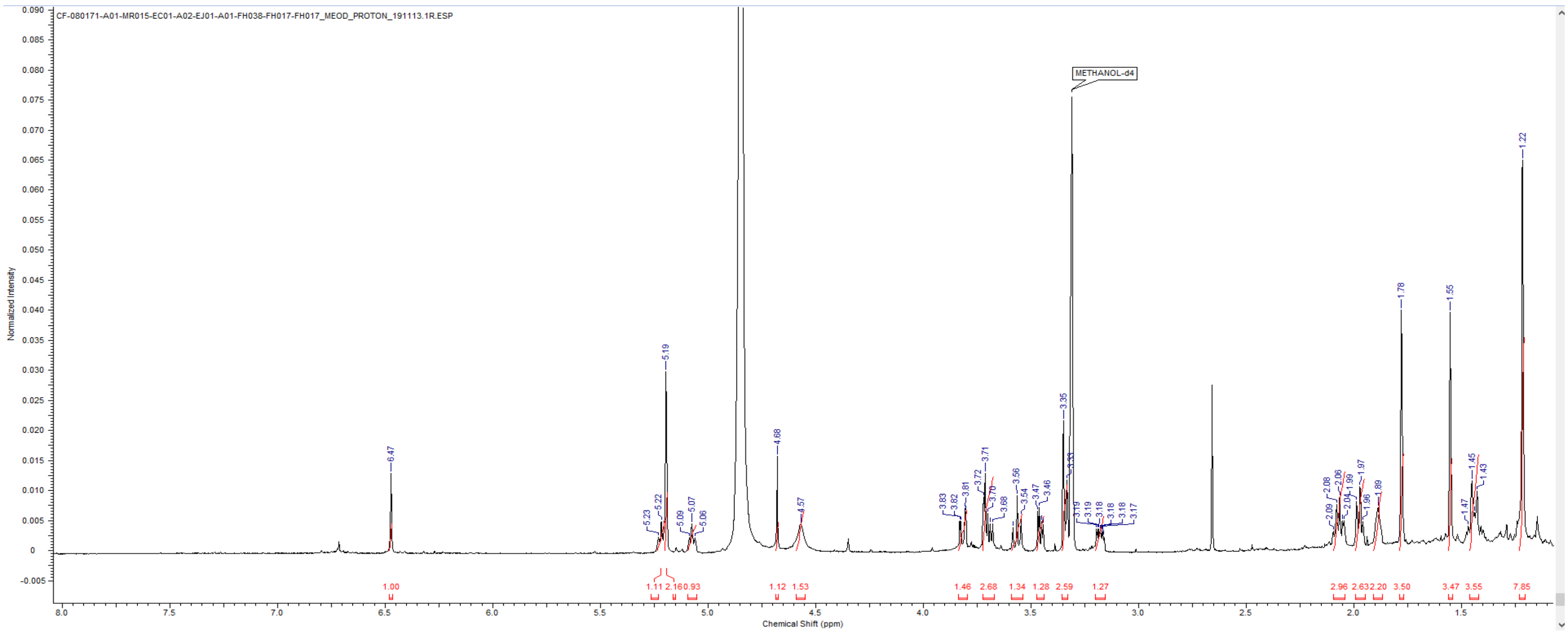

Figure S34. ^{1}H-NMR (500 MHz, CD_3OD) spectrum of **4**.

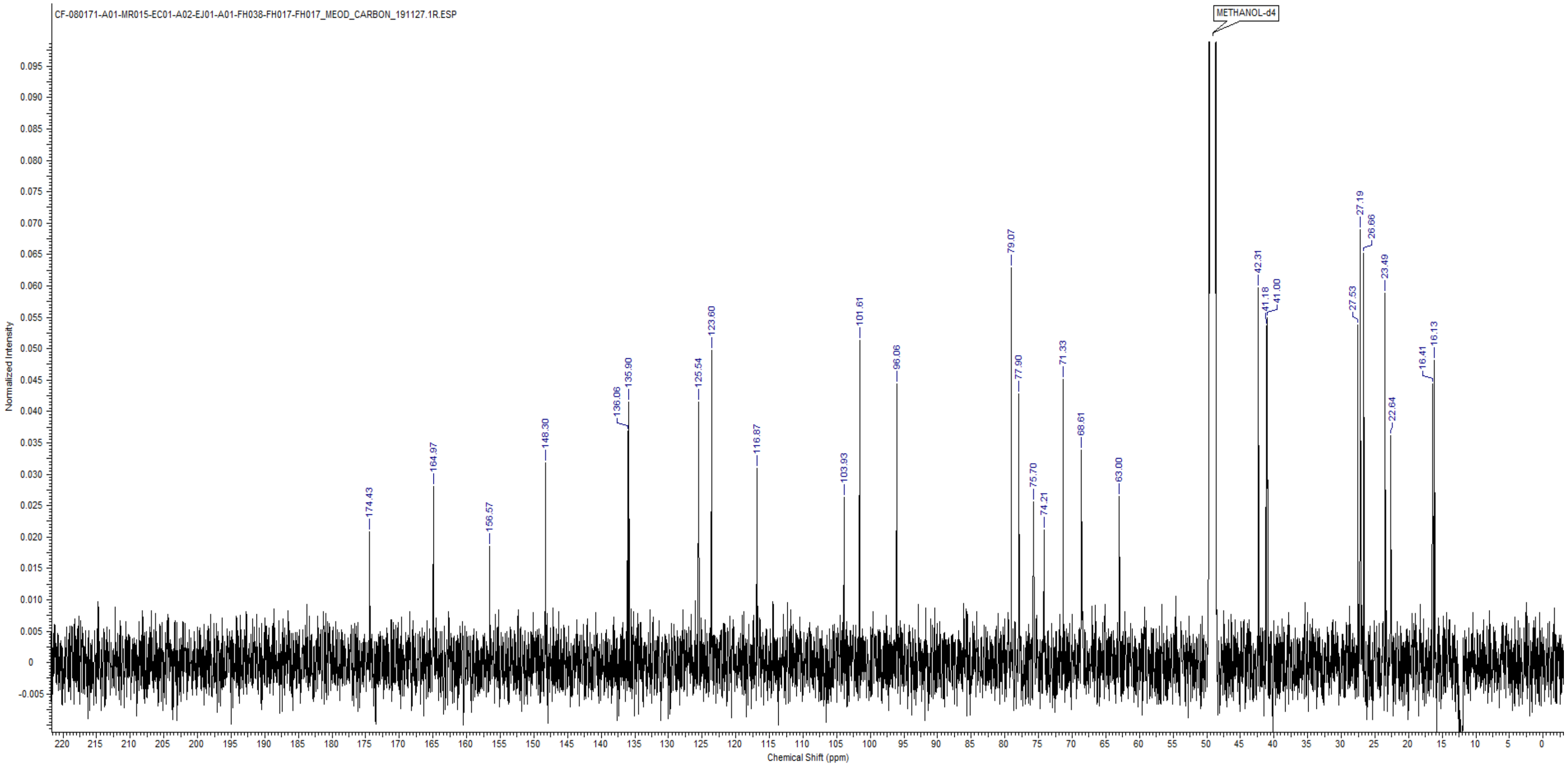

Figure S35. ^{13}C-NMR (125 MHz, CD_3OD) spectrum of **4**.

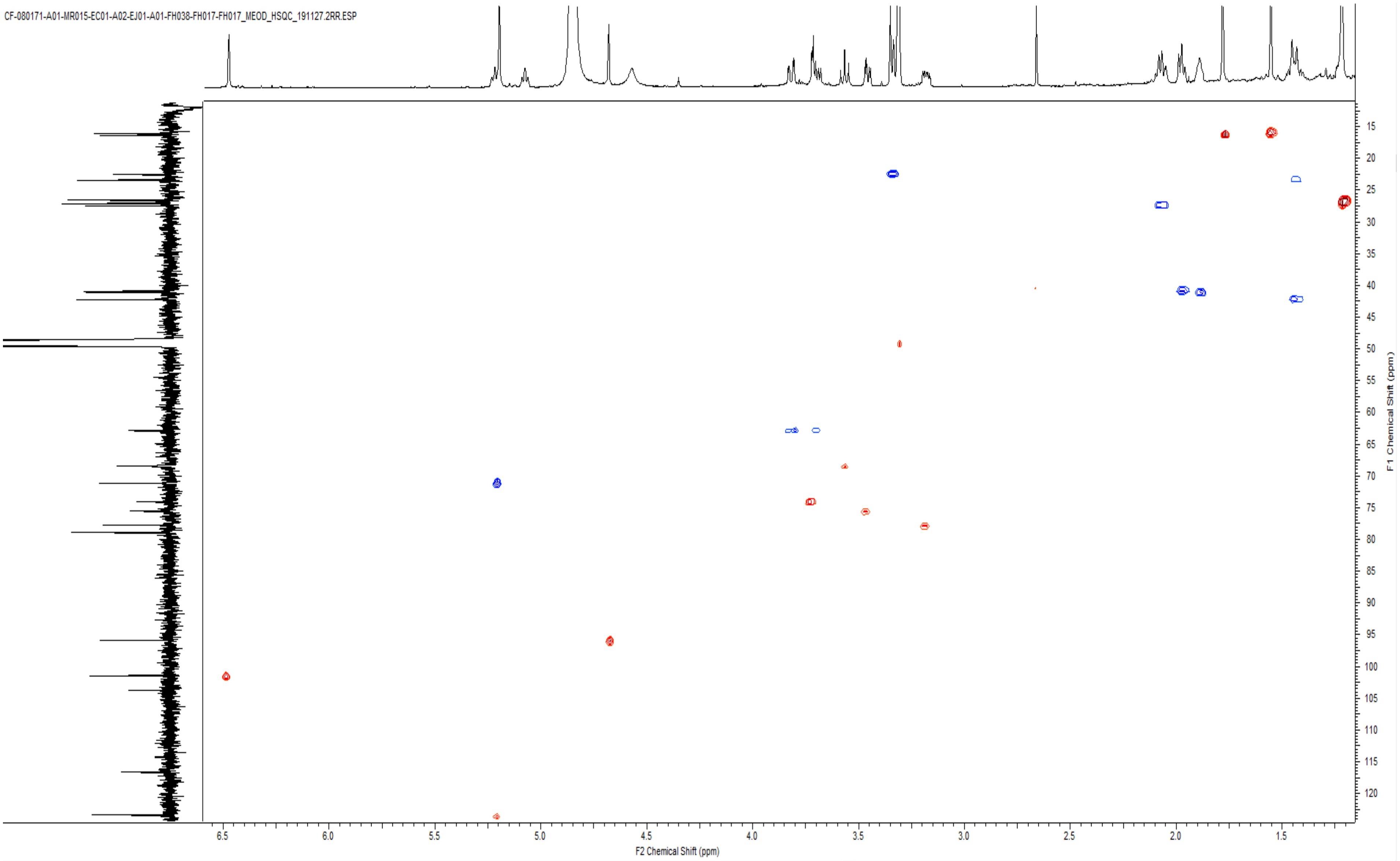

Figure S36. HSQC (CD_3OD) spectrum of **4**.

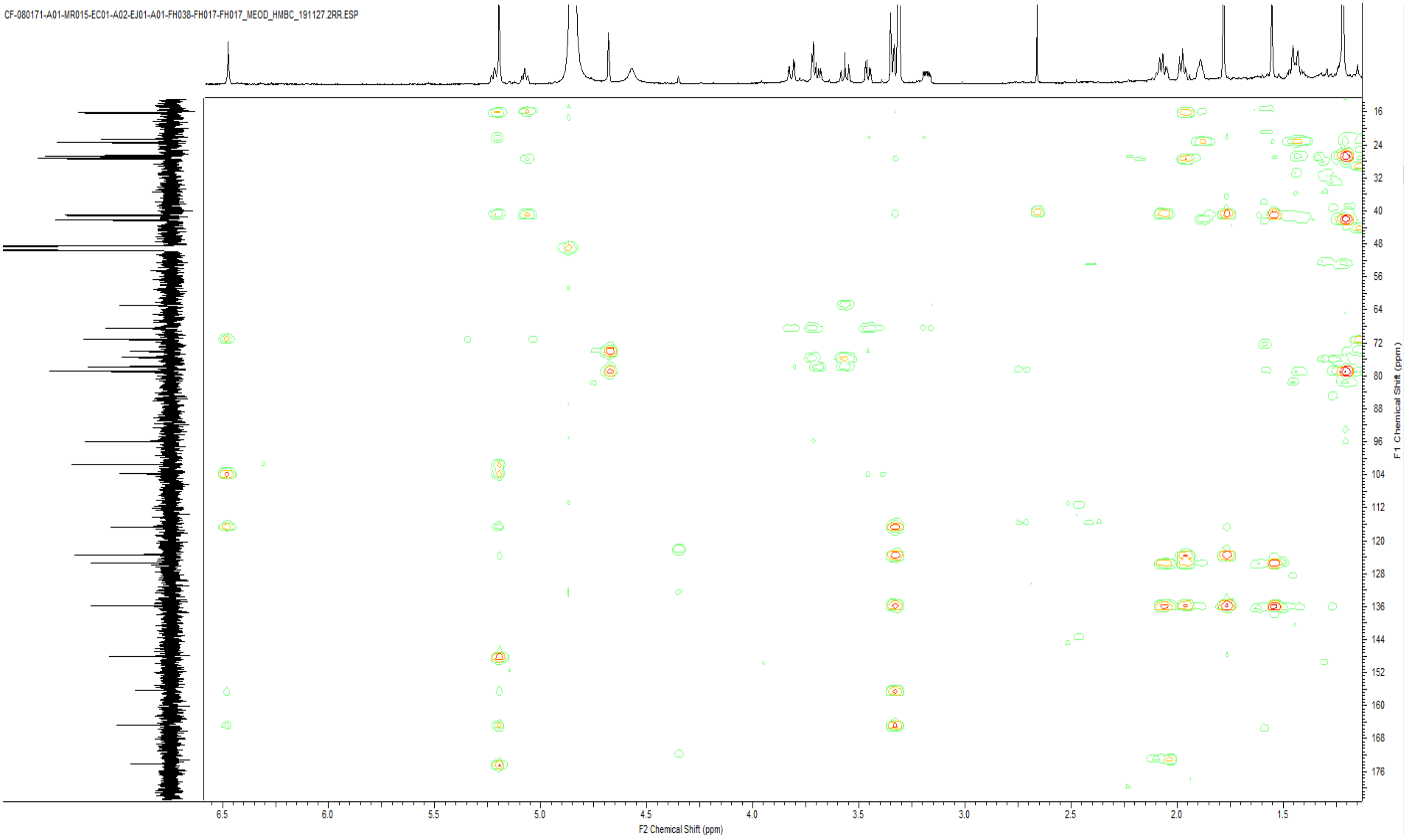

Figure S37. HMBC (CD_3OD) spectrum of **4**.

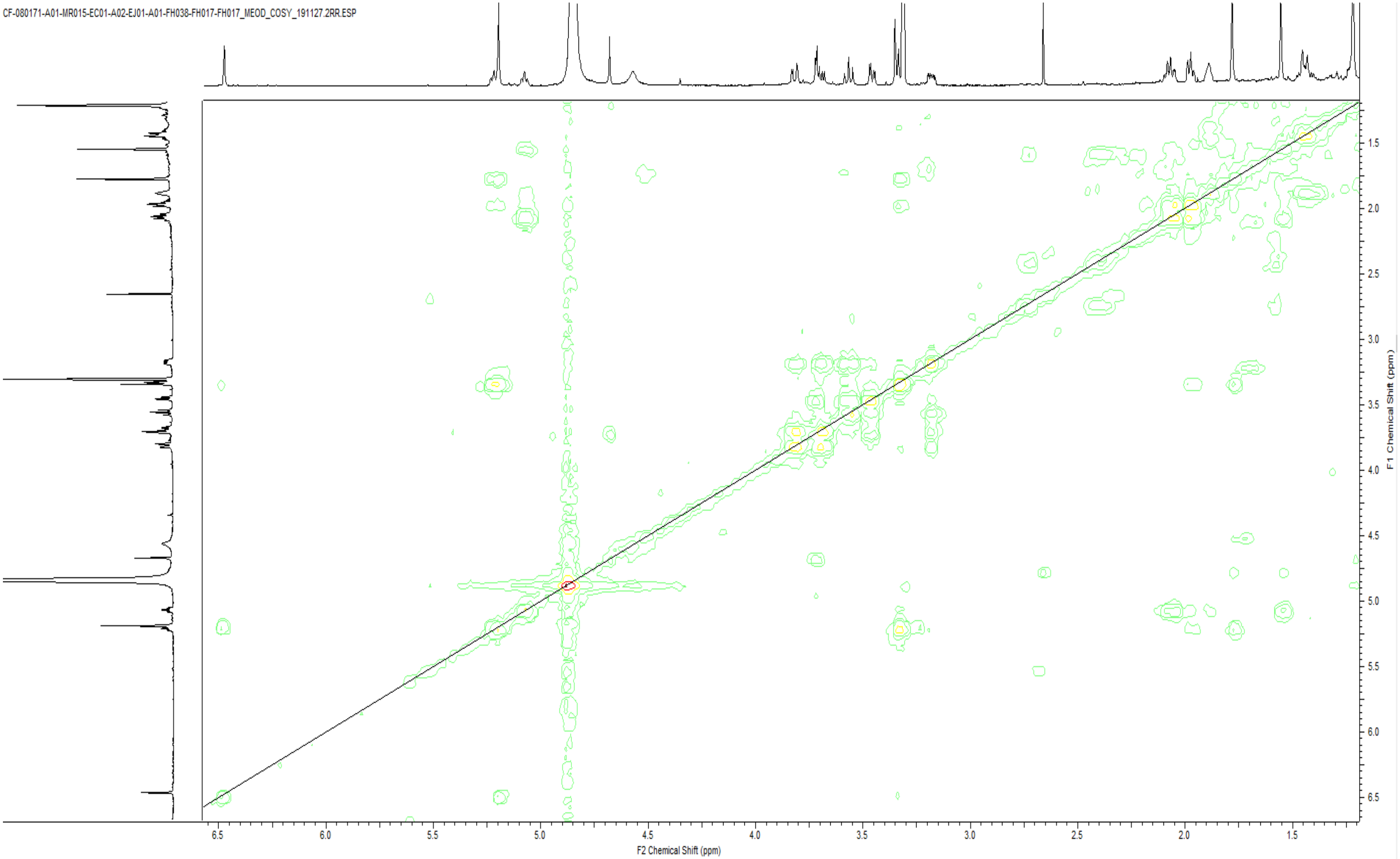

Figure S38. COSY (CD_3OD) spectrum of **4**.

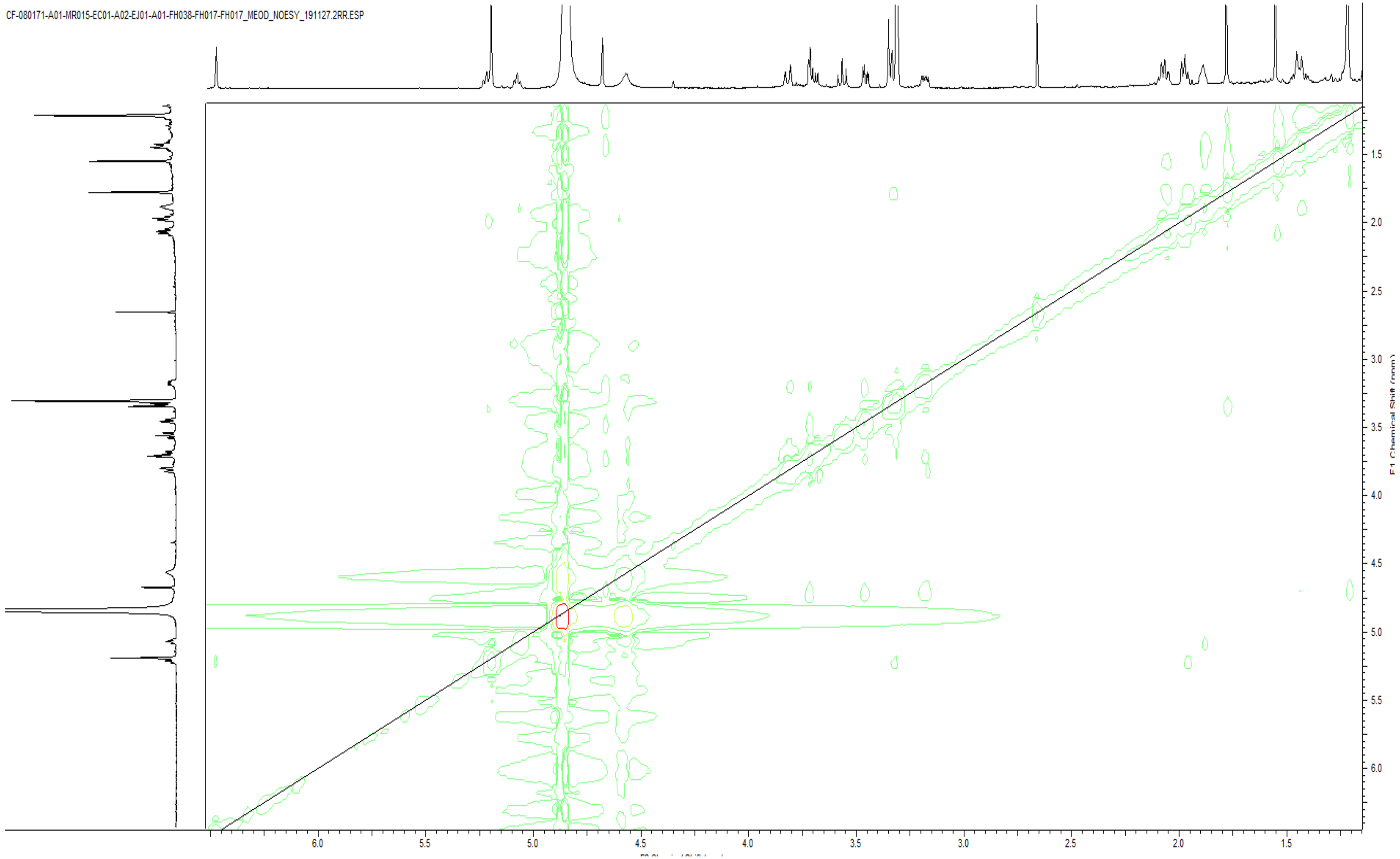

Figure S39. NOESY (CD_3OD) spectrum of **4**.

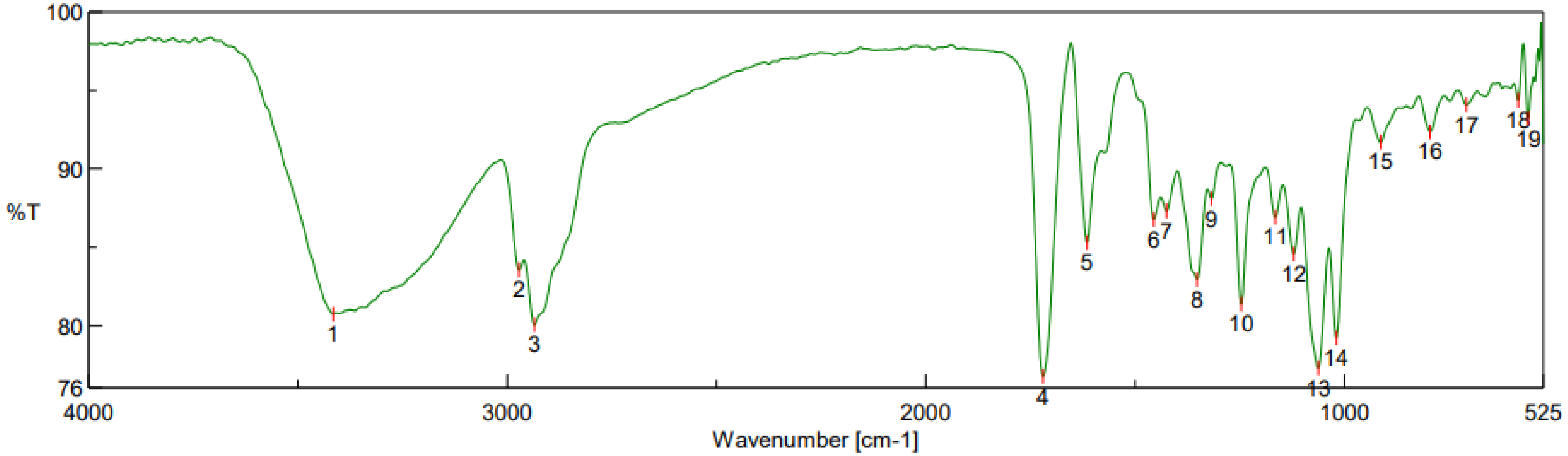

Figure S40. IR (MeOH) spectrum of **4**.

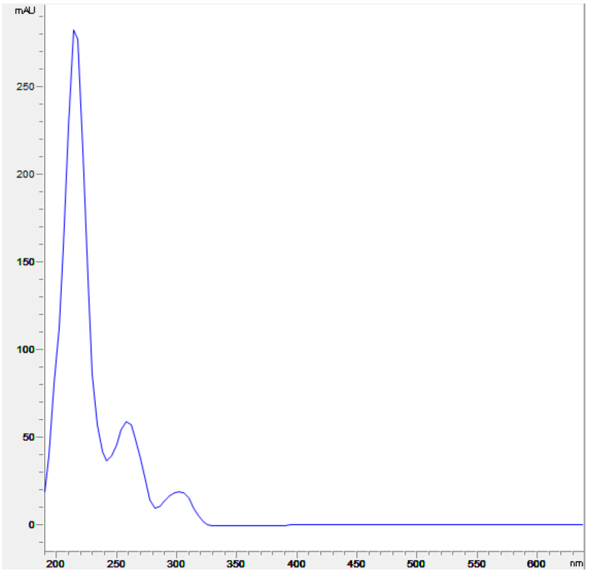

Figure S41. UV/vis (DAD) spectrum **5**.

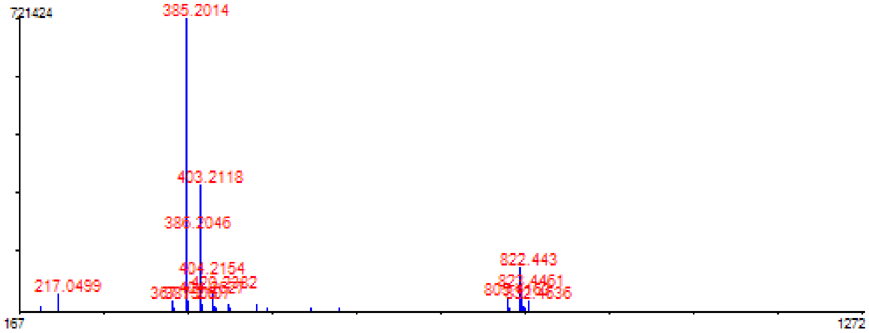

Calc. M+H$^+$= 403.2116

Calc. M-H_2O+H$^+$= 385.2010

Figure S42. ESI-TOF spectra of **5**.

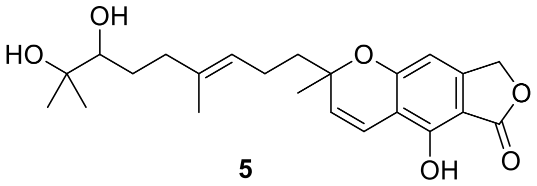

5

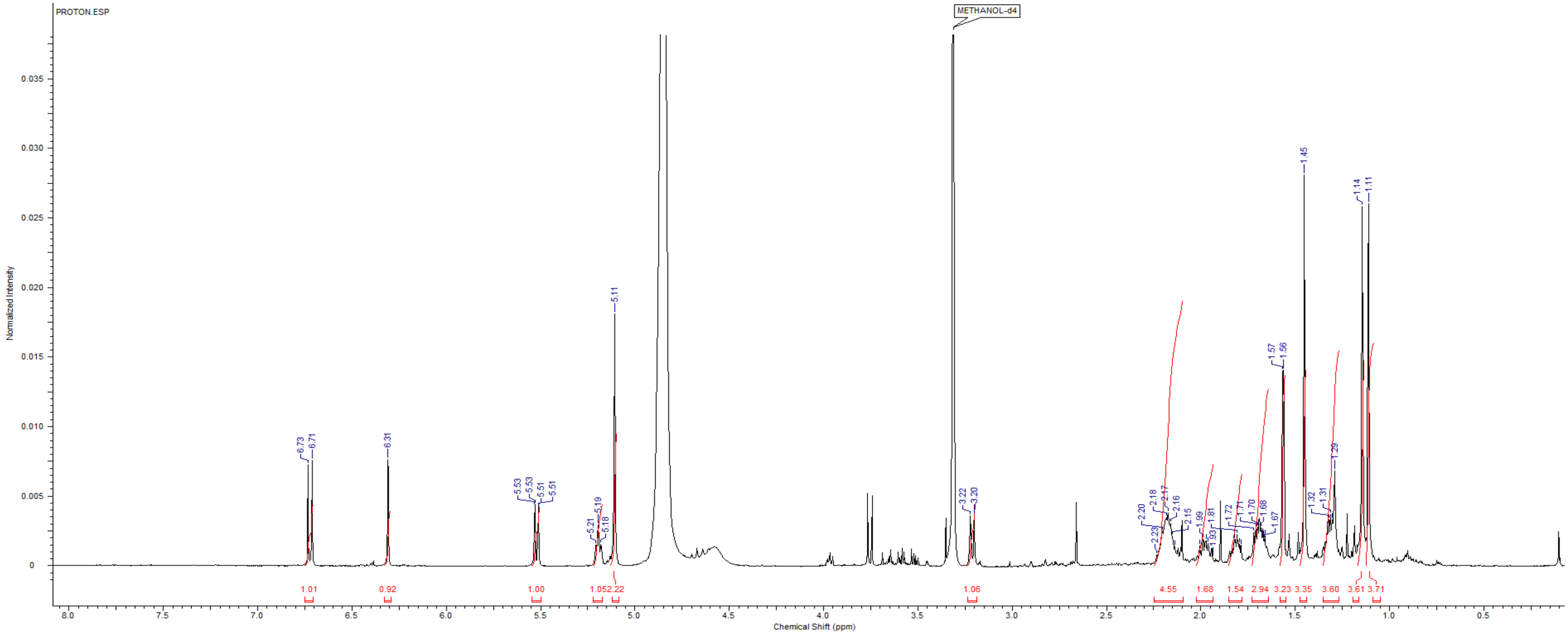

Figure S43. ^{1}H-NMR (500 MHz, CD_3OD) spectrum of **5**.

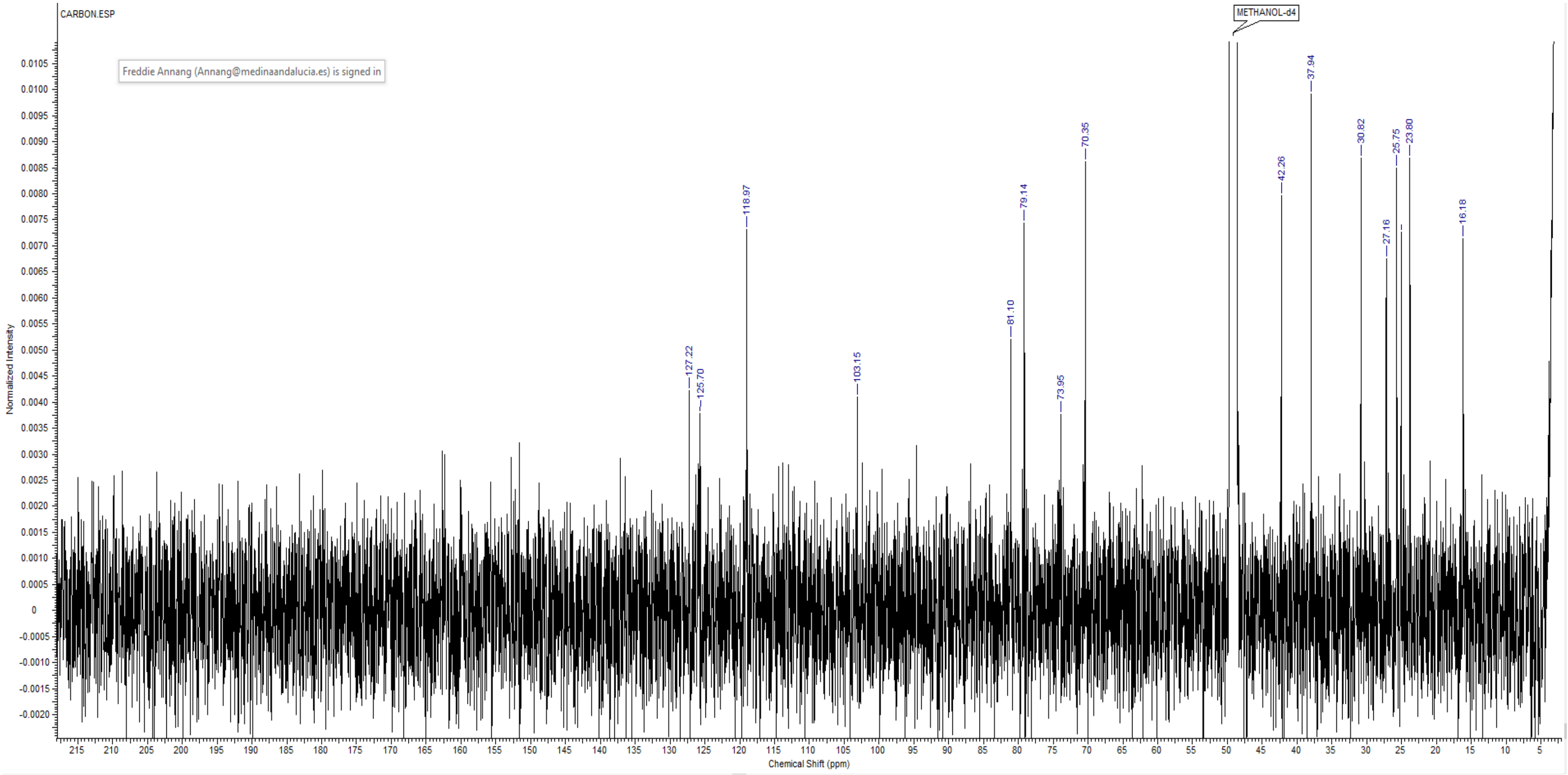

Figure S44. ^{13}C-NMR (125 MHz, CD_3OD) spectrum of **5**.

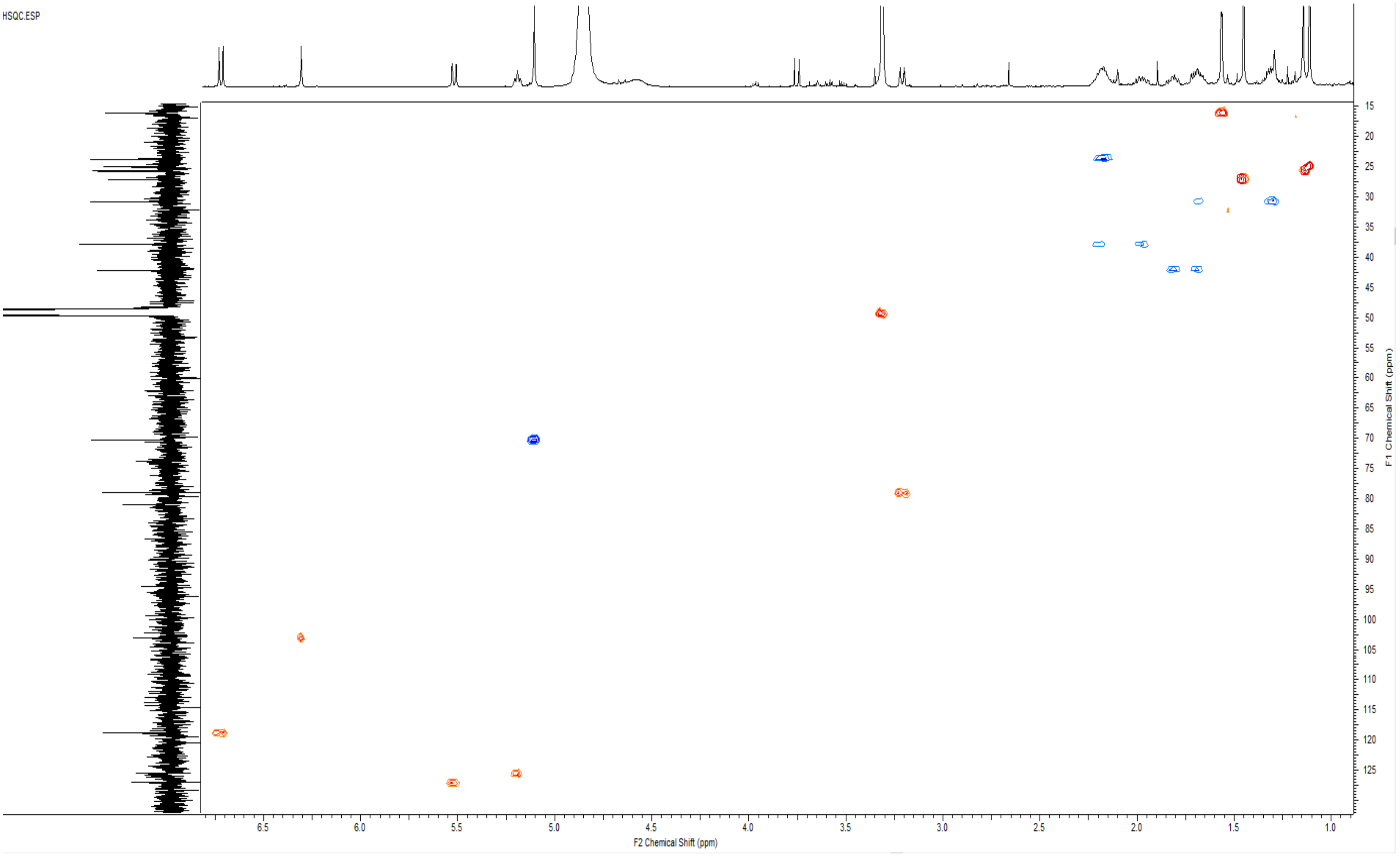

Figure S45. HSQC (CD_3OD) spectrum of **5**.

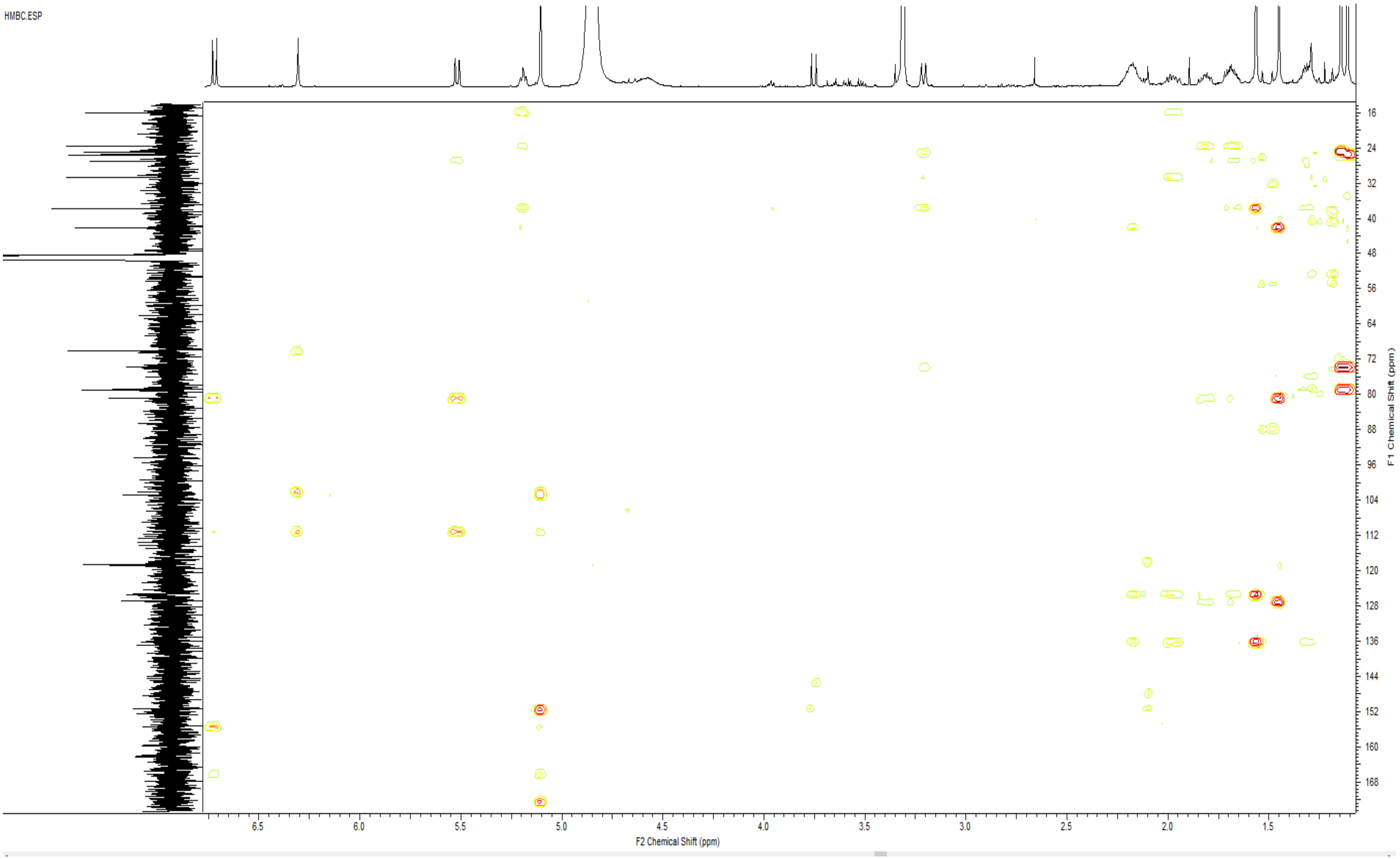

Figure S46. HMBC (CD_3OD) spectrum of **5**.

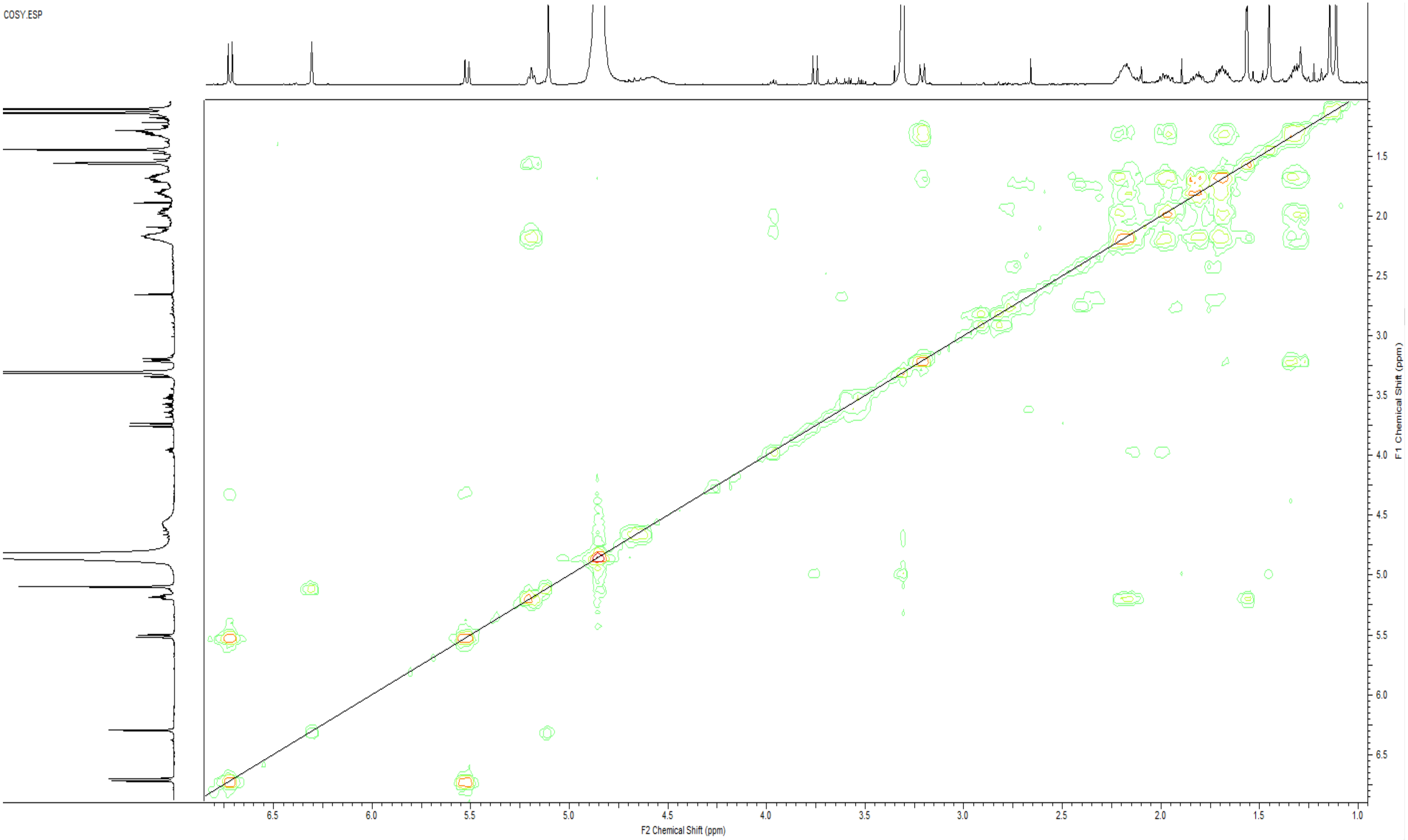

Figure S47. COSY (CD_3OD) spectrum of **5**.

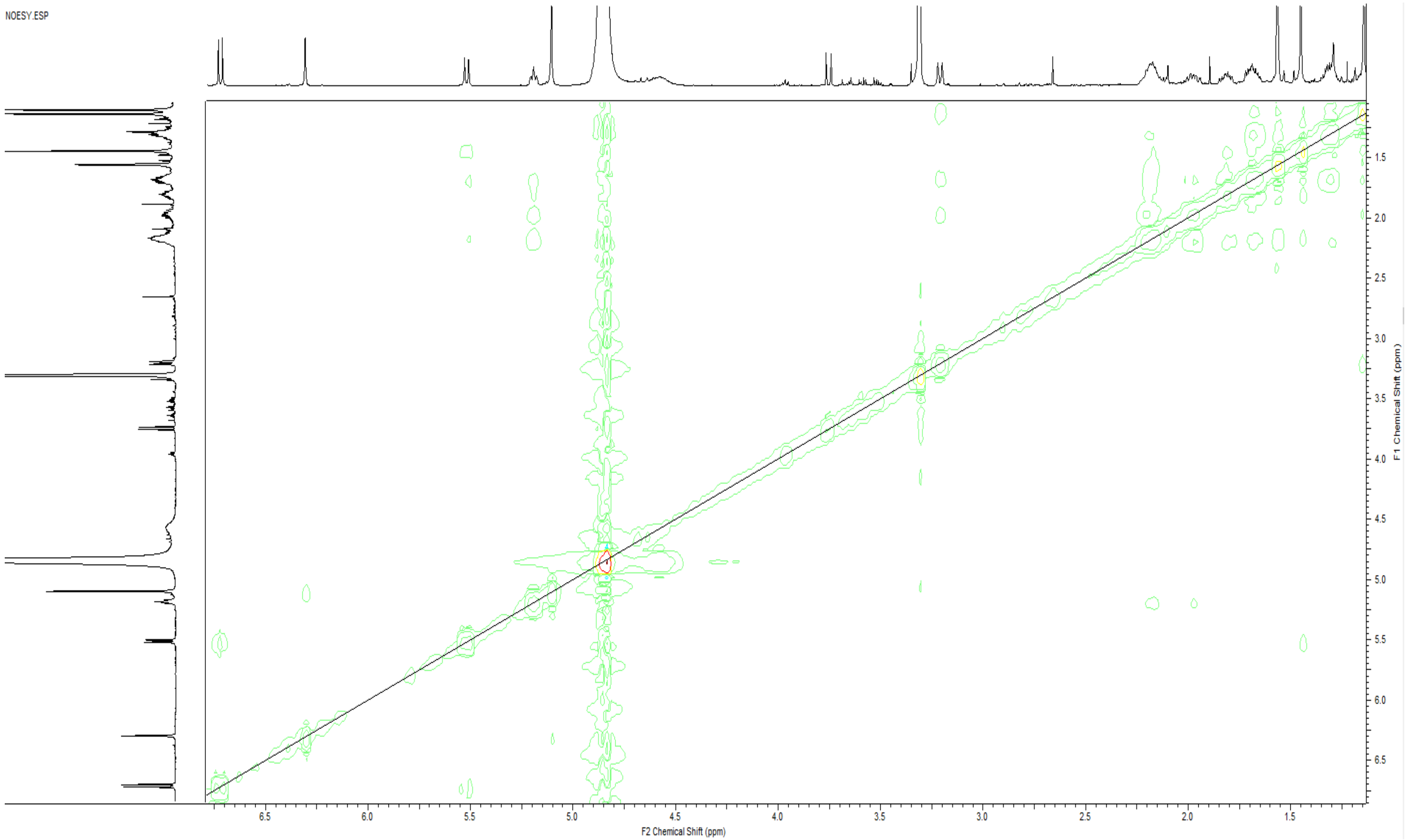

Figure S48. NOESY (CD_3OD) spectrum of **5**.

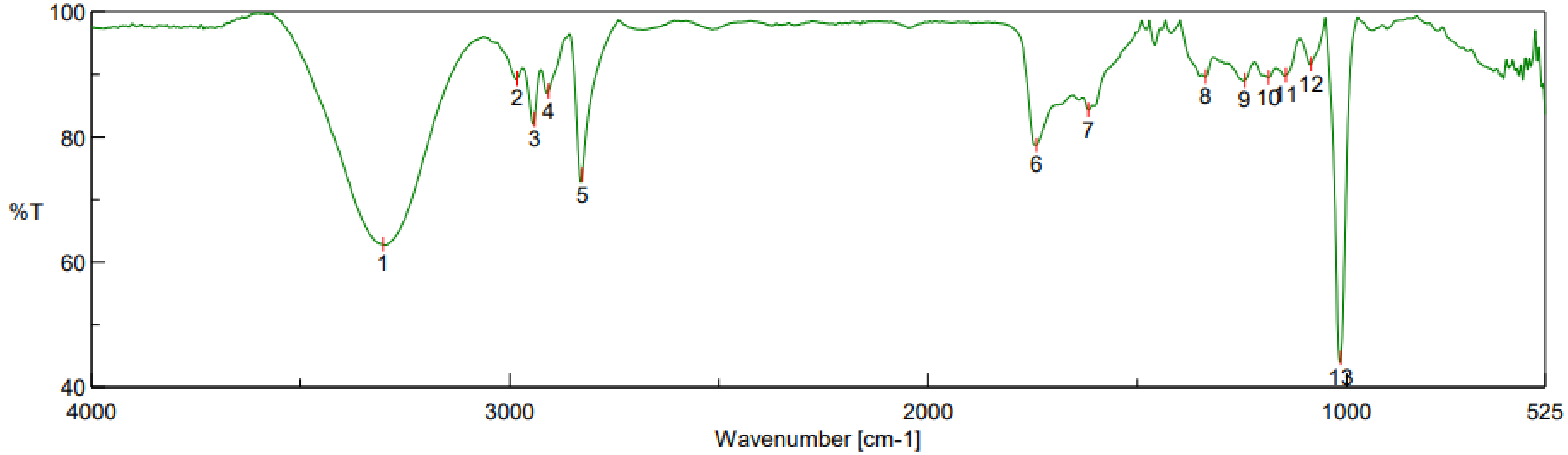

Figure S49. IR (MeOH) spectrum of **5**.

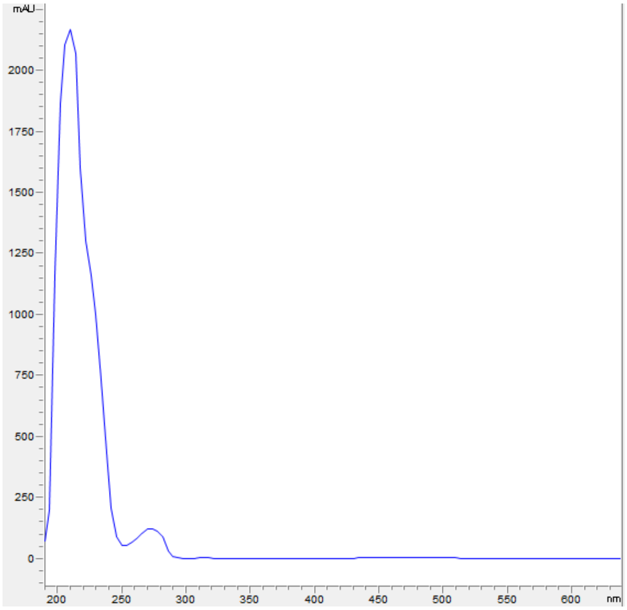

Figure S50. UV/vis (DAD) spectrum **6**.

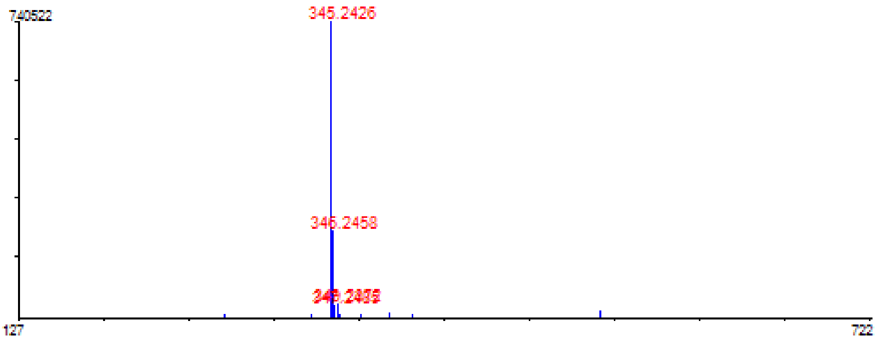

Calc. M+H+= 345.2425

Figure S51. ESI-TOF spectra of **6**.

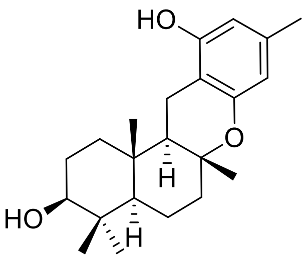

6

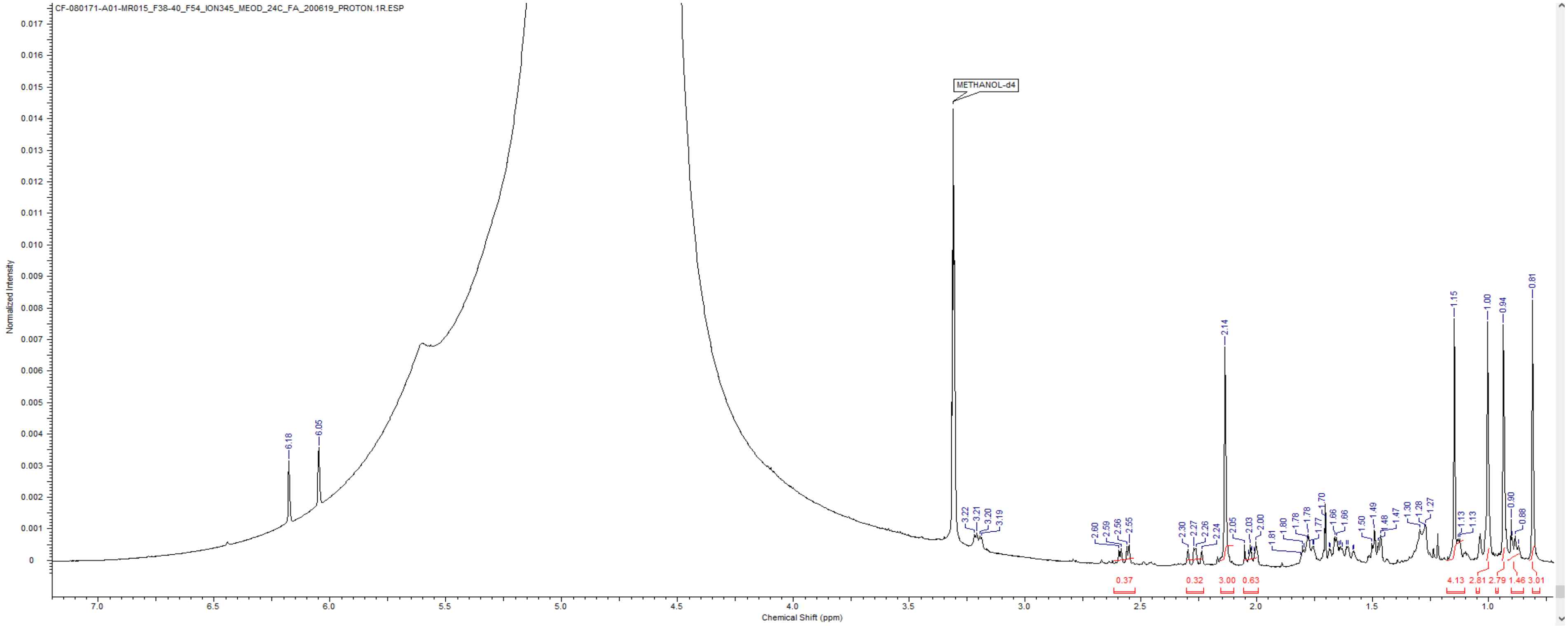

Figure S52. ^{1}H-NMR (500 MHz, CD_3OD) spectrum of **6**.

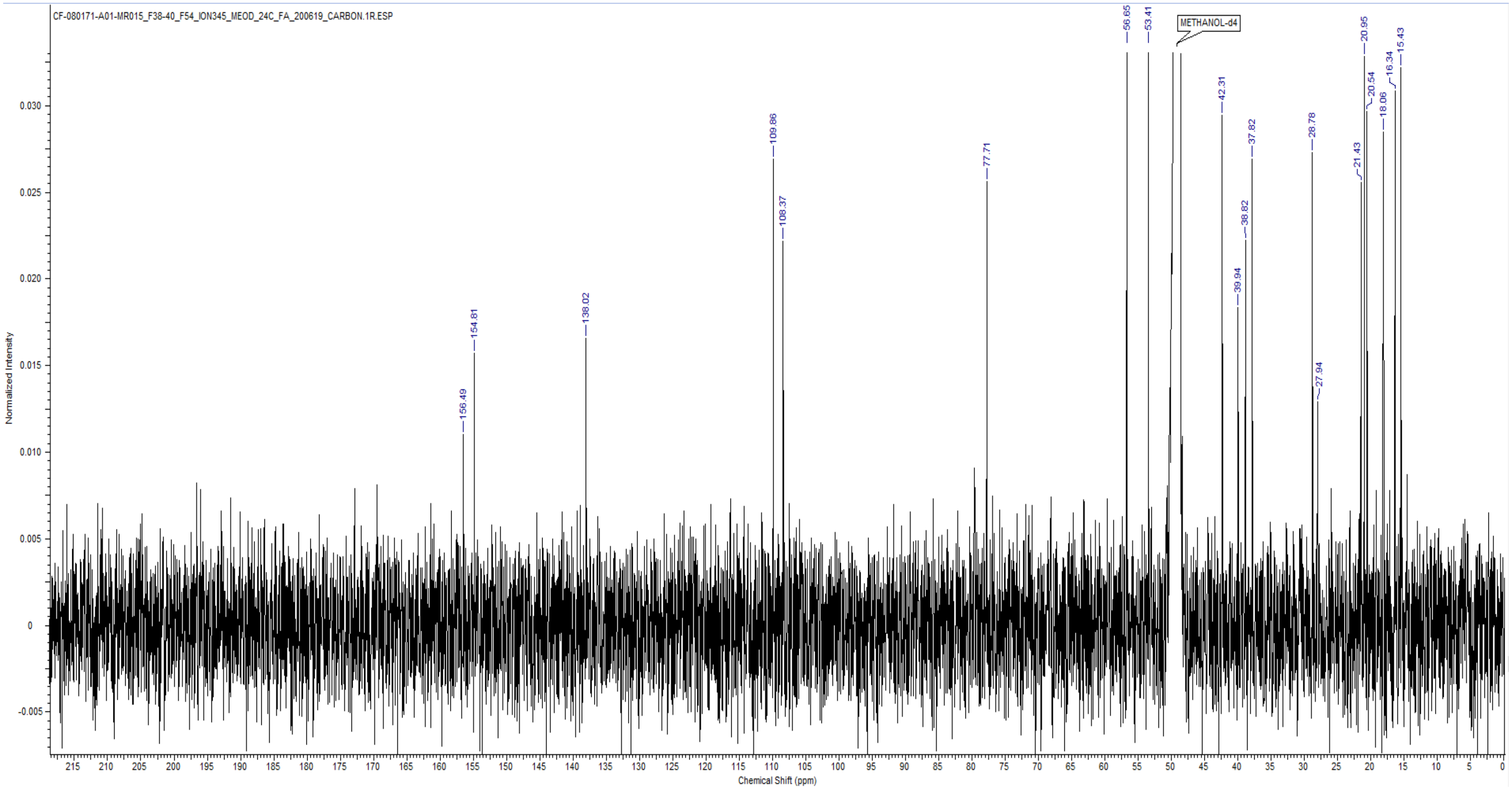

Figure S53. ^{13}C-NMR (125 MHz, CD_3OD) spectrum of **6**.

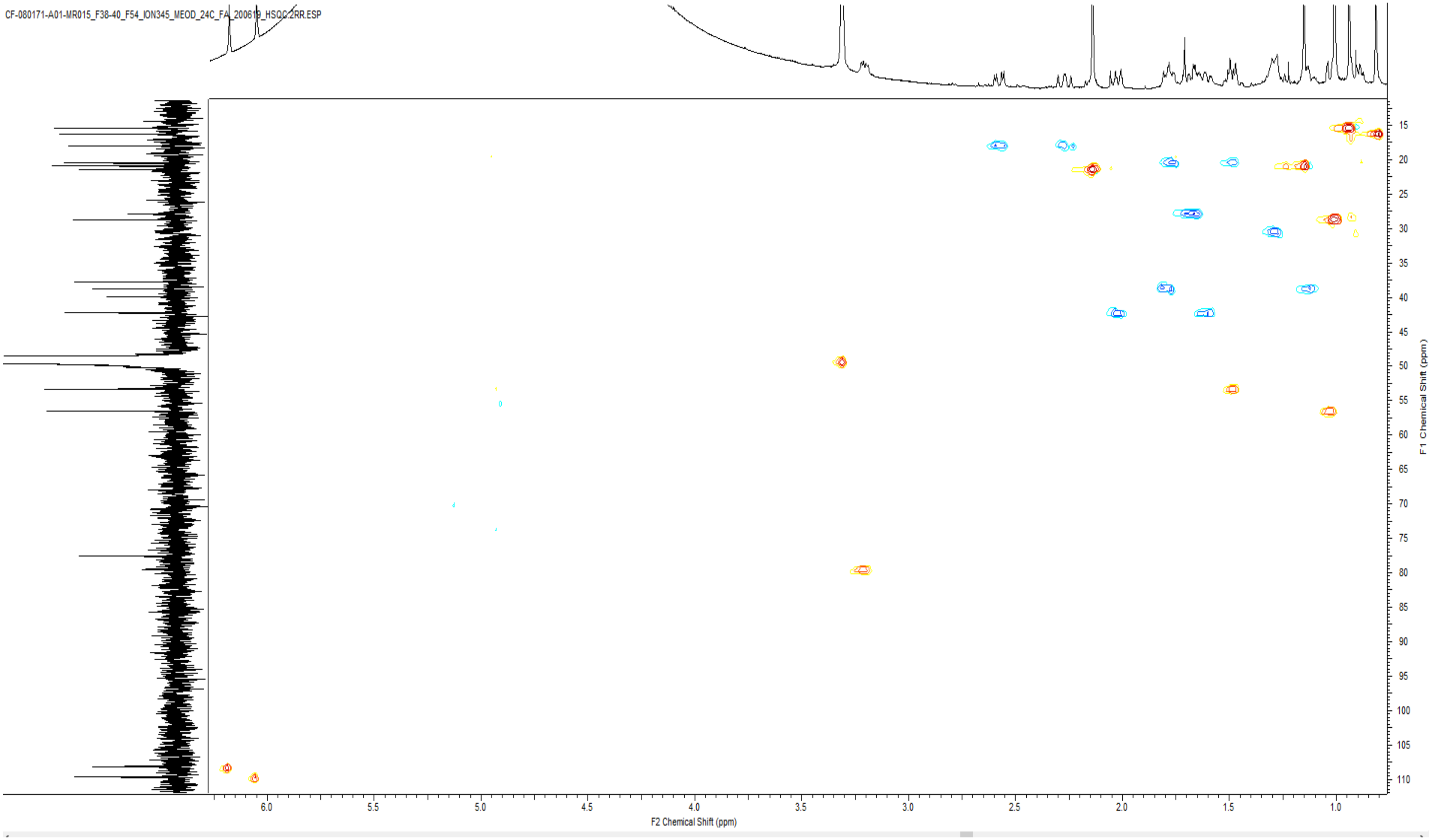

Figure S54. HSQC (CD_3OD) spectrum of **6**.

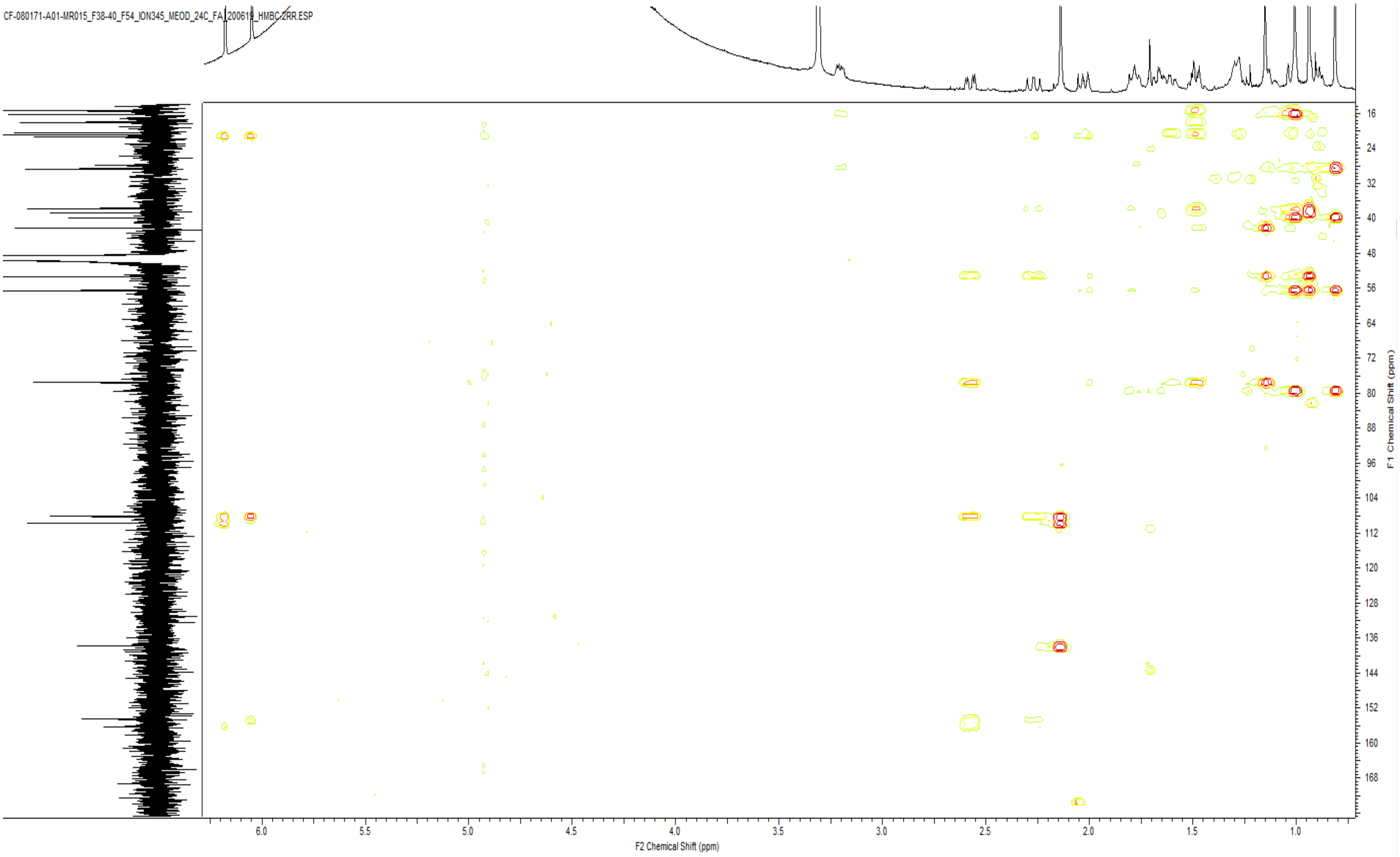

Figure S55. HMBC (CD_3OD) spectrum of **6**.

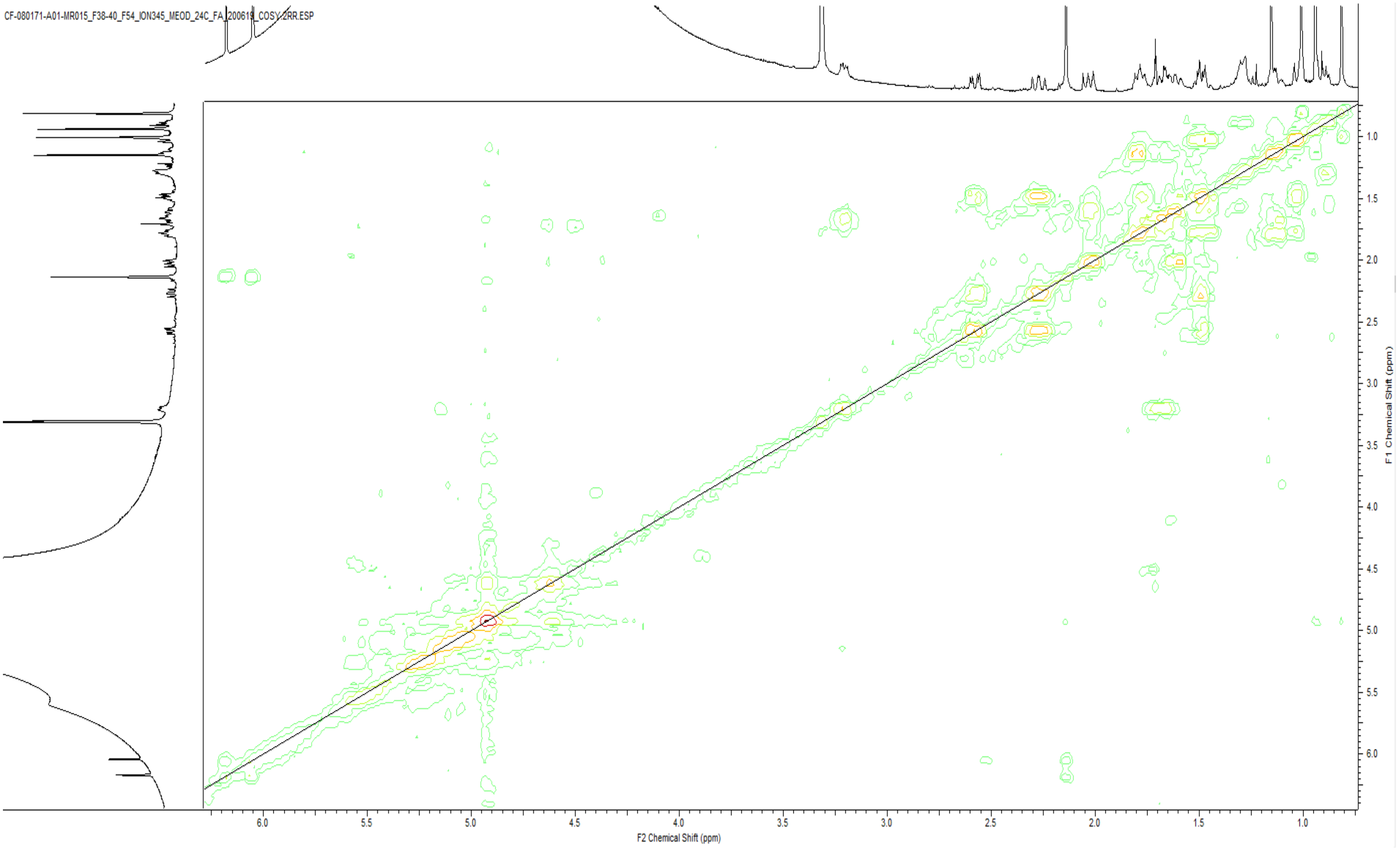

Figure S56. COSY (CD_3OD) spectrum of **6**.

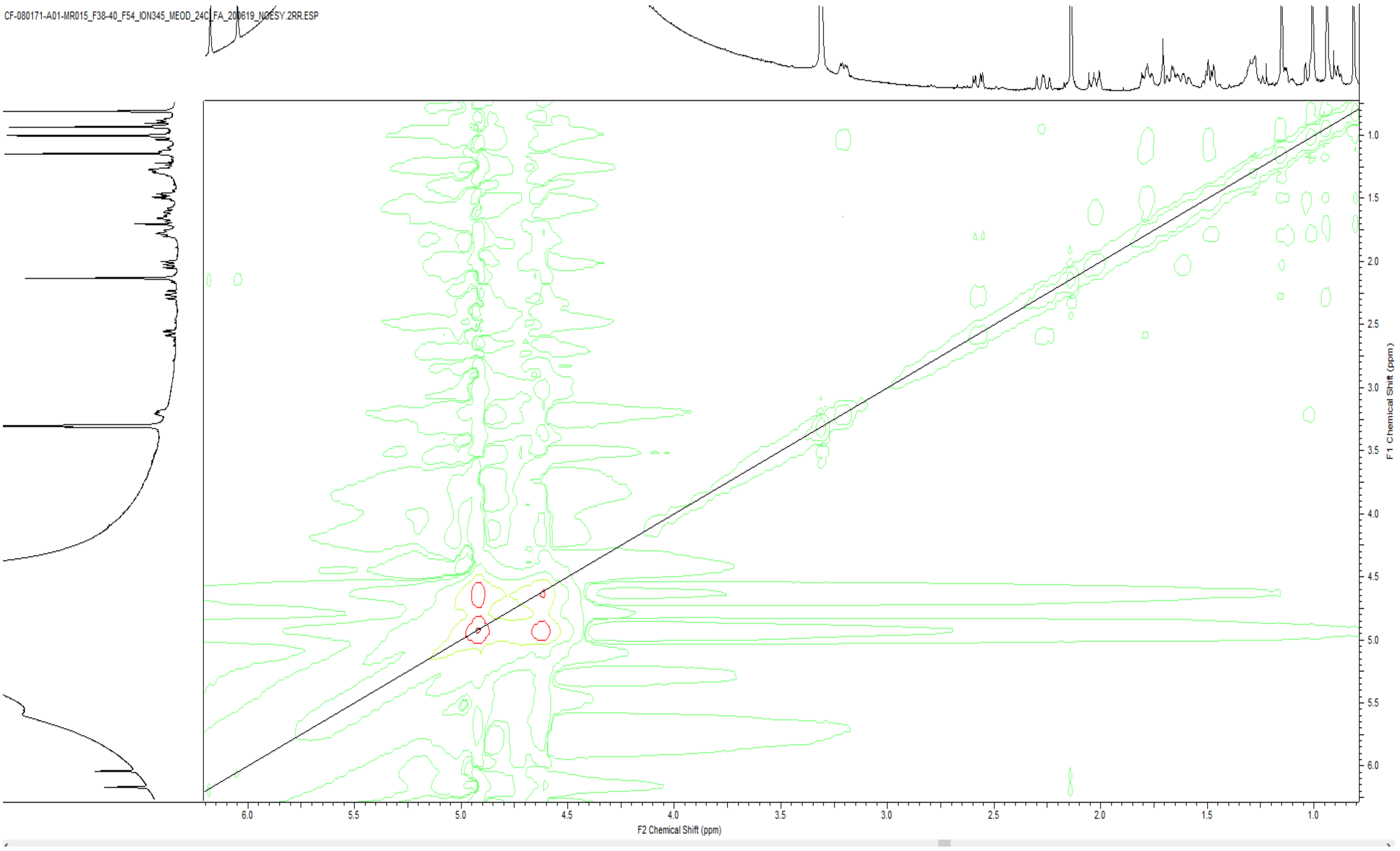

Figure S57. NOESY (CD_3OD) spectrum of **6**.

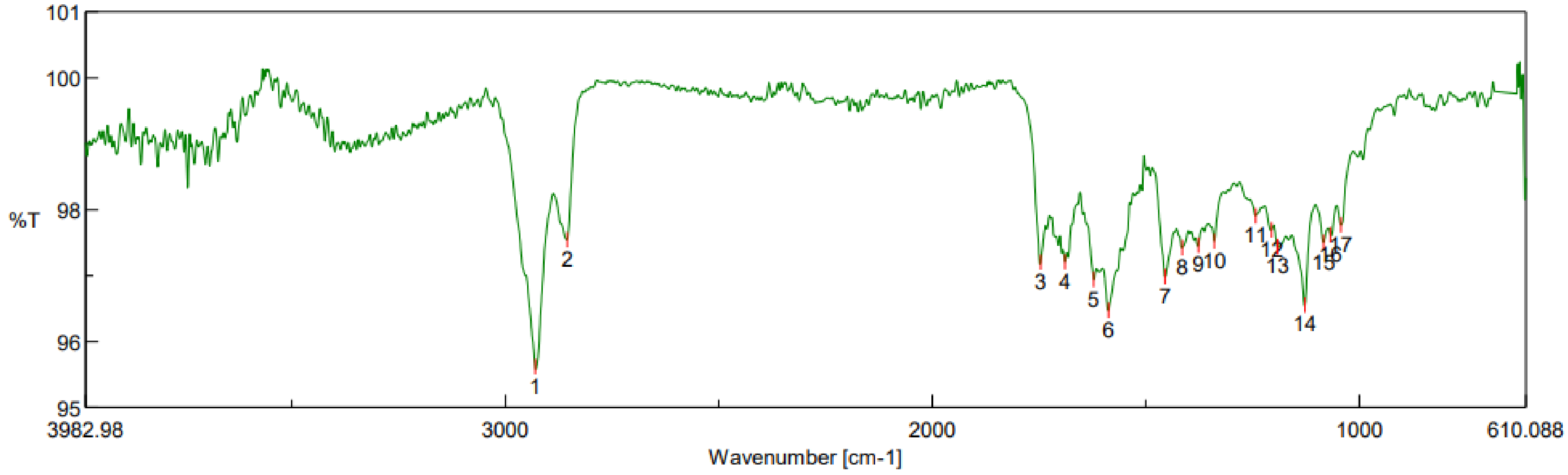

Figure S58. IR (MeOH) spectrum of **6**.

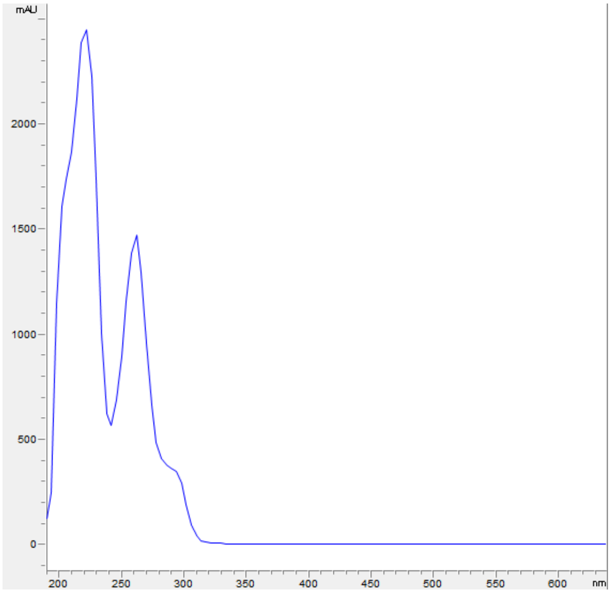

Figure S59. UV/vis (DAD) spectrum **7**.

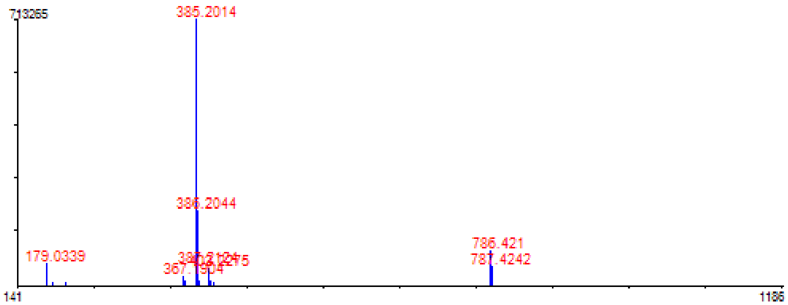

Calc. M+H$^+$= 385.2010

Calc. 2M+NH$_4$$^+$= 786.4212

Figure S60. ESI-TOF spectra of **7**.

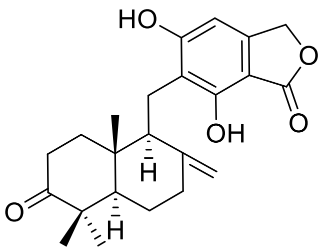

7

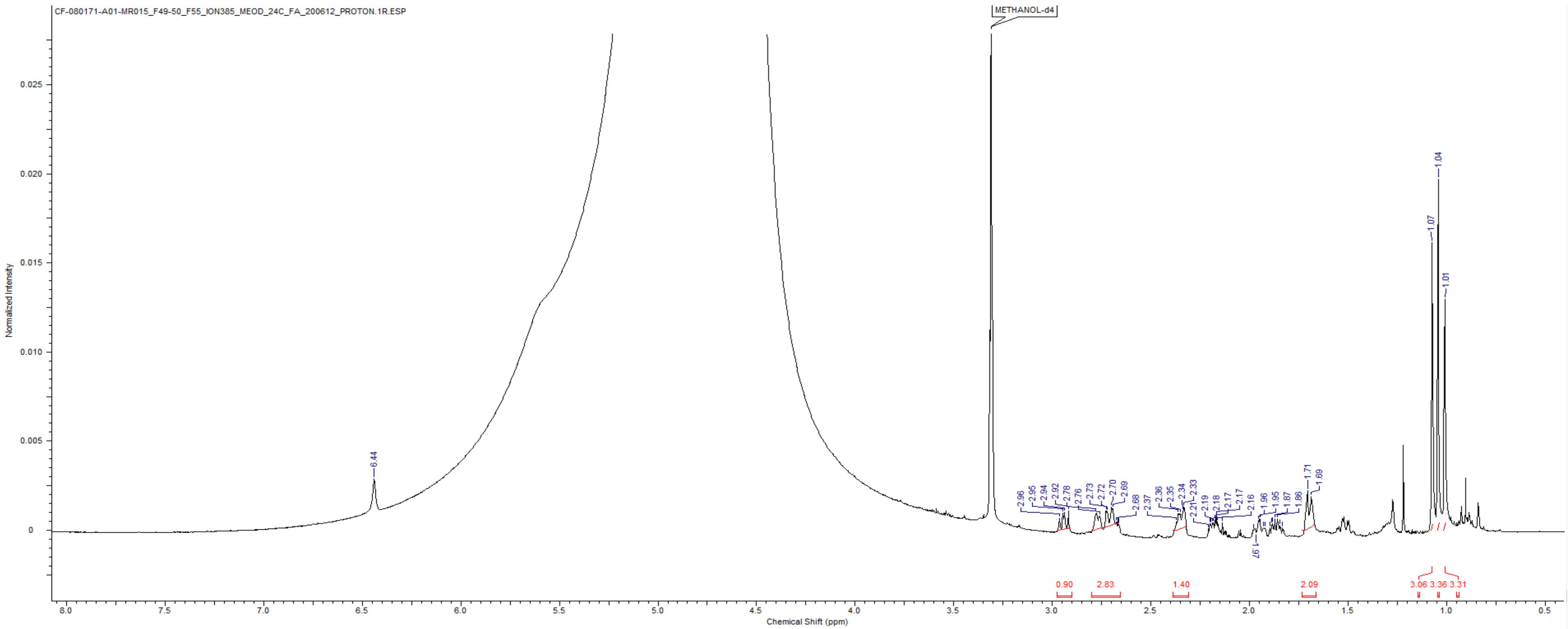

Figure S61. ^{1}H-NMR (500 MHz, CD_3OD) spectrum of **7**.

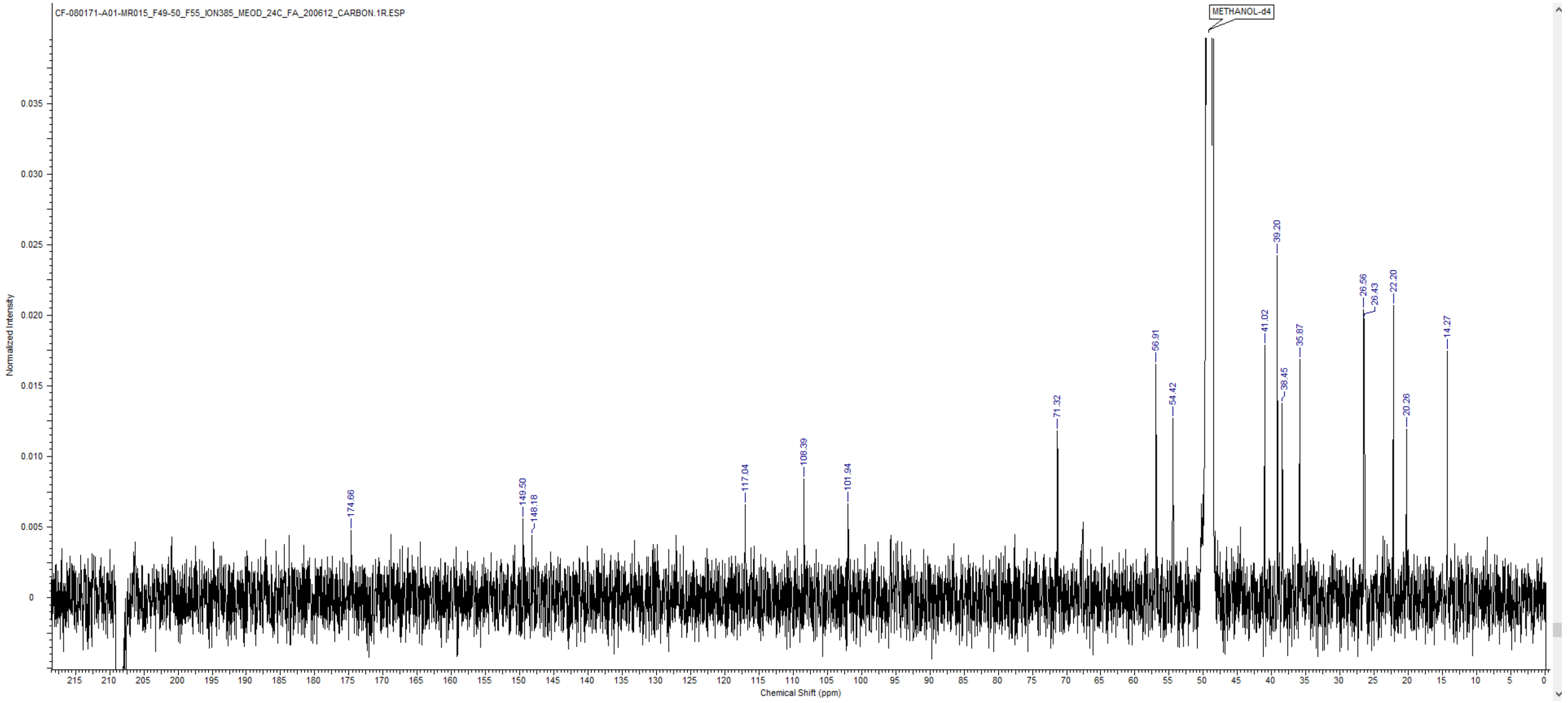

Figure S62. ^{13}C-NMR (125 MHz, CD_3OD) spectrum of **7**.

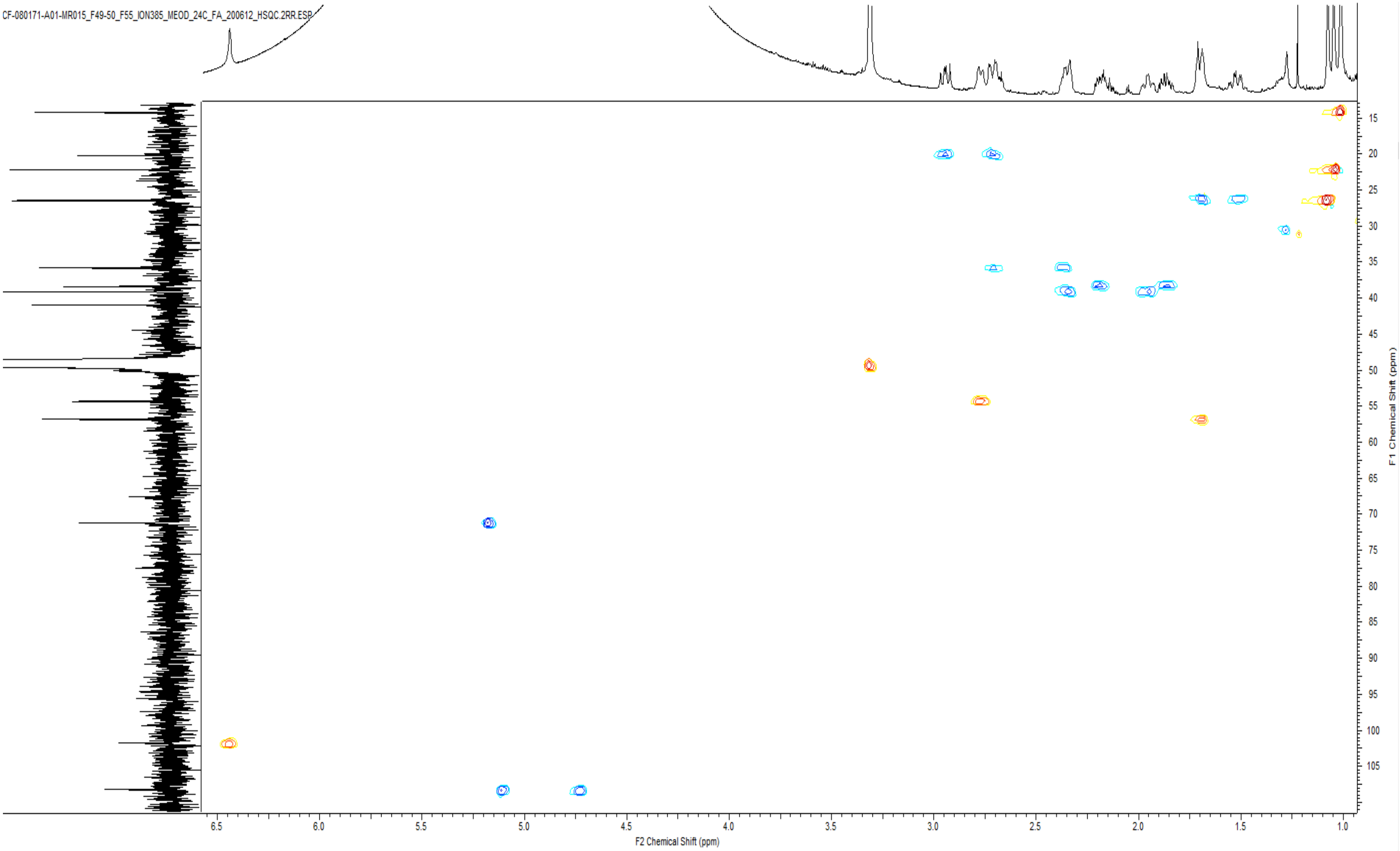

Figure S63. HSQC (CD_3OD) spectrum of **7**.

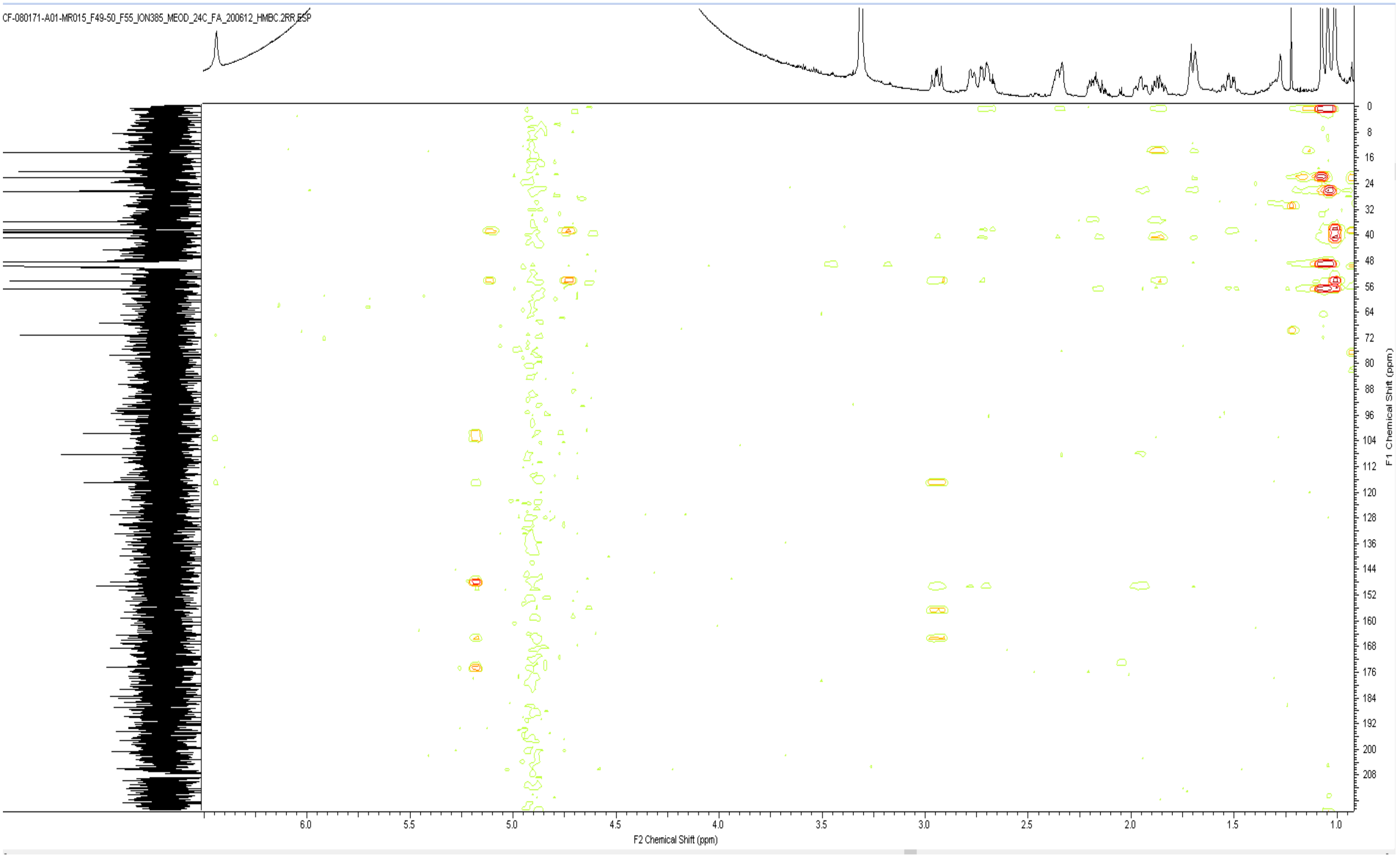

Figure S64. HMBC (CD_3OD) spectrum of **7**.

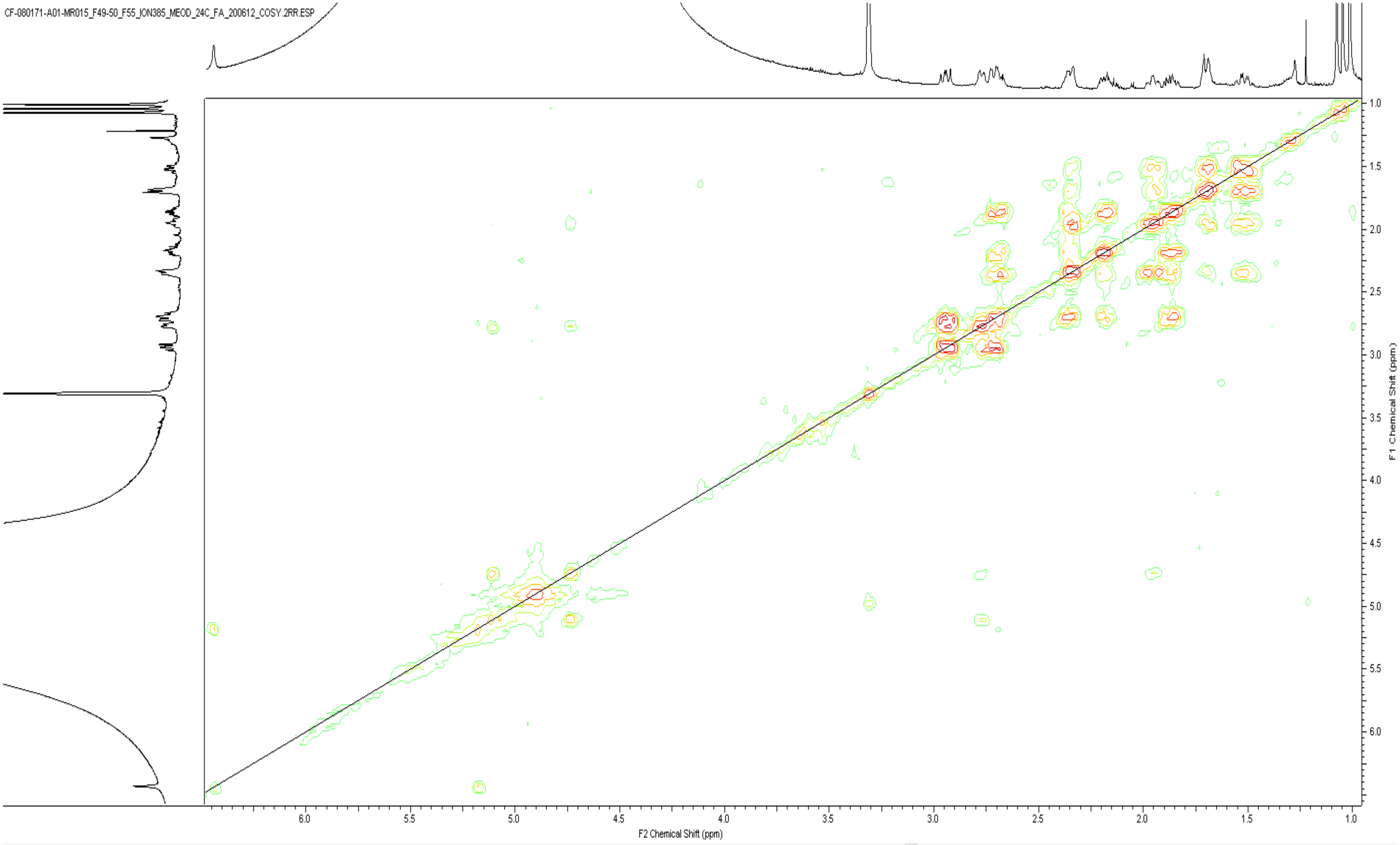

Figure S65. COSY (CD_3OD) spectrum of **7**.

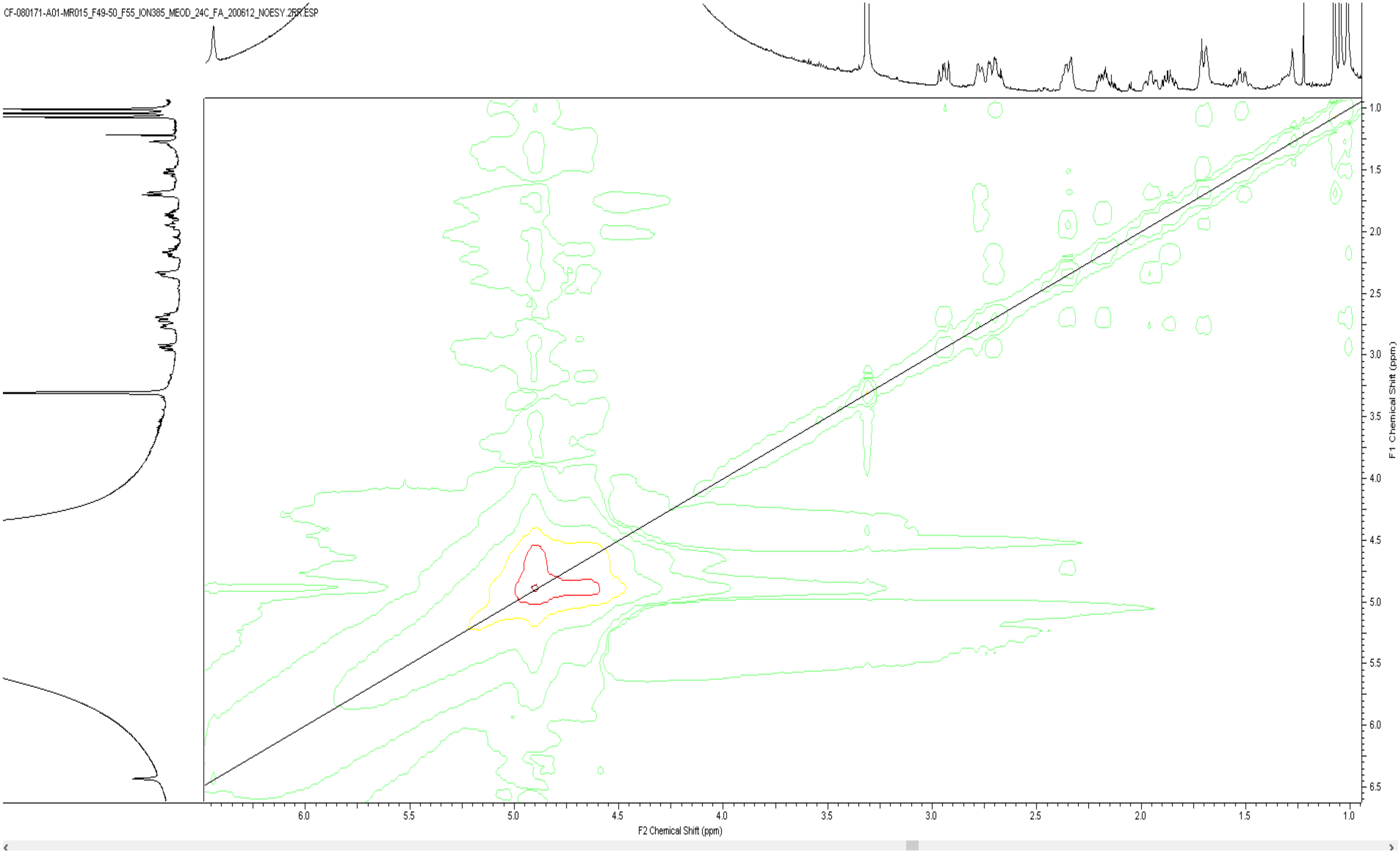

Figure S66. NOESY (CD_3OD) spectrum of **7**.

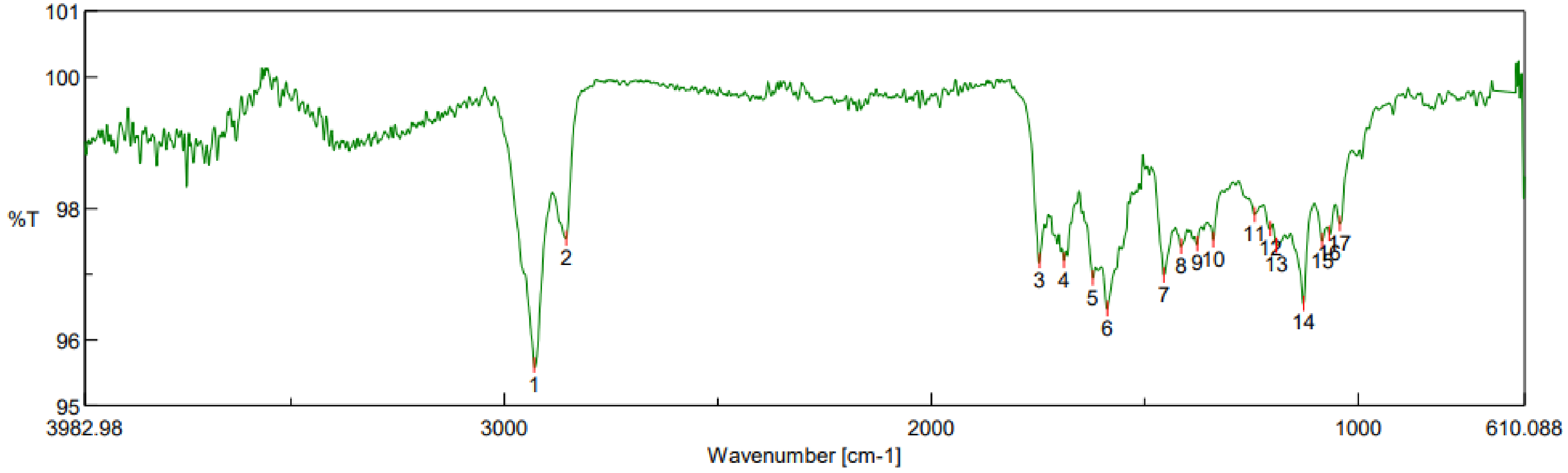

Figure S67. IR (MeOH) spectrum of **7**.

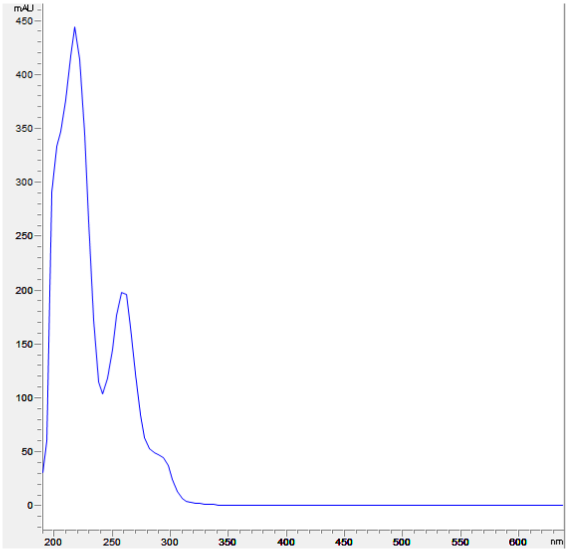

Figure S68. UV/vis (DAD) spectrum **8**.

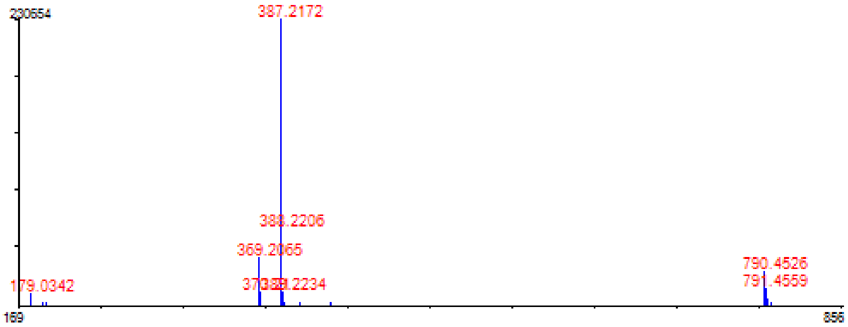

Calc. M+H$^+$= 387.2166

Calc. 2M+NH$_4$$^+$= 790.4525

Figure S69. ESI-TOF spectra of **8**.

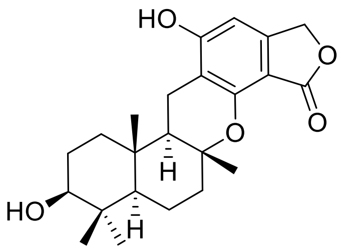

8

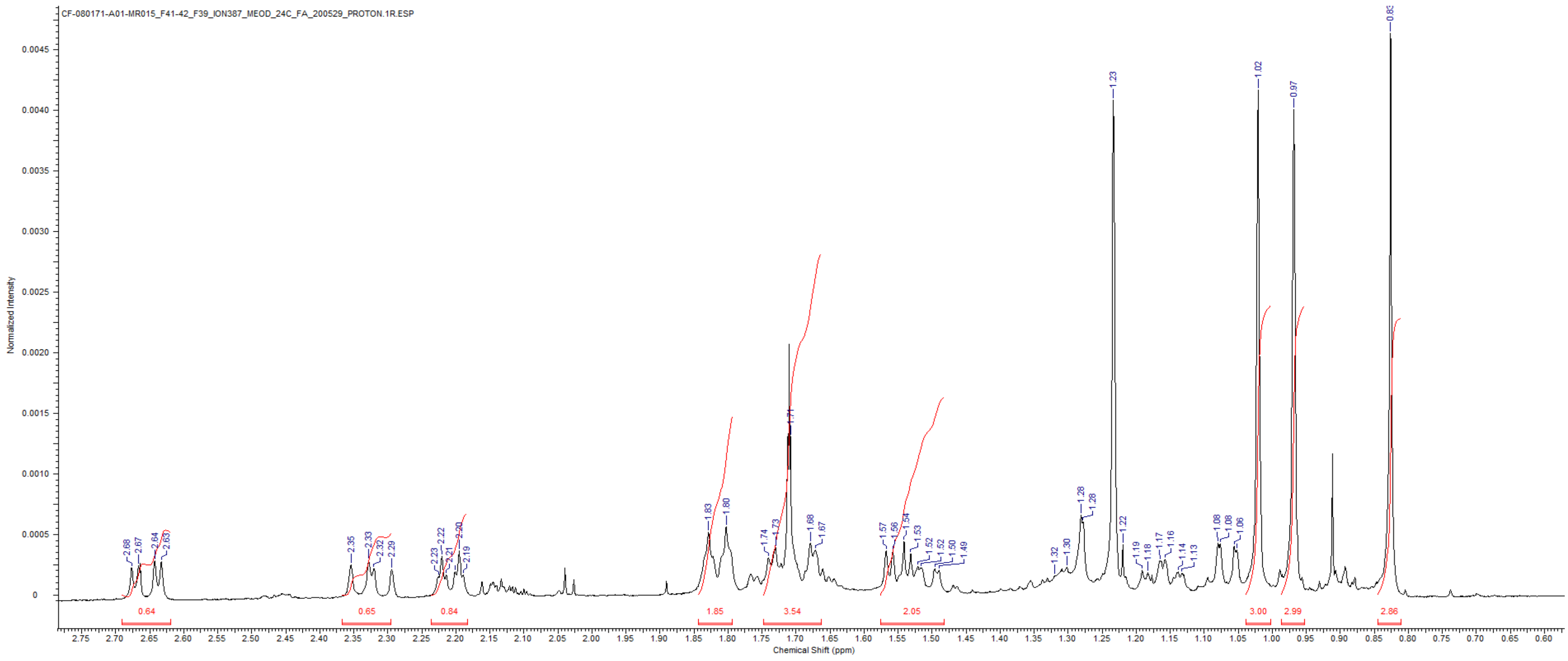

Figure S70. ^{1}H-NMR (500 MHz, CD_3OD) spectrum of **8**.

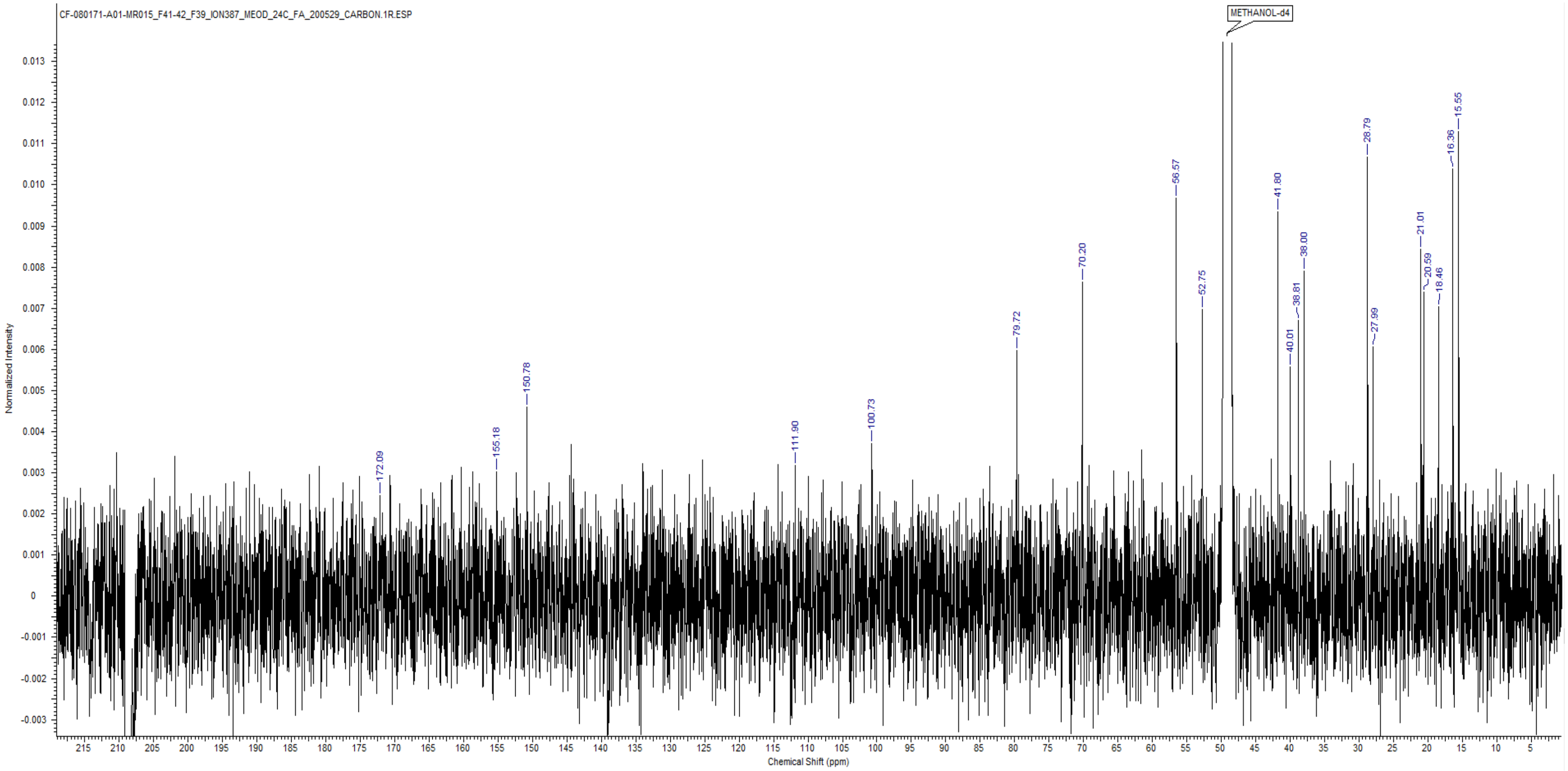

Figure S71. ^{13}C-NMR (125 MHz, CD_3OD) spectrum of **8**.

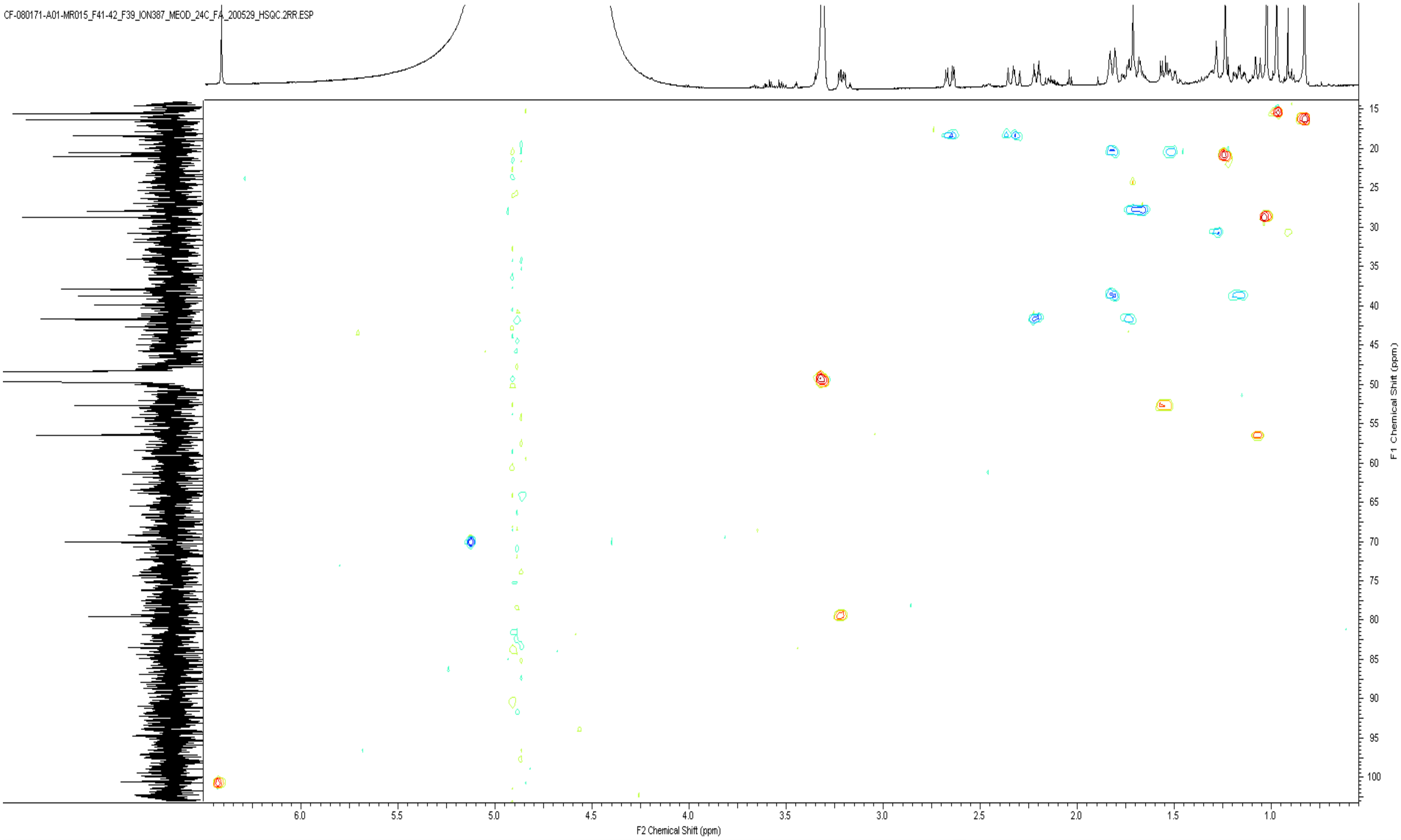

Figure S72. HSQC (CD_3OD) spectrum of **8**.

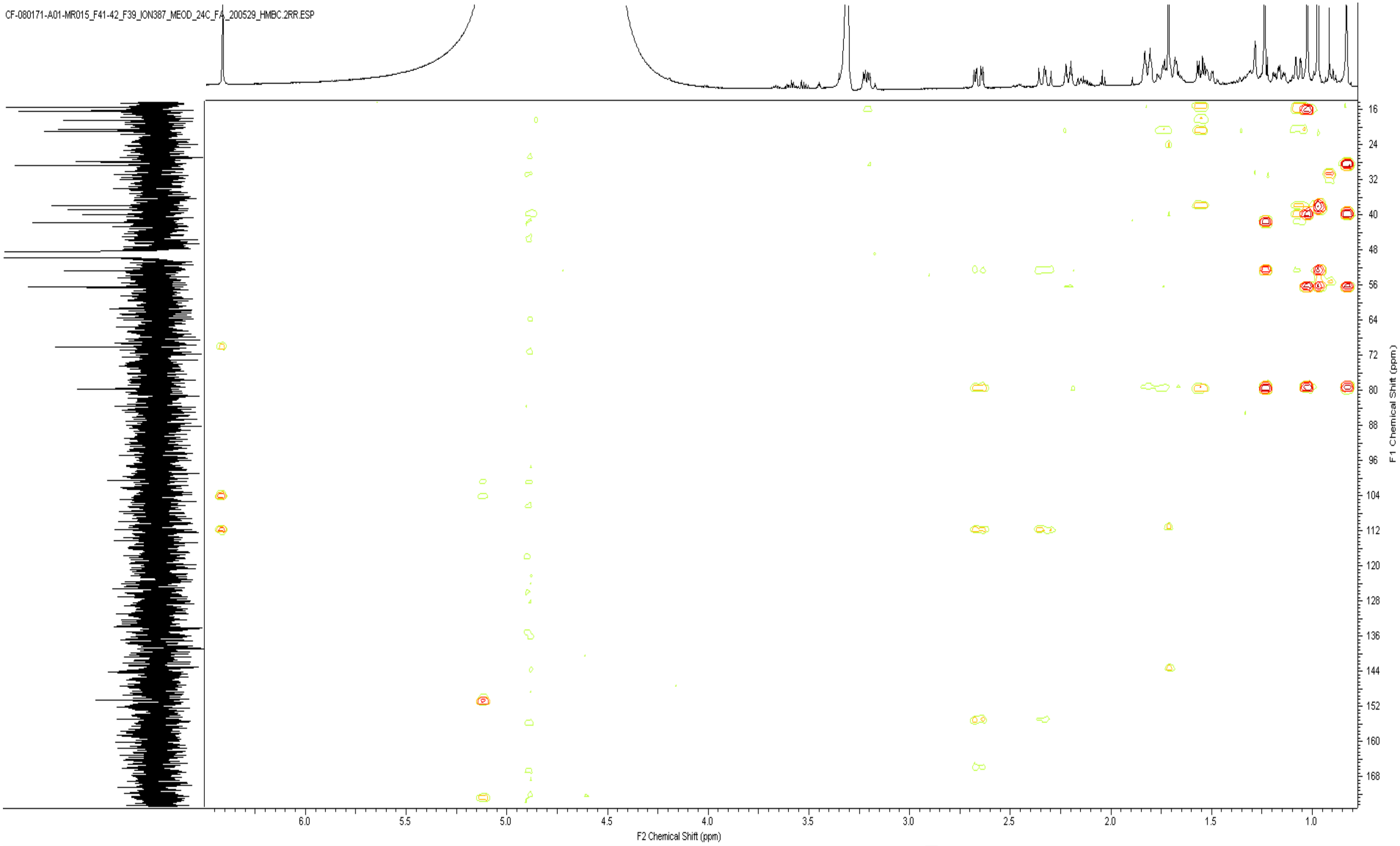

Figure S73. HMBC (CD_3OD) spectrum of **8**.

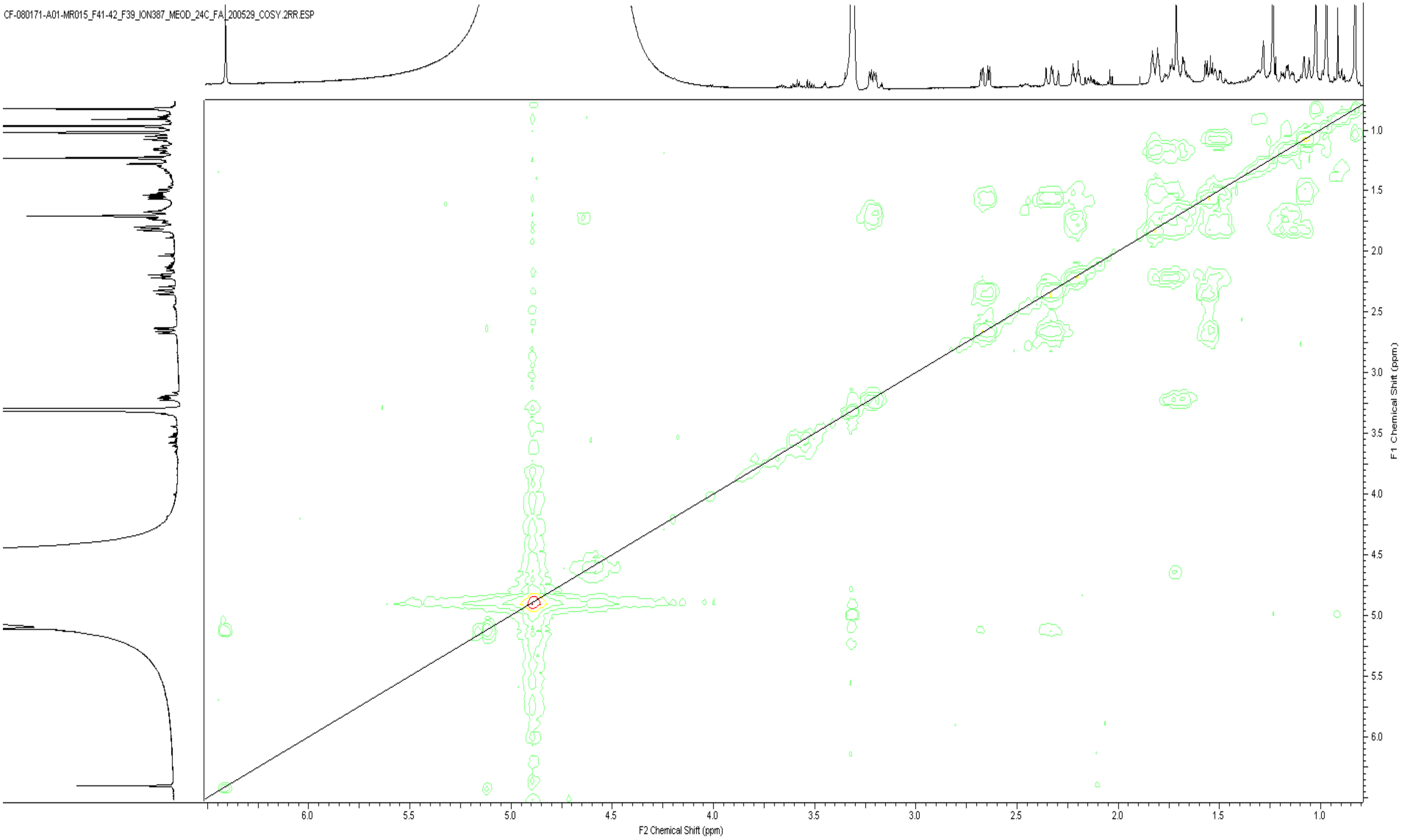

Figure S74. COSY (CD_3OD) spectrum of **8**.

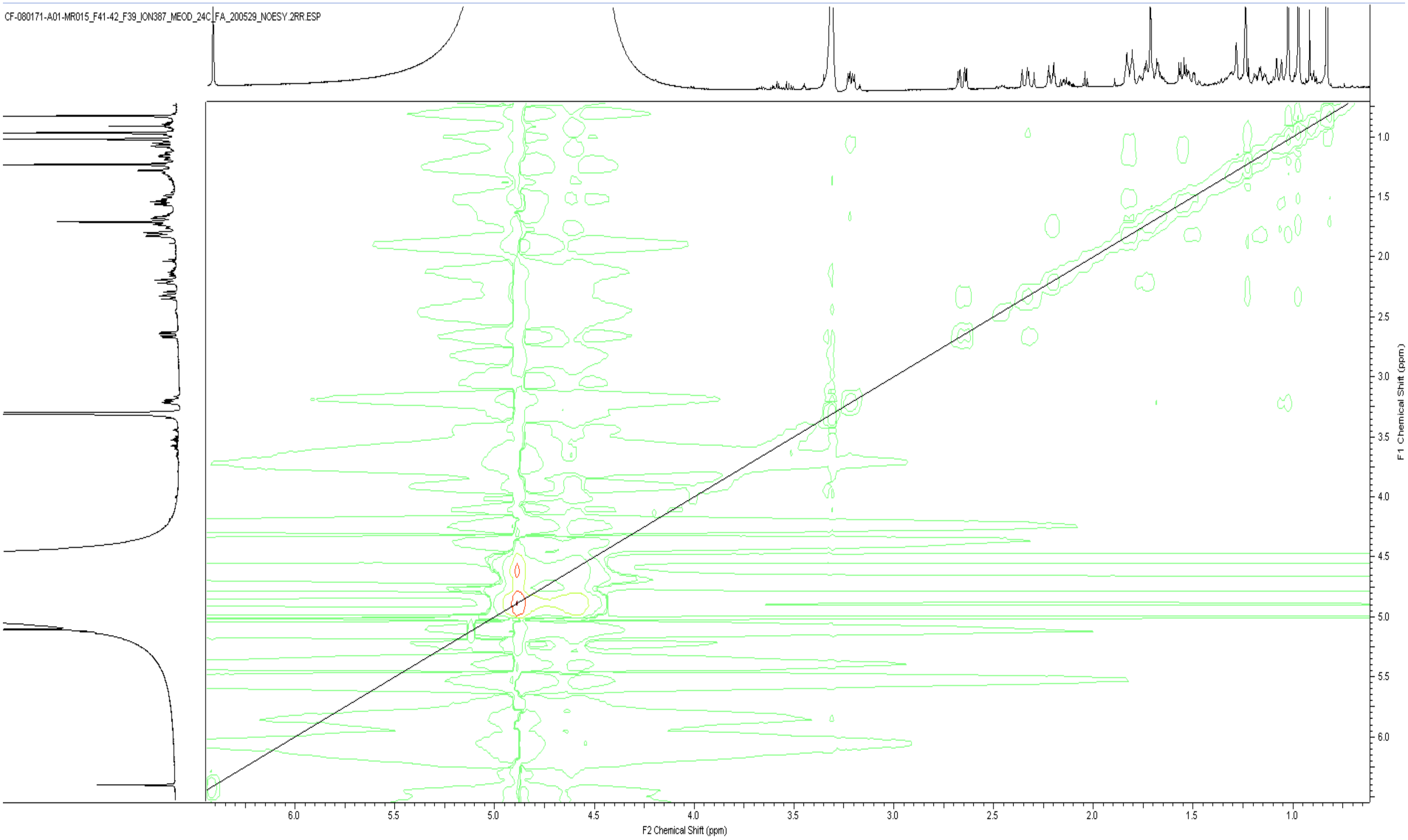

Figure S75. NOESY (CD_3OD) spectrum of **8**.

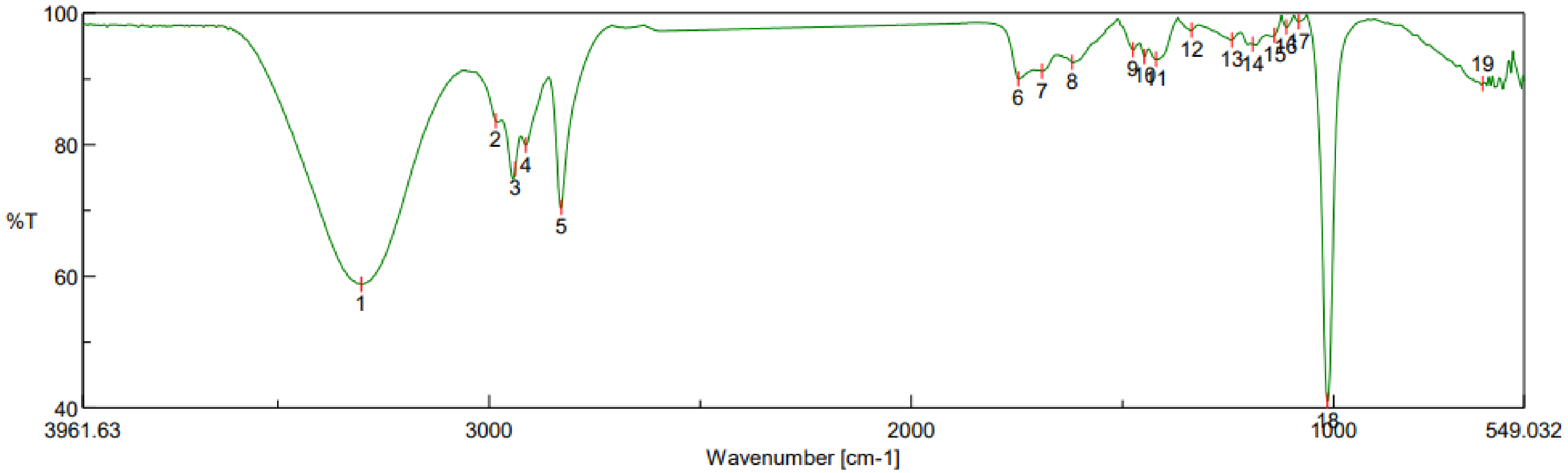

Figure S76. IR (MeOH) spectrum of **8**.

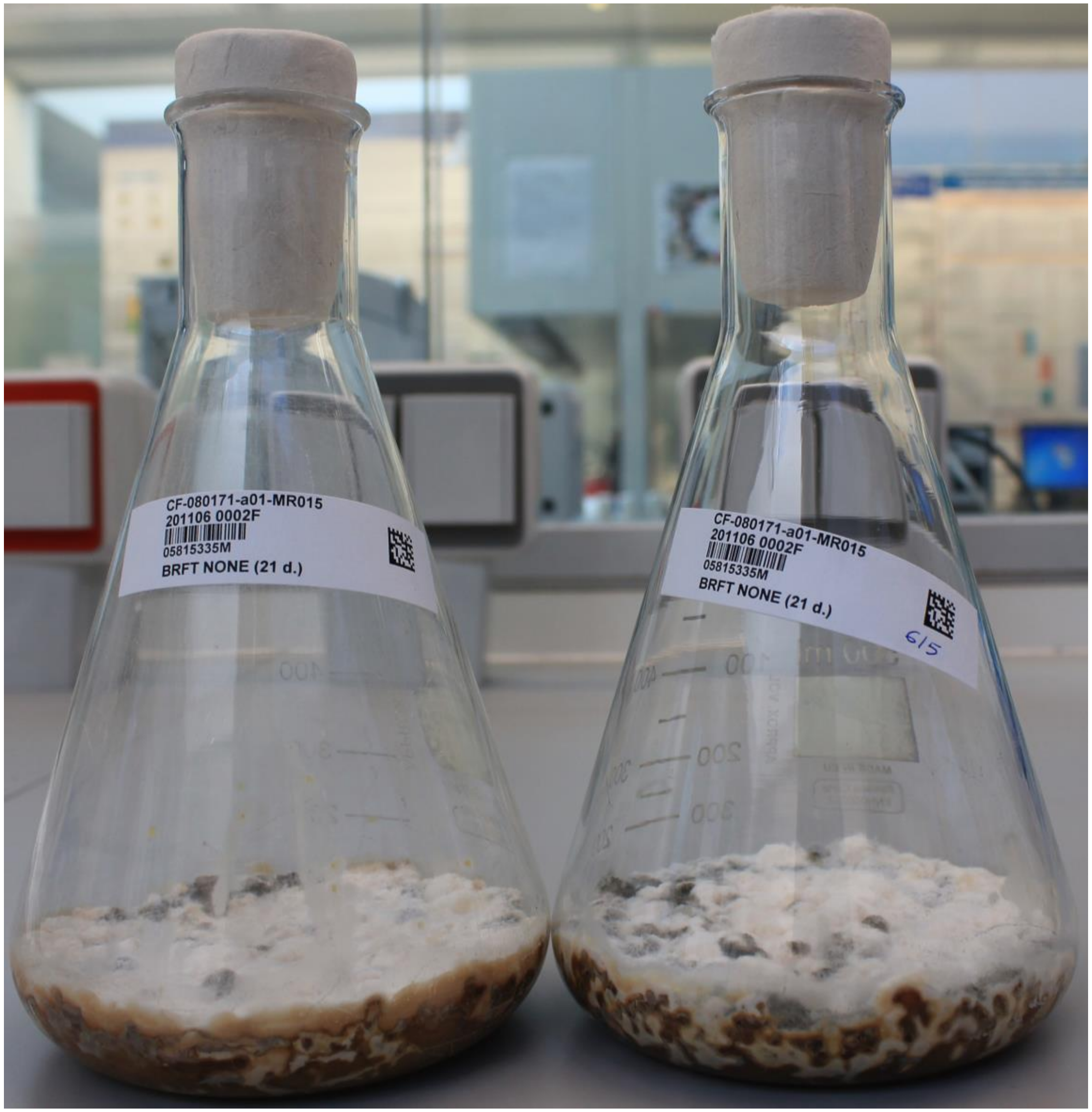

Figure S77. Picture of ***Memnoniella dichroa*** **CF-080171** grown on BRFT medium after 21 days of incubation.

Compound **1**

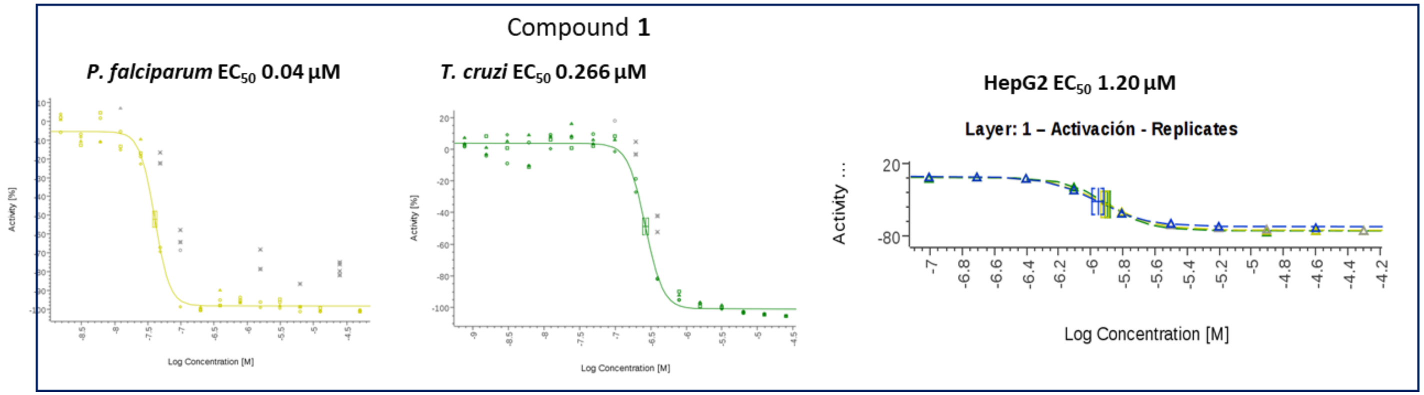

Compound **2**

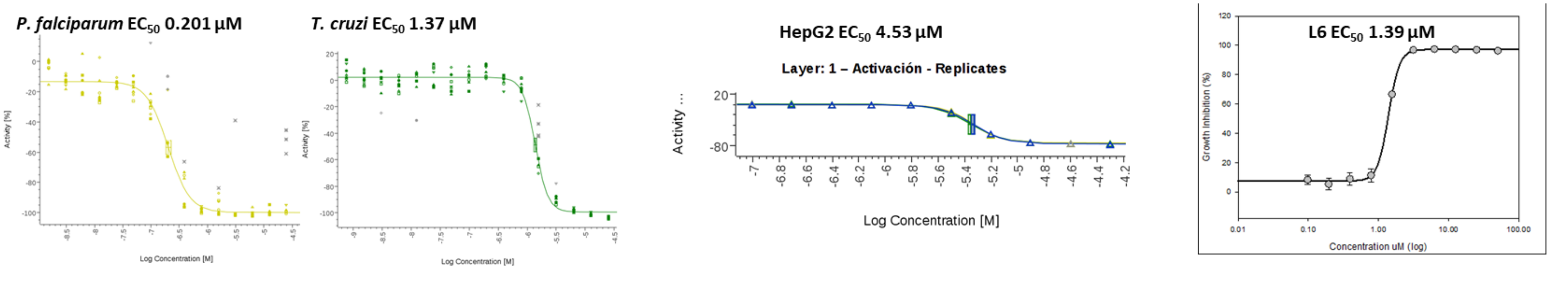

Compound **3**

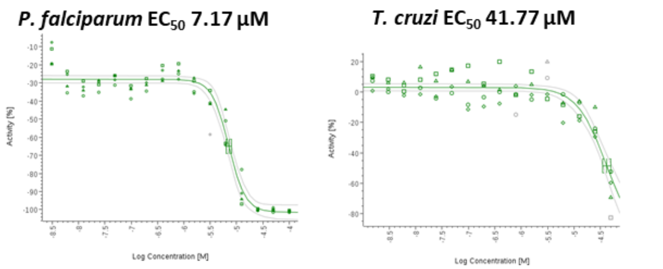

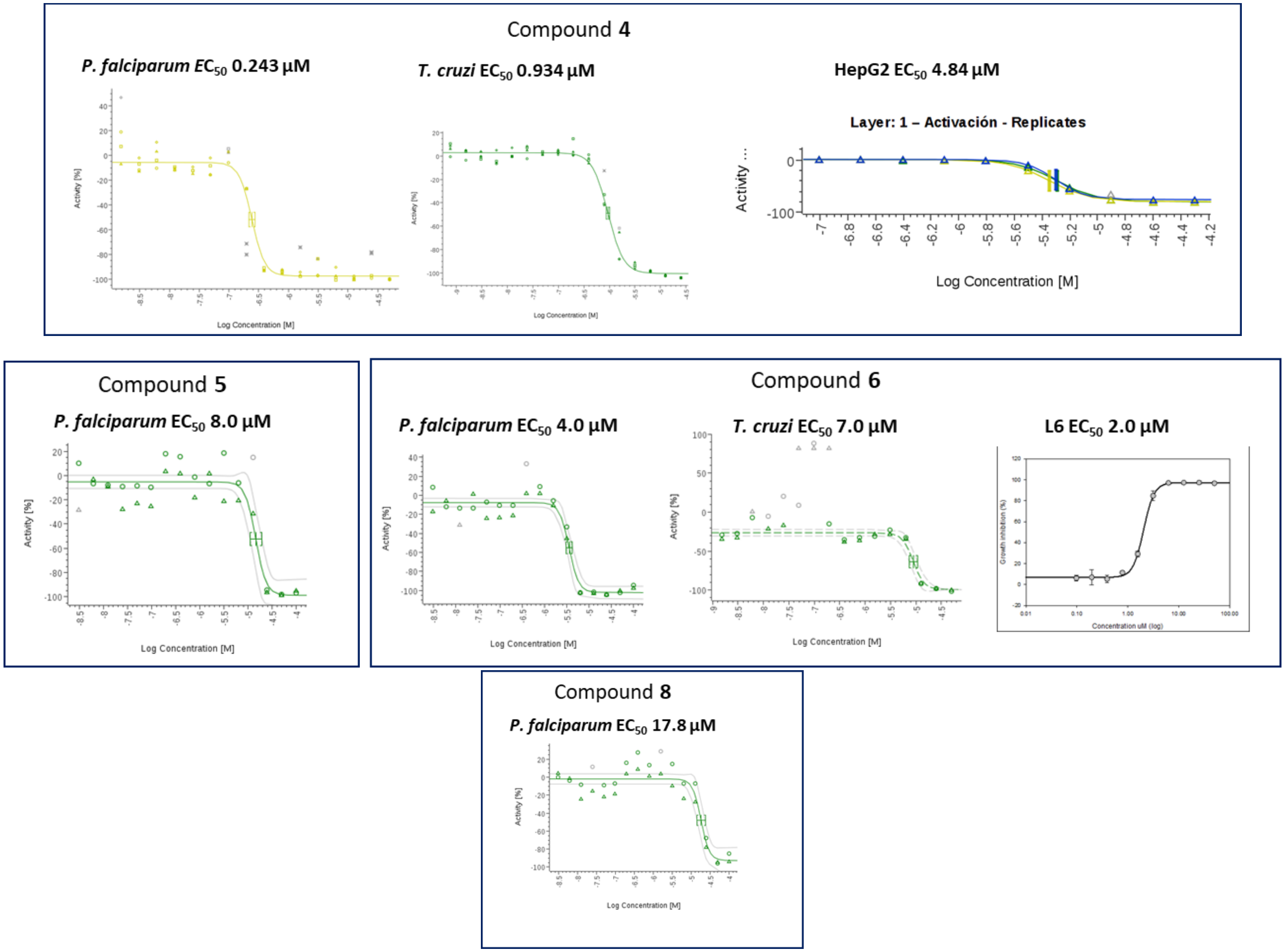

Figure S78. EC_{50} curves in the cases of the compound which could be determined for the concentration ranges tested.
